# Molecular evolution of the ATP-binding cassette subfamily G member 2 gene subfamily and its paralogs in birds

**DOI:** 10.1186/s12862-020-01654-z

**Published:** 2020-07-14

**Authors:** Shengchao Ma, Hehe Liu, Wenqiang Sun, Ahsan Mustafa, Yang Xi, Fajun Pu, Yanying Li, Chunchun Han, Lili Bai, He Hua

**Affiliations:** 1 Farm Animal Genetic Resources Exploration and Innovation Key Laboratory of Sichuan Province, Sichuan Agricultural University, Chengdu, Sichuan 611130 P.R. China; 2 Institute of Animal Nutrition, Key Laboratory for Animal Disease-Resistance Nutrition of China, Ministry of Education, Sichuan Agricultural University, Chengdu, P.R. China

**Keywords:** Birds, *ABCG2*, *ABCG2-like*, Gene duplication, Gene family evolution, Phylogeny, Selection pressures, Chromosomal synteny

## Abstract

**Background:**

ATP-binding cassette (ABC) transporters are involved in the active transportation of various endogenous or exogenous substances. Two *ABCG2* gene subfamily members have been identified in birds. A detailed comparative study of the *ABCG2* and *ABCG2-like* genes aid our understanding of their evolutionary history at the molecular level and provide a theoretical reference for studying the specific functions of *ABCG2* and *ABCG2-like* genes in birds.

**Results:**

We first identified 77 *ABCG2*/*ABCG2-like* gene sequences in the genomes of 41 birds. Further analysis showed that both the nucleic acid and amino acid sequences of *ABCG2* and *ABCG2-like* genes were highly conserved and exhibited high homology in birds. However, significant differences in the N-terminal structure were found between the ABCG2 and ABCG2-like amino acid sequences. A selective pressure analysis showed that the *ABCG2* and *ABCG2-like* genes were affected by purifying selection during the process of bird evolution.

**Conclusions:**

We believe that multiple members of the *ABCG2* gene subfamily exist on chromosome 4 in the ancestors of birds. Over the long course of evolution, only the *ABCG2* gene was retained on chromosome 4 in birds. The *ABCG2-like* gene on chromosome 6 might have originated from chromosome replication or fusion. The structural differences between the N terminus of ABCG2 protein and those of ABCG2-like proteins might lead to functional differences between the corresponding genes.

## Background

The ATP-binding cassette (ABC) subfamily G member 2 (junior blood group) (*ABCG2*) gene is the second member of the G subfamily of ABC transporters and is also considered the breast cancer resistance protein (*BCRP*) gene [1, 2]. The ABCG2/BCRP protein is mainly distributed in tissues with secretory and excretory functions, such as placental/synovial trophoblasts, small and large intestinal epithelia, liver tubule membrane, canaliculi, mammary lobule and vascular endothelial cells [3, 4]. The human ABCG2 protein contains a nucleotide-binding domain (NBD) and six transmembrane domains (TMDs) [5]. The ABCG2 protein can transport substrates from intracellular fluids to extracellular interstitial fluids and is reportedly involved in various other functions [6, 7], such as the stability of stem cells [8], the steady state of tissues cells [9–11], maintaining the blood-brain barrier and the fetal blood barrier [1, 12] and reducing drug absorption, distribution and excretion [13].

The members of the ABC transporter superfamily in most mammals can be classified into seven subfamilies (from A to G) [14], and each of these subfamilies might have undergone a long evolutionary process, from single structures to half structures or ABC2 structures, and then from half structures to full structures (simple to complex structures). Xiong et al. [15] found that NBD and TMD domain fusion events might have occurred during the above process and that these fusion events occurred at least four times during the transformation from the half-structured transporters to full structures. Some ABC proteins have lost their TMD, which leads to changes in their basic functions (e.g., ABCE and ABCF). During the evolution of the seven full-structure ABC transporters, ABCA, ABCB, ABCC and ABCG originated before the last eukaryotic common ancestor (LECA), whereas the ABCD, ABCE and ABCF families originated before terrestrial plants, archaea, and the differentiation between bacteria and archaea, respectively.

A large number of gene family duplications have occurred via whole-genome duplication (WGD) events [16–18]. Seret et al. [19] found that members of the ATP-binding cassette superfamily, namely, *Pdr5p* and *Snq2p*, derived from a common ancestor gene before WGD. In contrast, both *Pdr10p* (*Pdr5p* paralog) and *YNR070wp* (*Snq2p* paralog) originated from independent duplicating events after WGD. Duplication events of ABC transporter genes have occurred in both fish and mammalian genomes, but ABC transporter gene loss events have also occurred due to duplicating events in a large number of genes. Annilo et al. [20] found that both gene transformation and coevolution occurred during the introduction and loss of ABC transporter genes. Moreover, human ABC transporter genes show 94, 85 and 77% homology to those in mammals, chickens and zebrafish, respectively. However, only 41 ABC transporter genes can be found in chickens, which is fewest number of those found in any higher vertebrate, and no specific genome-duplication events have been detected in birds.

The *ABCG2* gene is located in a quantitative trait locus (QTL) in some livestock species, and mutations in *ABCG2* are associated with performance and disease traits in livestock and humans [21]. For example, *ABCG2* variants are likely to affect the milk yield and composition in Holstein cattle [21–23] and the development of gout [24] and drug resistance in human cancer cells [1, 25–28] (e.g., breast [1, 27], colon [25] and liver [28] cancer cells). However, the evolutionary process and functions of the *ABCG2-like* gene (*ABCG2* paralog and a member of the *ABCG2* gene subfamily in birds) remain unknown.

Knowledge of the molecular evolution of gene families is an important prerequisite for understanding the functional differences among protein family members and predicting new functions for their paralogous and orthologous genes. Therefore, this study aimed to analyze the genetic structure, genome duplication characteristics, chromosome distribution, phylogeny and other aspects of the bird *ABCG2* and *ABCG2-like* genes and to therefore determine the potential changes in and connections between these genes and their functions in birds. Overall, the present study can provide theoretical references for studying not only the trait regulatory functions of *ABCG2* and *ABCG2-like* genes but also the evolution aspects of the ABC transporter superfamily in birds.

## Results

### *ABCG2* gene subfamilies in different birds

Through BLAST, 77 *ABCG2* and *ABCG2-like* nucleic acid sequences, including 41 of the *ABCG2* gene and 36 of the *ABCG2-like* gene, were obtained from the genomes of 41 bird belonging to 33 families. Combined with the current NCBI nomenclature system, the BLAST results suggested that only two *ABCG2* gene subfamily members, i.e., the *ABCG2* and *ABCG2-like* genes, exist in birds. However, the *ABCG2-like* genes were lost in *Coturnix japonica* (Phasianidae), *Meleagris gallopavo* (Phasianidae), *Gallus gallus* (Phasianidae), *Mesitornis unicolor* (Mesitornithidae) and *Manacus vitellinus* (Pipridae) (Additional file 1, Tables S1 and S2).

### Phylogenetic analysis

**Fig. 1:**
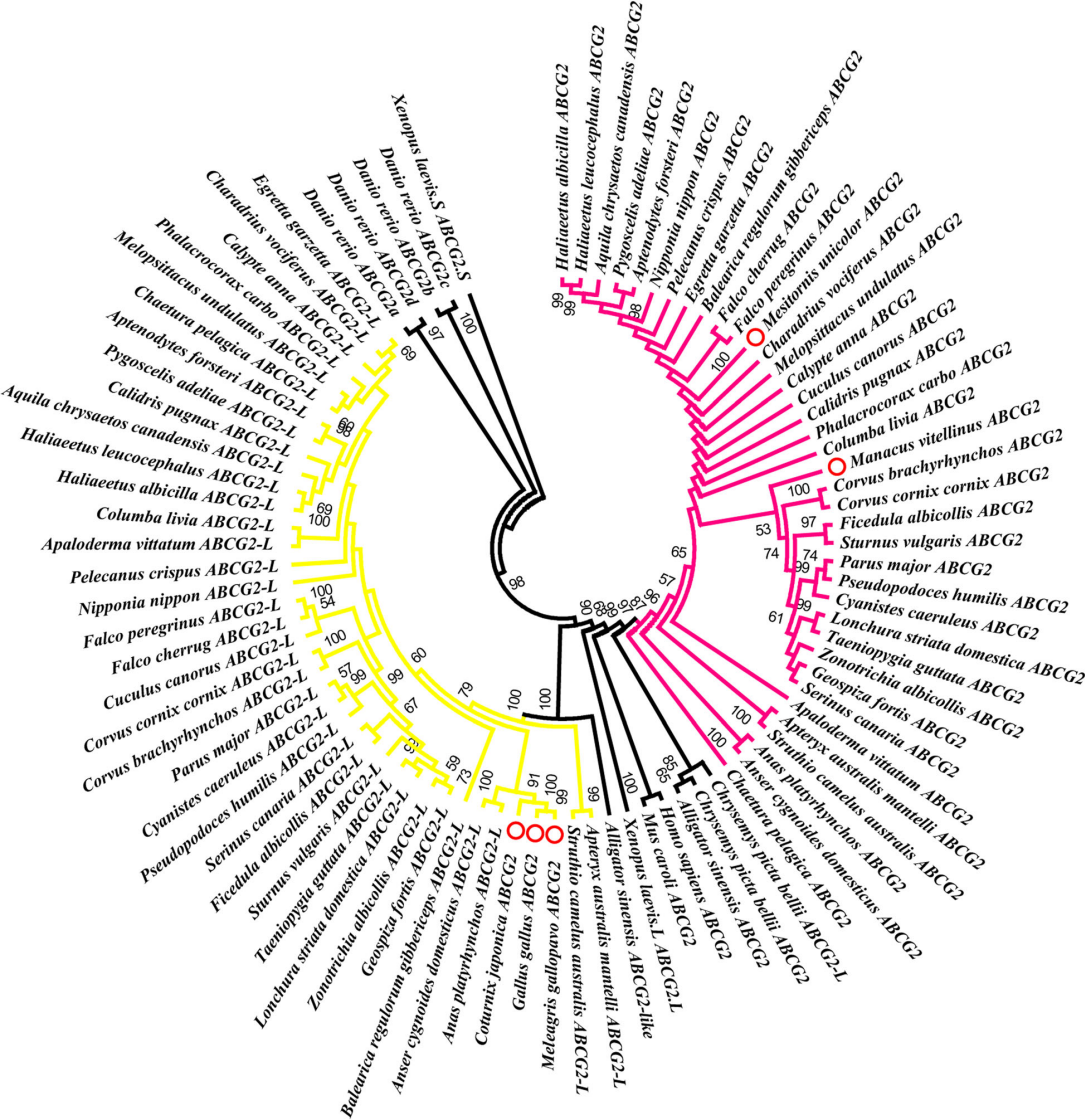
Evolutionary relationships of the *ABCG2* and *ABCG2-like* genes in birds. The phylogenetic tree was constructed with the ML method using MEGA7 software. The numbers on the nodes represent the bootstrap support. The outermost ring contains the Latin name and gene name of the birds and outgroup species, and *ABCG2-like* is abbreviated as *ABCG2-L*. The red circles indicate the birds whose *ABCG2-like* genes were lost

A maximum likelihood (ML) phylogenetic analysis (Fig. 1) was performed using the nucleic acid sequences (coding sequences) of the *ABCG2* and *ABCG2-like* genes, which were obtained from all birds (Additional file 1, Table S1) and outgroup species (Additional file 2, Table S3). The results supported the classification of the two gene subfamily members (*ABCG2* and *ABCG2-like*) in the 41 birds. However, phylogenetic trees also clustered the ABCG2 genes of 3 birds (*Coturnix japonica*, *Meleagris gallopavo* and *Gallus gallus*) were clustered in the clades of *ABCG2-like* genes (species marked with red circles in Fig. 1), suggesting that these three *ABCG2* genes and *ABCG2-like* genes have high homology. Although the clade nodes between the two subfamilies and between different species did not always exhibit sufficient phylogenetic resolution (the bootstrap support for some nodes was less than 50%), we could still deduce that the *ABCG2* and *ABCG2-like* genes of birds likely originated from a common ancestor. We subsequently consulted the comprehensive bird phylogeny described by Prum et al. [29] and found that the phylogenetic relationships among *ABCG2-like* genes were closer to the comprehensive phylogenetic relationship among birds than those among *ABCG2* genes. Therefore, we speculated that the *ABCG2-like* genes were more conserved than the *ABCG2* genes during the evolution of birds.

### Selection pressure analysis

**Fig. 2:**
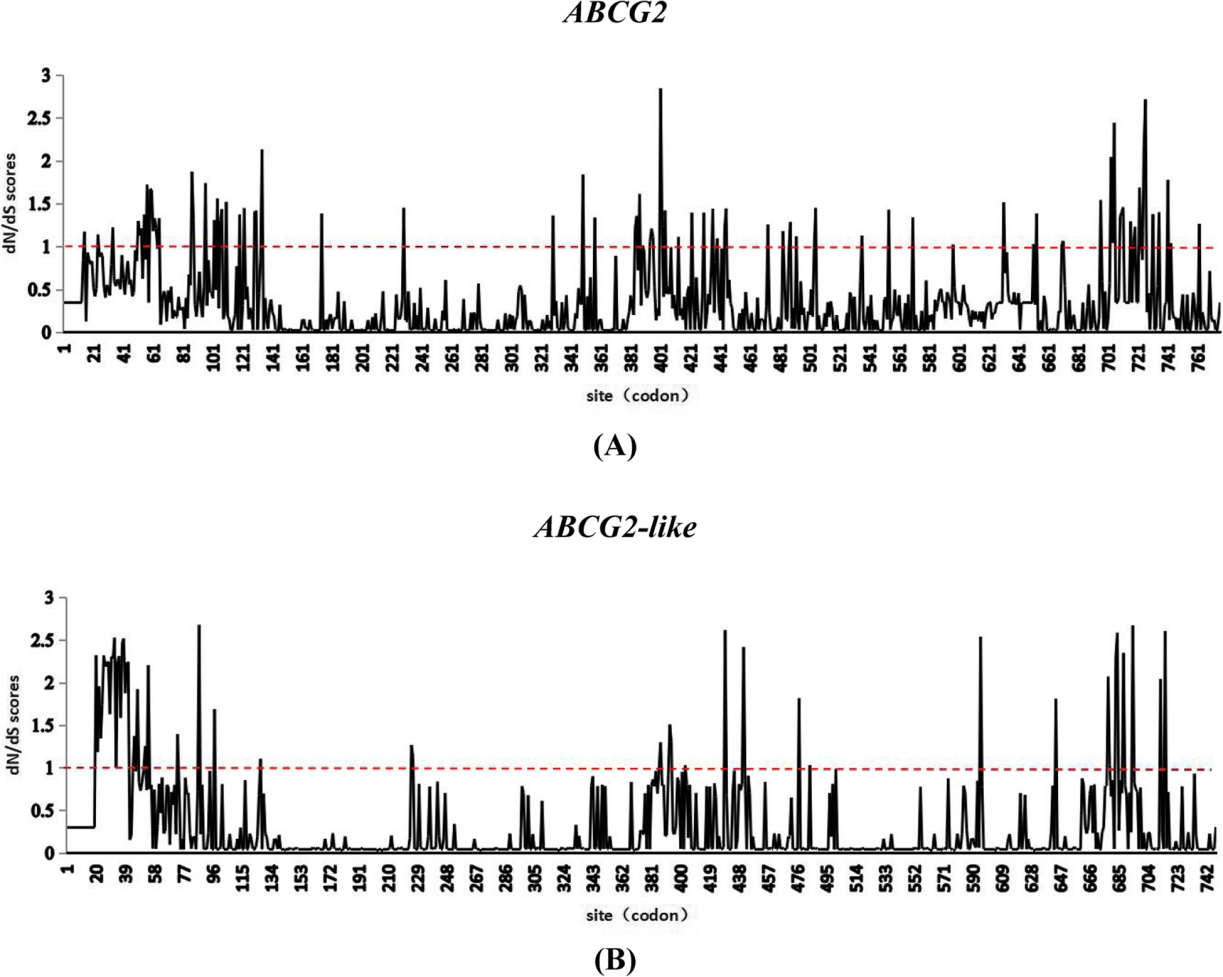
Sliding window plot of the dN/dS ratios obtained for the (**a**) *ABCG2* and (**b**) *ABCG2-like* genes. The value corresponding to neutral selection (dN/dS = 1) is indicated by the red dotted line

Using the CodeML program, positive and purifying selection analyses of the *ABCG2* and *ABCG2-like* gene sequences were performed for the various birds, as shown in Tables 1 and 2. In the M0 (one ratio) model, the ω values of *ABCG2* and *ABCG2-like* genes were 0.25797 and 0.23234, respectively (Tables 1 and 2), and these values were far less than 1. Therefore, the M0 model provides no direct evidence that the *ABCG2* and *ABCG2-like* genes were affected by positive selection pressure. Positive selection analysis was used to identify positive selection sites (Fig. 2) [30].

**Table 1:** Selection pressure analysis of sites in the coding region of *ABCG2* genes

Model code	lnL	Parameters	Number of positive selection sites
M0 (one ratio)	−19,249.376879	ω = 0.25797	None
M1a (nearly neutral)	−18,586.64149	P0 = 0.70173,P1 = 0.29827 ω0 = 0.07200, ω1 = 1	Not allowed
M2a (positive selection)	−18,561.41171	P0 = 0.69472,P1 = 0.27126,P2 = 0.03402 ω0 = 0.07356,ω1 = 1,ω2 = 2.61739	13
M3 (discrete)	−11,953.59808	P0 = 0.28795,P1 = 0.18745,P2 = 0.27619 ω0 = 0.03433,ω1 = 0.03433,ω2 = 0.03433	Not allowed
M7 (beta)	−18,532.88959	*p* = 0.24865,q = 0.61421(p1 = 0.1), ω = 0.00101	Not allowed
M8 (beta&ω > 1)	−18,489.18864	p0 = 0.9112692,*p* = 0.33179,q = 1.27694, (p1 = 0.08874) ω = 1.77811	56

Note: lnL refers to the logarithm of the maximum likelihood (ML)

**Table 2:** Selection pressure analysis of sites in the coding region of *ABCG2-like* genes

Model code	lnL	Parameters	Number of positive selection sites
M0 (one ratio)	−12,339.59447	ω = 0.23234	None
M1a (nearly neutral)	−11,969.60128	P0 = 0.77409,P1 = 0.22591 ω0 = 0.03929, ω1 = 1	Not allowed
M2a (positive selection)	−11,956.39673	P0 = 0.77460, P1 = 0.19600,P2 = 0.02940 ω0 = 0.04171, ω1 = 1, ω2 = 3.43990	16
M3 (discrete)	−18,486.07613	P0 = 0.30973,P1 = 0.31583,P2 = 0.24289,ω0 = 0,ω1 = 0.11046,ω2 = 0.44219	Not allowed
M7 (beta)	−11,978.85492	*p* = 0.10690,q = 0.34331 (p1 = 0.10000), ω = 0	Not allowed
M8 (beta&ω > 1)	−11,956.1596	p0 = 0.95146,*p* = 0.13558,q = 0.57516, (p1 = 0.04854) ω = 2.55211	36

Note: lnL refers to the logarithm of the maximum likelihood (ML)

A likelihood ratio test (LRT) was performed to compare the M1a (nearly neutral) and M2a (positive selection) models (Additional file 3, Tables S4 and S5). The statistical values (ΔlnL) of *ABCG2* and *ABCG2-like* genes were 25 (*p* < 0.01) and 13 (*p* < 0.01), respectively. Therefore, the M2a model was superior to the M1a model.

In addition, the LRT comparison of the M7 (beta) and M8 (beta and ω > 1) models revealed a significant difference between the models. The ΔlnL values of the *ABCG2* and *ABCG2-like* genes were 43 (*p* < 0.01) and 22 (*p* < 0.01), respectively (Additional file 3, Tables S4 and S5), and the M8 model was superior to the M7 model.

Based on the M2a model, a total of 13 positive selection sites (in codons) were found for the *ABCG2* genes (Additional file 4, Tables S6 and S7). Among these sites, three were statistically significant (*p* < 0.05), and two sites were extremely significant (*p* < 0.01). Moreover, a total of 16 positive selection sites were found for the *ABCG2-like* genes (Additional file 4, Tables S6 and S7), and only three of these were statistically significant (*p* < 0.05).

However, we found a total of 56 positive selection sites in the coding regions of the *ABCG2* genes (Additional file 4, Tables S6 and S7) with the M8 model. Six positive selection sites were statistically significant (*p* < 0.05), and eight positive selection sites were extremely significant (*p* < 0.01). Furthermore, a total of 36 positive selection sites were found for the *ABCG2-like* genes (Additional file 4, Tables S6 and Table S7). Among these, two were statistically significant positive selection sites (*p* < 0.05), and two were extremely significant positive selection sites (p < 0.01). Moreover, the analysis of the M3 model revealed that approximately 95.7 and 98.5% of the sites in the *ABCG2* and *ABCG2-like* gene sequences, respectively, were affected by negative selection.

These results showed that the *ABCG2* and *ABCG2-like* genes were mainly subject to strong purifying selection, but the *ABCG2* genes were affected by stronger positive selection pressure in birds compared with the *ABCG2-like* genes. Additionally, the *ABCG2-like* gene sequences were more conserved than the *ABCG2* gene sequences in birds.

### Chromosomal synteny analysis of *ABCG2* and *ABCG2-like* genes

**Fig. 3:**
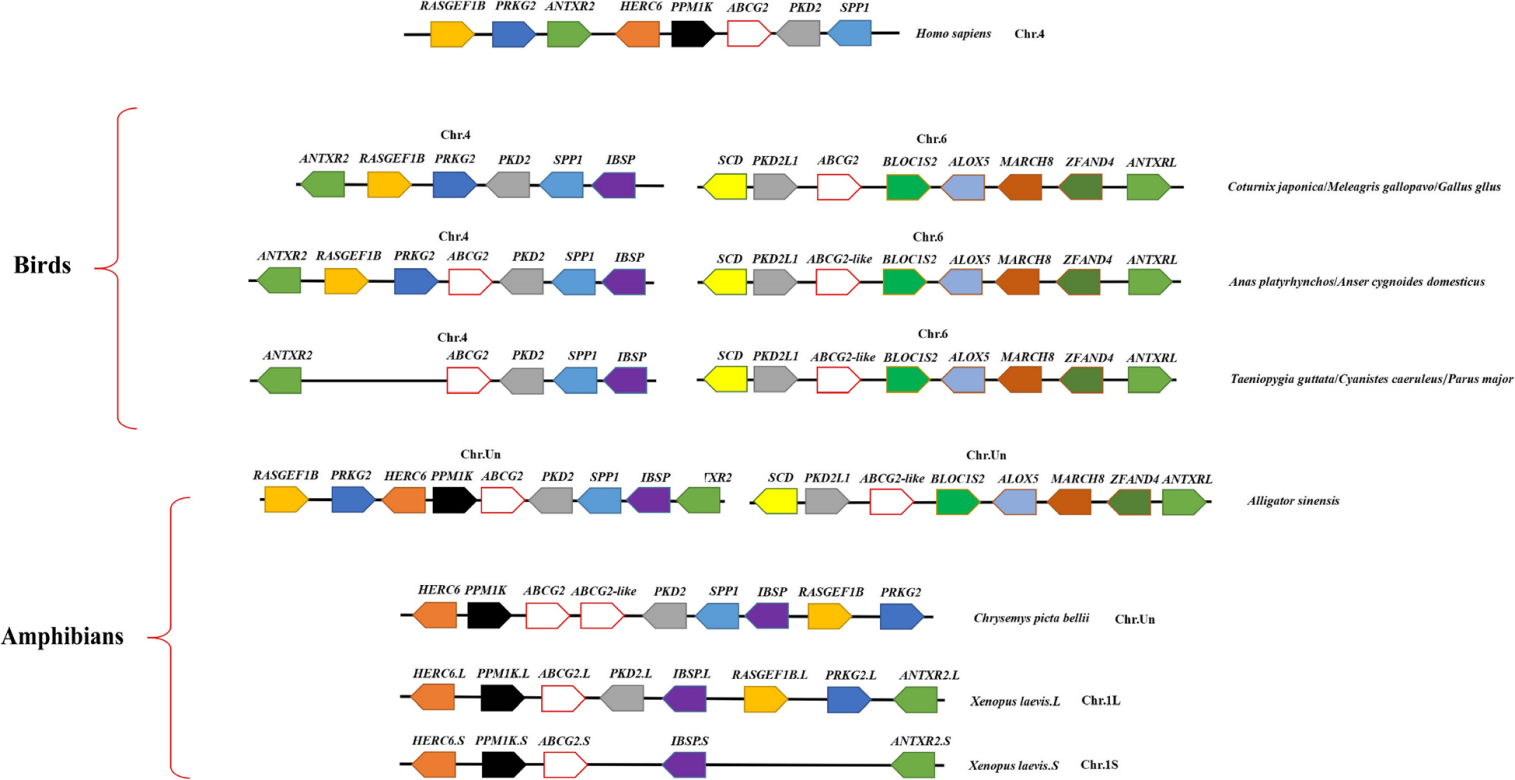
Genomically conserved synteny analysis of the *ABCG2* and *ABCG2-like* genes. Different genes are represented by colored pentagons. The gene names are shown on the top of the pentagons, and pentagons with the same color represent members of the same gene family. We arranged the order of the pentagons based on the relative positions of genes in the chromosome or scaffold

A chromosomal synteny analysis was performed with several representative birds using the Genome Data Viewer (GDV) from the NCBI and Ensembl 94. Conserved synteny dot plots showed that the neighborhood regions of the *ABCG2* and *ABCG2-like* genes in seven birds were similar to those in *Homo sapiens*, *Alligator sinensis*, *Chrysemys picta bellii* and *Xenopus laevis* (Fig. 3). Conserved chromosome segments (Fig. 3) were found in *Anas platyrhynchos* and *Taeniopygia guttata*, and the conserved chromosome segments are *ABCG2*-*PKD2*-*SPP1-IBSP* (chromosome 4) and *BLOC1S2*-*ABCG2-like*-*PKD2L1*-*SCD* (chromosome 6). Conserved chromosome segments (Fig. 3) were also found in *Gallus gallus, Meleagris gallopavo* and *Coturnix japonica* (deletion of the *ABCG2-like* gene occurred in the genomes of *Gallus gallus, Meleagris gallopavo* and *Coturnix japonica*), and these conserved chromosome segments are *PKD2*-*SPP1-IBSP* (chromosome 4) and *BLOC1S2*-*ABCG2*-*PKD2L1*-*SCD* (chromosome 6). These results reflected the overall evolutionary conservation of *ABCG2* and *ABCG2-like* genes in birds. However, deletion of the *ABCG2* gene occurred in some birds, which was contrary to the characteristics of genome conservation, and the complex reasons for this result would be worth further study. Moreover, the *ABCG2* and *ABCG2-like* genes were found adjacent to each other on the same chromosome in *Chrysemys picta bellii.* In addition, only a single copy of the *ABCG2* gene was found in the *Xenopus laevis* genome.

### Exon/intron structure and splicing site analysis

**Fig. 4:**
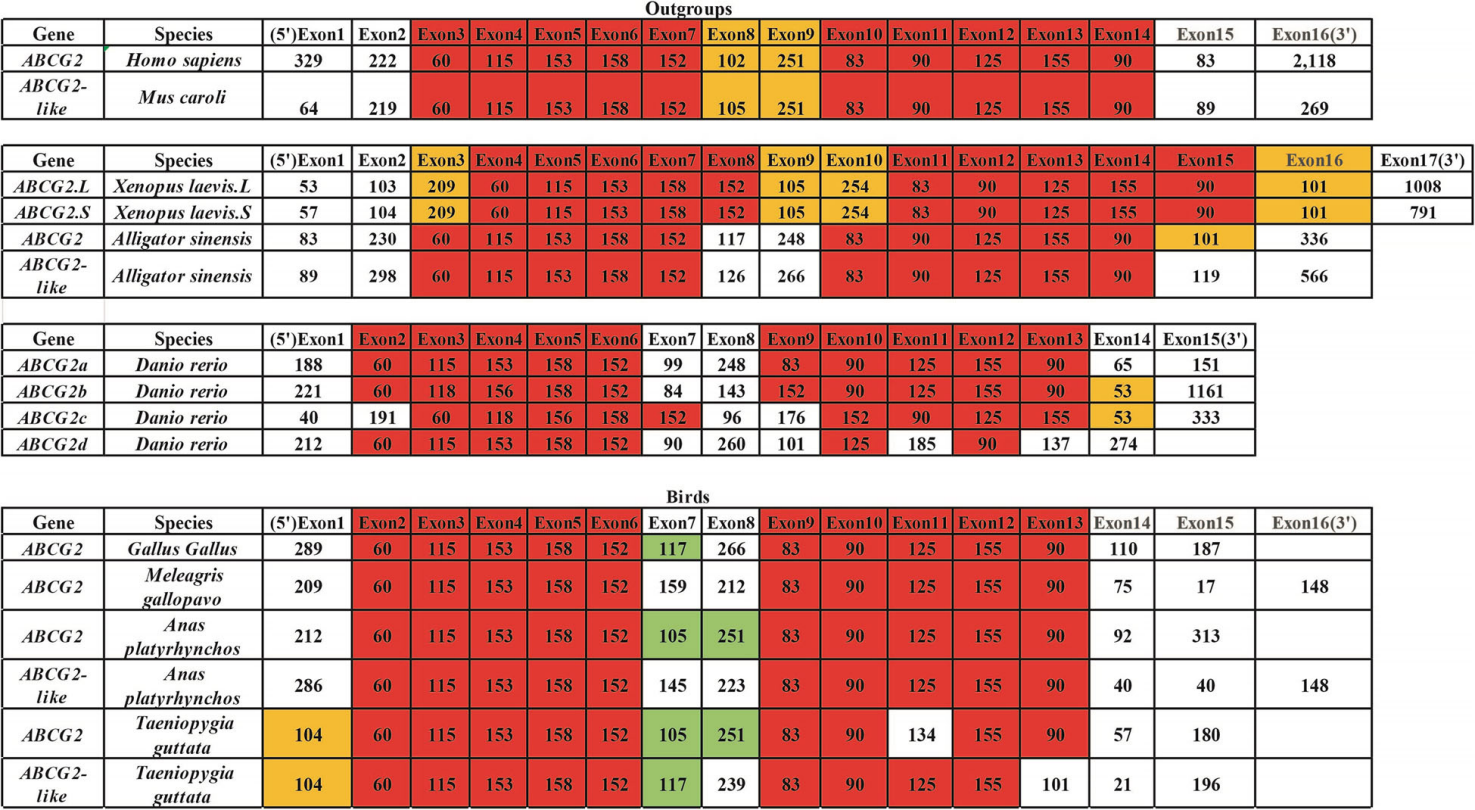
Exon structure (5′-3′) and splicing sites of the *ABCG2* and *ABCG2-like* genes in each birds and outgroup species in series or sections. The numbers in the boxes are the nucleotide lengths. The order of each exon is indicated in the first row. The red exon is conserved in all bird *ABCG2* and *ABCG2-like* genes. The size of each exon is not drawn to scale

**Fig. 5:**
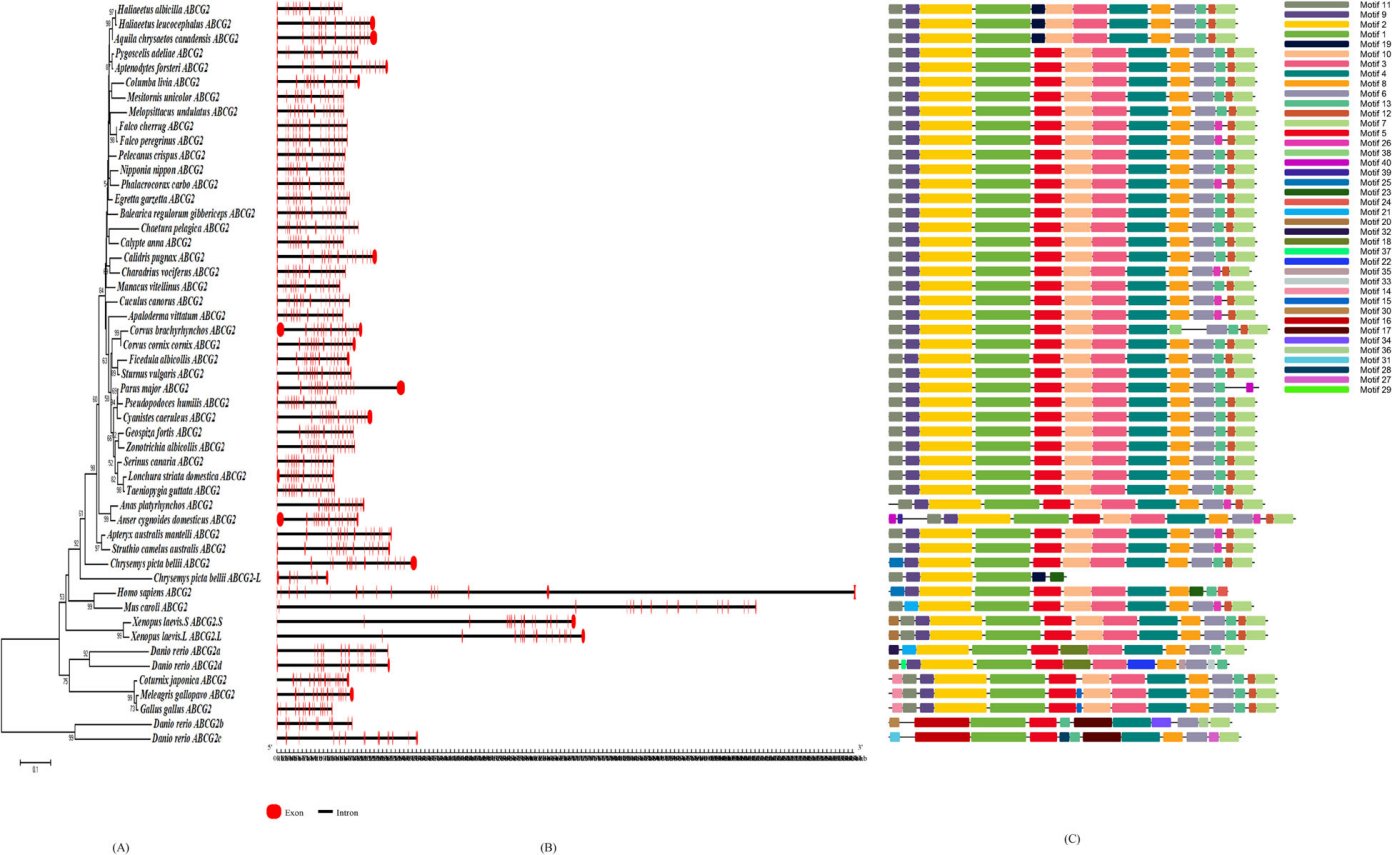
Phylogenetic analysis (**a**), gene exons/intron structure (**b**) and protein domain structures (**c**) of the *ABCG2* genes in birds. The phylogenetic tree of the amino acid sequences was constructed with the NJ method using MEGA7 software. The numbers on the nodes represent the bootstrap support. The red boxes and black lines (drawn to scale, as shown in the figure below) represent the exons and introns, respectively. The protein motifs are shown as colored boxes based on the MEME identification

In the current study, the exon/intron structures of each *ABCG2* or *ABCG2-like* gene sequence were obtained from the 41 birds and outgroup species (Figs. 4b and 5b). An exon/intron structure analysis showed that the CDSs were interrupted by several introns. Compared with *Homo sapiens* and *Mus caroli*, birds harbored significantly shorter *ABCG2* and *ABCG2-like* genes.

**Fig. 6:**
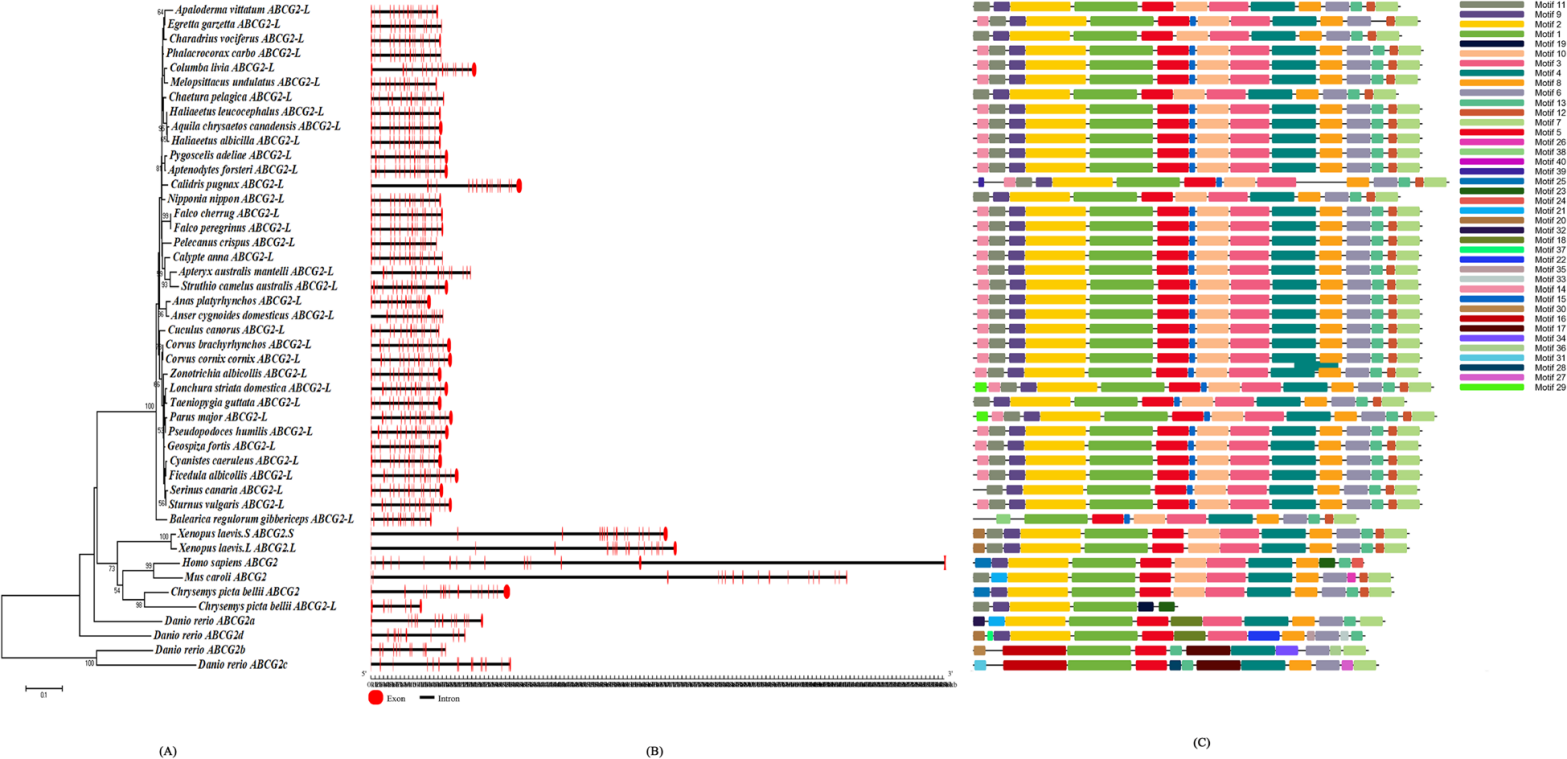
Phylogenetic analysis (**a**), gene exons/intron structure (**b**) structures and protein domain structures (**c**) of the *ABCG2-like* genes in birds. The phylogenetic tree of the amino acid sequences was constructed with the NJ method using MEGA7 software. The numbers on the nodes represent the bootstrap support. The red boxes and black lines (drawn to scale) represent the exons and introns, respectively. The protein motifs are shown as colored boxes based on the MEME identification

According to the structure analysis, 22 *ABCG2* gene sequences and 24 *ABCG2-like* gene sequences were composed of 15 exons, whereas 13 *ABCG2* gene sequences and 15 *ABCG2-like* gene sequences were composed of 16 exons. Only two *ABCG2-like* genes were composed of 17 exons, and one *ABCG2* gene was composed of 14 exons (Figs. 5b and 6b). Most members of each individual subfamily contained more than seven similar and conserved exons. The exons marked in red, as shown in Fig. 4, were conserved in length and consisted of 60, 115, 153, 158, 152, 83, 90, 125, 155, 156 and 90 nucleotides. However, the lengths of the first and last exons of the CDSs of the *ABCG2* or *ABCG2-like* genes varied significantly among birds (*p* < 0.05), and these exons were identified in the *ABCG2* or *ABCG2-like* gene sequences of all the studied birds, *Homo sapiens*, *Mus caroli* and *Danio rerio*. At least five conserved exons were copied in series or piecewise in the *ABCG2* and *ABCG2-like* genes, supporting the hypothesis of a common ancestral relationship among *ABCG2* and *ABCG-like* gene sequences (Fig. 4).

### Amino acid sequence domain analysis of ABCG2 and ABCG2-like proteins

The protein domains of the ABCG2 and ABCG2-like protein sequences from the 41 birds and outgroup species were predicted in this study (Figs. 5c and 6c). A total of 40 motifs, named 1–40, were identified in the full-length amino acid sequences of the ABCG2 and ABCG2-like proteins (Additional file 5).

Most amino acid sequences of the *ABCG2* gene subfamily shared more than 12 common motifs, which indicated that both ABCG2 and ABCG2-like proteins were highly conserved (Figs. 5c and 6c). However, some small differences were found in the N-terminal region. Most ABCG2-like amino acid sequences of birds harbored a specific motif (named 14 in Figs. 5c and 6c), whereas the ABCG2 amino acid sequences of birds did not have this motif in the N-terminal region.

In addition, the ABCG2 protein sequences of *Anas platyrhynchos* and *Anser cygnoides domesticus* contain seven and eight transmembrane helical structures, respectively, and the ABCG2 protein sequences of *Parus major* contain four transmembrane helical structures. The amino acid sequences of the *ABCG2* genes in other birds have five transmembrane helices, and those of *ABCG2-like* genes in all birds also have five transmembrane helices (Additional file 6). In summary, with the exception of the ABCG2 amino acid sequences of *Anas platyrhynchos*, *Anser cygnoides domesticus* and *Parus major*, the ABCG2 and ABCG2-like amino acid sequences of most birds have similar transmembrane structures. Moreover, these sequences have transmembrane structures similar to those of the ABCG2 and ABCG2-like proteins of some amphibians.

These results suggested that ABCG2 proteins are homologous to ABCG2-like proteins and that those in the same subgroup might have similar functions. However, functional differences between the two proteins cannot be excluded. Although the function of these conserved motifs has not been elucidated, some of the motifs might determine differences in the transport functions of the two proteins.

### Phosphorylation site analysis of *ABCG2* and ABCG*2-like* genes

The potential serine (S), threonine (T), and tyrosine (Y) phosphorylation sites (Additional file 7, Table S8) in the ABCG2 and ABCG2-like protein sequences of the 41 birds were predicted. Approximately 11, 6 and 17% of the S, T and Y residues, respectively, in the ABCG2 amino acid sequences were predicted as phosphorylated sites, and approximately 11, 11 and 13% of the S, T and Y residues, respectively, in the ABCG2-like protein sequences were predicted as phosphorylated sites.

The number of S phosphorylation sites in the ABCG2 protein varied greatly among the different studied birds. Similar results were also obtained for the ABCG2-like protein; however, compared with the number of S sites in ABCG2 proteins, that in the ABCG2-like proteins was less variable across the birds. According to the results (Additional file 8), all S residues were concentrated in the NBD region of the amino acid sequence, and a small number of phosphorylation sites were located in the TMD regions. Compared with the ABCG2 protein, the ABCG2-like protein contained more phosphorylation sites in the NBD region (Additional file 8). In terms of the total number of phosphorylation sites, we found that the TMD regions of the ABCG2 and ABCG2-like proteins were highly conserved. The differences in the number and distribution of phosphorylation sites between ABCG2 and ABCG2-like amino acid sequences might be related to their functions.

### Gene expression patterns in *Anas platyrhynchos* and conversion analysis

To establish the occurrence of gene conversion between paralogs, the nucleotide sequences of the *ABCG2* gene subfamily in some birds and outgroup species were analyzed (Additional file 1, Table S8) using GENECONV and SIMPLOT. The results from GENECONV showed no clear evidence of gene conversion events during the evolutionary process of birds, and no significant evidence demonstrates genetic conversion between the *ABCG2* gene of *Gallus gallus* and the *ABCG2-like* gene of *Anas platyrhynchos*. SIMPLOT also showed high shared sequence identity between the paralogs in the birds and outgroup species, and the results provided no clear evidence of gene conversion (Additional file 9).

**Fig. 7:**
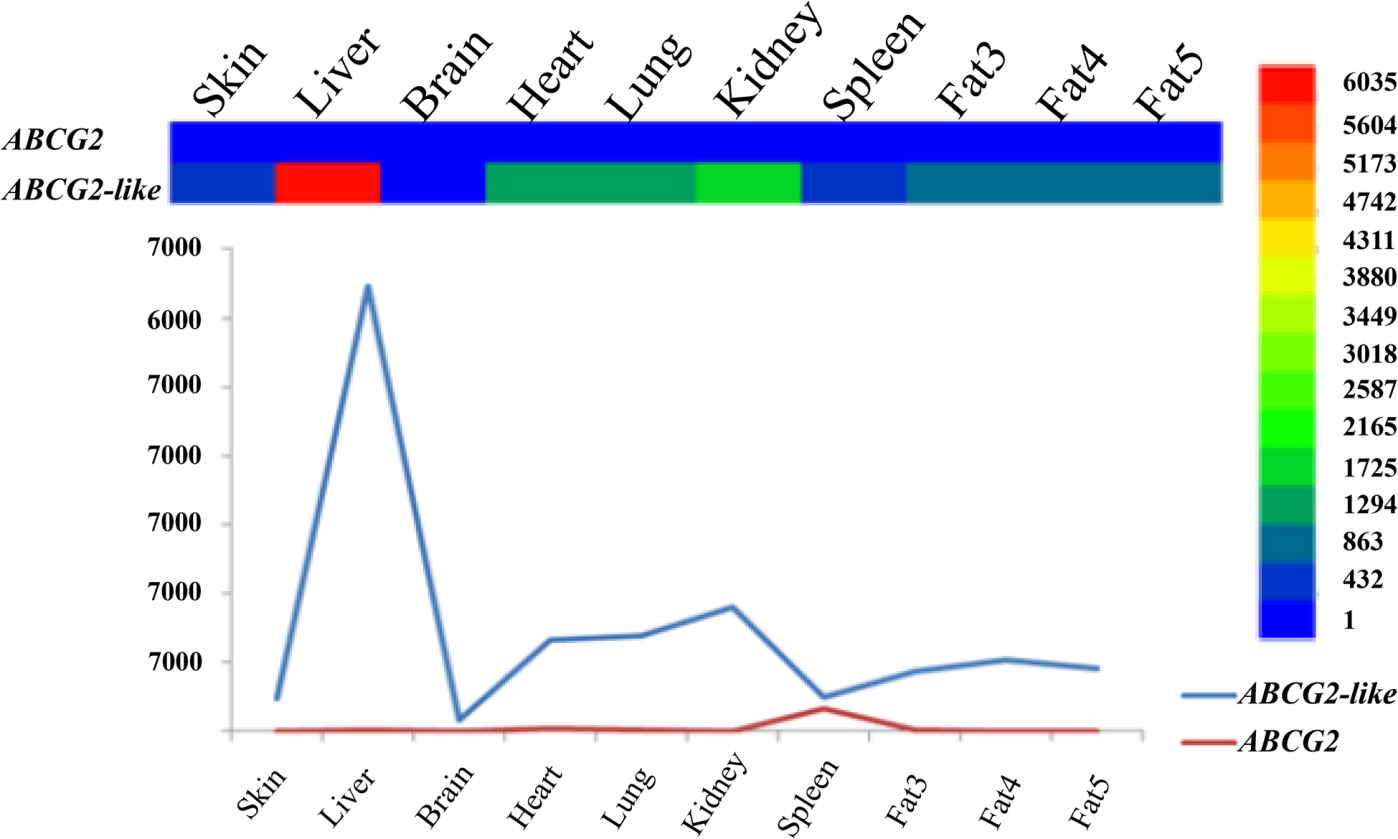
Gene expression patterns of the *ABCG2* and *ABCG2-like* genes in mallards

Data on the expression of *ABCG2* and *ABCG2-like* genes in mallards were obtained from duckbase (http://duckbase.org/rnaseqExpression; Additional file 10). The expression levels of the *ABCG2* and *ABCG2-like* genes were highest in the spleen and liver, respectively, whereas the *ABCG2* gene was barely expressed in some tissues in mallards (Fig. 7). The expression patterns of these two genes were significantly different in mallard.

## Discussion

As an important transmembrane transporter protein, Zhou et al. [31] found that increasing the expression of the ABCG2 transport protein during the process of red blood cell maturation can reduce the level of intracellular protoporphyrin IX. Although the *ABCG2* gene has been investigated in various studies, the evolutionary process of the *ABCG2* gene subfamily members and the functional differences in this gene among birds have never been studied. In the present study, phylogenetic methods and comparative genomics were used to investigate the molecular evolution characteristics of *ABCG2* and *ABCG2-like* genes in birds. After an extensive database statistical analysis, *ABCG2* subfamily genes were found to be widely present in chordates and vertebrates. Overall, *ABCG2* or *ABCG2-like* genes were found in 41 birds, and *ABCG2-like* genes were lost in only five birds.

### Origin, duplication events and conversion of the *ABCG2* and *ABCG2-like* genes in birds

The genomic structure of birds is relatively evolutionarily stable in evolution [32], and chromosomes 1–10 and Z are the ancestors of almost all chromosomes in birds [33]. Moreover, two rounds (2R) of genome duplication occurred during the early diversification of chordates and vertebrates [34, 35], which provides a theoretical basis for studying the evolution of the *ABCG2* and *ABCG2-like* genes in birds.

Based on a chromosomal synteny analysis (Fig. 3), *ABCG2* or *ABCG2-like* genes share similar gene neighborhoods in birds (including early birds), *Alligator sinensis* and *Xenopus laevis*. Furthermore, two members of the *ABCG2* gene subfamily were adjacent to each other in *Chrysemys picta bellii* and shared similar chromosomal neighborhoods with the *ABCG2* gene of birds. First, these results showed highly conserved chromosomal synteny in the neighborhood regions of *ABCG2* or *ABCG2-like* genes in birds. Moreover, these results confirmed that the characteristics of ancestral chromosomes were highly conserved and exhibit low segment recombination rates in birds [33, 36]. Although the NCBI database did not specify which chromosome in *Chrysemys picta bellii* harbored the *ABCG2* and *ABCG2-like* genes, Matusda Y et al. [37] found that chromosome 4 of birds is highly homologous with chromosome 4 of turtles (particularly between chickens and soft-shelled turtles). Chromosome 4 is very old in bird genomes and can be characterized by an early origin and strong evolutionary conservatism [38]. Based on the above-described results, we hypothesized that the *ABCG2* and *ABCG2-like* genes in *Chrysemys picta bellii* are also located on chromosome 4. We also assumed that the ancestors of the *ABCG2* gene subfamily members existed on chromosome 4 or the ancestor of chromosome 4 for a long time during the evolutionary process (Fig. 3), and this assumption is supported by the results from a phylogenetic analysis.

We also found that even more *ABCG2* gene subfamily members were located on the same chromosome in some fishes (Additional file 11). For example, the *ABCG2* gene and multiple *ABCG2-like* genes were located on chromosome 25 in *Astyanax mexicanus*, which suggested that multiple members of the *ABCG2* gene subfamily already existed in the same chromosome in early chordates. However, the evolutionary connection between the chromosome in fishes and that in amphibians remains unclear, and we can trace the origin of *ABCG2* and *ABCG2-like* genes back only to amphibians. We hypothesize that during the evolution of birds from fishes, most members (or multiple copies of a single member) of the *ABCG2* gene subfamily were lost, and ultimately, only two *ABCG2* gene subfamily members were retained in birds. Moreover, only one copy of the *ABCG2* gene in frog genomes is located on chromosome 1. These findings produce an unusual situation. The reasons for this phenomenon are very complicated, and no clear conclusion has been reached. Some studies have suggested that chromosome fusion occurred during the process of speciation in frogs [39], which might have caused the deletion of *ABCG2* gene subfamily members in frogs. These findings provides a reference for explaining the large number of deletions of *ABCG2* gene subfamily members during the evolution of birds from fishes.

Some of the *ABCG2-like* genes in birds are located on chromosome 6. *ABCG2-like* neighborhoods similar to those in birds have been found in some alligator genomes (Fig. 3). Based on the results from the phylogenetic and chromosomal synteny analyses, we hypothesized that the presence of *ABCG2* and *ABCG2-like* genes on different chromosomes could be traced back to either after the differentiation of turtle and birds or after the 2R WGD event.

We also found that one or more members of the *ABCG2* gene subfamily were also located on microchromosomes in some fishes, e.g., two *ABCG2-like* genes are located on the LG5 chromosome (Additional file 11). Some of these members have similar neighborhood segments among bird lineages and amphibians (Additional file 11). Therefore, we speculated that multiple microchromosomes containing *ABCG2-like* genes were further fused into a complete chromosome and that multiple *ABCG2-like* genes were lost during this process. Ultimately, only one *ABCG2-like* gene was retained in birds, and this gene did not originate from the WGD event. The timing of the appearance of chromosomes 6 to 9 in birds has not yet been determined [33]. Studying the origin of *ABCG2* and *ABCG2-like* genes can provide a reference for studying the origin of chromosome 6 in birds.

The current study provides no clear evidence of genetic conversion between *ABCG2* and *ABCG2-like* genes (Additional file 9), which supports the hypothesis of an independent origin of *ABCG2* and *ABCG2-like* genes. Olsen et al. [22] found that the *ABCG2*-*PKD2*-*SPP1* segment was located in a QTL. In the present study, the above segment was also found in some birds. Therefore, it can be speculated that the *ABCG2* gene controls similar quantitative traits in birds.

### Influence of positive and purifying selection on the *ABCG2* and *ABCG2-like* genes in birds

A selection pressure analysis of the *ABCG2* and *ABCG2-like* genes showed that both genes were more affected by purifying selection than by positive selection pressure. These results further supported the hypothesis that both *ABCG2* and *ABCG2-like* genes are highly conserved in birds. We also inferred that both *ABCG2* and *ABCG2-like* genes have some similar functions but exhibit some differences, e.g., regulation of some quantitative traits. The selection pressure sites were mostly concentrated at the N terminus (NBD region), which also indicated a difference in the N terminus between the two genes that might affect their function. Compared with the *ABCG2-like* genes, the *ABCG2* genes were under stronger positive selection pressure. Therefore, the *ABCG2* gene was more evolutionarily active. This finding motivates studies of *ABCG2* and *ABCG2-like* gene deletions in some birds.

### Gene and protein structures of the *ABCG2* and *ABCG2-like* genes in birds and functional differences between these genes during evolution

An exon/intron structure analysis can provide valuable information on duplication events within gene families that occurred during eukaryotic evolution, and the gain and loss of introns reflect positive or negative correlations with the CDS evolutionary rate [40]. In addition, an exon/intron structure analysis provides a theoretical reference for exploring the functional differences in gene families. Based on the gene structure analysis, all *ABCG2* or *ABCG2-like* genes, with the exception of the *Balearica regulorum gibbericeps ABCG2* gene, contained more than 15 exons in their CDSs (Figs. 5 and 6). A sequence length of more than seven exons was conserved in birds (Fig. 7). However, birds contained much shorter introns than other outgroup species (Figs. 5 and 6), which suggests that the *ABCG2* and *ABCG2-like* genes have similar functions. We thus conclude that the *ABCG2* and *ABCG2-like* genes perform unique functions in birds.

Through an analysis of the ABCG2 and ABCG2-like protein domains, one additional motif was found in the N terminus of *ABCG2-like* (Figs. 5 and 6; Additional file 5), and this motif could be responsible for the functional differences between the two genes. However, more than 12 similar motifs were found to exist in the whole proteins of various birds (Figs. 5 and 6; Additional file 5), supporting the notion of strong conservation and similarity among ABCG2 and ABCG2-like proteins, and the analysis also revealed some inaccurate gene annotations. A detailed analysis clarified some of the inaccurate annotations, such as *ABCG2* of *Gallus gallus*, *Meleagris gallopavo*, and *Coturnix japonica*, which should be *ABCG2-like* genes based on a phylogenetic analysis of the three species in the same clade. The above gene and protein structural analyses provide evidence for the origin of *ABCG2* and *ABCG2-like* genes from 2R WGD.

The posttranslational modification of proteins can affect the biochemical properties of proteins and plays an important role in maintaining biological processes in cells. Protein phosphorylation can change the structure and functions of a protein [41]. A protein can have one or more phosphorylation sites, which make the protein structurally diverse. A phosphorylation site analysis of the amino acid sequences based on the *ABCG2* and *ABCG2-like* genes (Additional files 7 and 8) suggested that the phosphorylation sites were also concentrated in the ATP-binding domain (N terminus) of ABCG2 and ABCG2-like proteins, but there were also certain differences. The number of S and T sites in the *ABCG2* gene was significantly larger than that in the *ABCG2-like* gene, which indicated that the structural differences between *ABCG2* and *ABCG2-like* genes at the N terminus might affect the actual functions of these genes in birds.

### Expression and function of *ABCG2* and *ABCG2-lik*e genes

Studying the expression of gene families in animal tissues can provide a reference for exploring the functional differences between gene families. The expression level of the *ABCG2-like* gene was significantly higher than that of the *ABCG2* gene in most bird tissues (e.g., liver, heart, lung, and kidney; Fig. 7). The *ABCG2* gene was almost not expressed in some tissues. We speculated that the *ABCG2-like* gene might play a more important role than the *ABCG2* gene or functionally replace this gene in some tissues of mallards. Alternatively, the *ABCG2-like* gene replaced the function of the *ABCG2* gene in some tissues of mallard. Even though some genomes of birds have lost the *ABCG2* and *ABCG2-like* genes, we speculated that ABCG2 might be functionally redundant in some birds and that its deletion can thus be tolerated. The expression patterns of *ABCG2* and *ABCG2-like* genes in birds require further research. Overall, the results of the present study provide new ideas for future studies on the deletion of *ABCG2* gene subfamily members throughout the evolutionary process of vertebrates and chordates and on the deletion of *ABCG2* or *ABCG2-like* genes in some birds.

## Conclusion

The diversity of the *ABCG2* gene subfamily members has declined in birds, and most birds have only retained *ABCG2* and *ABCG2-like* genes. Here, we speculated that the *ABCG2* and *ABCG2-like* genes might have originated from the same ancestral chromosome and that these two genes might have been produced via genome duplication events during the evolution of amphibians to birds. The protein sequences of the *ABCG2* and *ABCG2-like* genes were structurally conserved and homologous, but these sequences were less conserved at the N terminus. These results indicate that the functions of *ABCG2* and *ABCG2-like* genes are generally similar; however, differences in the N-terminal structure might have led to the functional differences between the two genes in some birds.

## Methods

### Acquisition and identification of the *ABCG2* and *ABCG2-like* genes in birds

The nucleic acid coding sequences and amino acid sequences used in this study were obtained from NCBI (https://www.ncbi.nlm.nih.gov/). We used the ABCG2 amino acid sequence of *Anas platyrhynchos* in a BLASTP search and obtained 41 ABCG2 amino acid sequences and 36 ABCG2-like amino acid sequences from 41 representative birds. All obtained sequences had E-scores less than 0.01. The corresponding nucleic acid sequences were obtained using tblastn [42]. The amino acid sequences identified by BLAST were used in a BLASTX search to ensure that the nucleic acid and amino acid sequences of each *ABCG2* and *ABCG2-like* gene and protein matched each other [43]. We completed all of the sequence searches in August 2018.

### Phylogenetic analysis

Nucleic acid sequences (CDSs) were used for the phylogenetic analysis of *ABCG2* and *ABCG2-like* genes in birds. A nucleic acid sequence alignment was performed using Clustal Omega included in MEGA7 (Additional file 12). Revised sequence alignments were then submitted to MEGA7 to select the appropriate DNA evolution model according to our dataset [44]. Here, we found that the nucleic acid sequence group of the *ABCG2* gene subfamily followed a K2 + G + I model.

MEGA7 software was used to construct a bootstrap (1000 replicate) tree [45] of the nucleic acid sequences, and the ML method was used in the phylogenetic analysis. The ML search was started with the tree generated using BIONJ [46], and the optimal tree was determined through a heuristic search using the nearest-neighbor interchange (NNI) algorithm [43].

### Selective pressure analysis

In the selective pressure analysis, we obtained values of omega (nonsynonymous/synonymous replacement rate ratio, dN/dS) to analyze the evolutionary selection pressure at the molecular level. The variable omega (ω) intuitively reflects the evolutionary trend of organisms at the codon level, and omega> 1, omega = 1 and omega< 1 represent genes subjected to positive selection, neutral selection and negative selection (purification selection) during evolution, respectively [47].

The ω values were calculated using the CodeML program in the PAML 4.9 package [48]. We selected the M0, M1a, M2a, M3, M7 and M8 models (site models) [49, 50] for the following reasons: (1) the M0 model allows uniform selection pressure between different sites in a sequence, and the M1a, M7, and M8 models do not allow sites with ω > 1; (2) the M3 model assumes variable selection pressure between sites; and (3) the M2a and M8 models allow sites with ω > 1 [51]. With LRTs, we compared three groups (M1a versus M2, M0 versus M3 [52–55], and M7 versus M8 [51]) to infer the most suitable model.

We then used M2a and M8 to identify the positive selection sites. The Bayes empirical Bayes (BEB) calculation method was adopted to identify the positive selection sites, and the posterior probability (PP) of these sites was analyzed [56]. We considered only positive sites with a PP > 95%.

### Sequence analysis

First, we used GSDS online analysis software (http://gsds.cbi.pku.edu.cn/) [57] to analyze the exon/intron structure and exon distribution patterns of *ABCG2* and *ABCG2-like* genes. To study the conserved motifs of ABCG2 and ABCG2-like proteins, MEME online analysis software (http://meme.nbcr.net/meme/intro.html) was used [58] to predict protein structural domains. The optimized parameters of MEME were as follows: maximum number of motifs, 40 [59]; optimal motif width, 10–100 residues [59], and optimal width of each motif, 10–100 residues [59]. TMHMM software (http://www.cbs.dtu.dk/services/TMHMM/) was used to predict the transmembrane structure domain of the ABCG2 and ABCG2-like amino acid sequences [60]. The ABCG2 and ABCG2-like protein sequence phosphorylation sites were predicted using the online program KinasePhos 2.0 (http://kinasephos2.mbc.nctu.edu.tw/) [61] with the default parameters. The aligned sequences were then examined for possible gene conversion events by constructing a sliding window genetic diversity plot (SIMPLOT 3.5.1) [62]. GENECONV 1.8 [63] software was used for conversion analysis using the default parameters and a global segment *p* value (*p* < 0.05) corrected with 10,000 pseudoreplicates.

## Supplementary information

**Additional file 1: Table S1.** GenBank numbers of the bird ABCG2 and ABCG2-like amino acid sequences obtained in our study. **Table S2** GenBank numbers of the bird *ABCG2* and *ABCG2-like* nucleic acid sequences obtained in our study.

**Additional file 2: Table S3.** Nucleic acid sequences GenBank numbers of the *ABCG2* gene subfamily members in outgroup species used in the phylogenetic analysis. **Table S8** Nucleic acid sequences GenBank numbers of the outgroup *ABCG2* gene subfamily member nucleic acid sequences used in the conversion analysis.

**Additional file 3: Table S4.** Likelihood ratio test statistics for evaluation of model fit in *ABCG2* gene. **Table S5** Likelihood ratio test statistics for evaluation of model fit in *ABCG2-like* gene.

**Additional file 4: Table S6.** Positive selection sites of *ABCG2* gene. **Table S7** Positive selection sites of *ABCG2-like* gene.

**Additional file 5.** Consensus sequences of the group specific motifs.

**Additional file 6. **Prediction results of the protein transmembrane structure of *ABCG2* gene subfamily members.

**Additional file 7: Table S7.** Number of phosphorylation sites in the ABCG2 and ABCG2-like amino acid sequences of birds.

**Additional file 8.** Information on the specific phosphorylation sites in the ABCG2 and ABCG2-like amino acid sequences in birds.

**Additional file 9. **Sequence similarity plots of coding and pseudogene sequences of the *ABCG2* and *ABCG2-like* gene families in birds and mammals.

**Additional file 10. **Expression of the *ABCG2* and *ABCG2-like* genes in mallards (*Anas platyrhynchos*).

**Additional file 11. **Chromosomal location of *ABCG2* gene subfamily members in some fishes.

**Additional file 12. **Multiple sequence alignments of the *ABCG2* and *ABCG2-like* genes with full-length amino acid sequences.

## Data Availability

All data and materials are shown within the manuscript or additional files. These data and materials are fully available without restriction.

## Abbreviations

ABC: ATP-binding cassette
ABCG2: ATP-binding cassette subfamily G member 2
ABCG2-L: ATP-binding cassette subfamily G member 2-like
BCRP: breast cancer resistance protein
BEB: Bayes empirical Bayes
CDS: coding sequence
dN: nonsynonymous
dS: synonymous
GDV: Genome Data Viewer
LECA: last eukaryotic common ancestor
LRT: likelihood ratio test
ML: maximum likelihood
NBD: nucleotide-binding domain
NCBI: National Center for Biotechnology Information
NNI: nearest-neighbor interchange
QTL: quantitative trait locus
PP: posterior probability
S: serine
T: threonine
TMD: transmembrane domain
WGD: whole-genome duplication
Y: tyrosine
2R: two rounds
ω: omega
