# Supplementary material for: Molecular evolution of the ATP-binding cassette subfamily G member 2 gene subfamily and its paralogs in birds
Source: BMC Evol Biol. 2020 Jul 14;20:85. doi: 10.1186/s12862-020-01654-z (PMC7362505; doi:10.1186/s12862-020-01654-z)
Supplement: Supplementary file 1 — Additional file 1: Table S1. GenBank numbers of the bird ABCG2 and ABCG2-like amino acid sequences obtained in our study. Table S2 GenBank numbers of the bird ABCG2 and ABCG2-like nucleic acid sequences obtained in our study. [file 12862_2020_1654_MOESM1_ESM.docx]

**Table S1 GenBank numbers of the bird ABCG2 and ABCG2-like amino acid sequences obtained in our study.**

| **Avian species** | **Gene** | **Genebank** | **Gene** | **Genebank** |
| --- | --- | --- | --- | --- |
| ***Aquila chrysaetos canadensis*** | ***ABCG2*** | **XP_011599112.1** | ***ABCG2-like*** | **XP_011595184.1** |
| ***Haliaeetus albicilla*** | ***ABCG2*** | **XP_009918793.1** | ***ABCG2-like*** | **XP_009910398.1** |
| ***Haliaeetus leucocephalus*** | ***ABCG2*** | **XP_010568154.1** | ***ABCG2-like*** | **XP_010561818.1** |
| ***Anas platyrhynchos*** | ***ABCG2*** | **XP_012948706.1** | ***ABCG2-like*** | **XP_005025255.1** |
| ***Anser cygnoides domesticus*** | ***ABCG2*** | **XP_013046088.1** | ***ABCG2-like*** | **XP_013056835.1** |
| ***Apaloderma vittatum*** | ***ABCG2*** | **XP_009865267.1** | ***ABCG2-like*** | **XP_009874487.1** |
| ***Aptenodytes forsteri*** | ***ABCG2*** | **XP_019327878.1** | ***ABCG2-like*** | **XP_009275988.1** |
| ***Apteryx australis mantelli*** | ***ABCG2*** | **XP_013812829.1** | ***ABCG2-like*** | **XP_013814295.1** |
| ***Balearica regulorum gibbericeps*** | ***ABCG2*** | **XP_010303776.1** | ***ABCG2-like*** | **XP_010305875.1** |
| ***Calidris pugnax*** | ***ABCG2*** | **XP_014816981.1** | ***ABCG2-like*** | **XP_014811848.1** |
| ***Calypte anna*** | ***ABCG2*** | **XP_008499029.1** | ***ABCG2-like*** | **XP_008500271.1** |
| ***Chaetura pelagica*** | ***ABCG2*** | **XP_010000049.1** | ***ABCG2-like*** | **XP_009999101.1** |
| ***Charadrius vociferus*** | ***ABCG2*** | **XP_009889049.1** | ***ABCG2-like*** | **XP_009887039.1** |
| ***Columba livia*** | ***ABCG2*** | **XP_021144731.1** | ***ABCG2-like*** | **XP_021149521.1** |
| ***Corvus brachyrhynchos*** | ***ABCG2*** | **XP_017599595.1** | ***ABCG2-like*** | **XP_017581441.1** |
| ***Corvus cornix cornix*** | ***ABCG2*** | **XP_019148760.1** | ***ABCG2-like*** | **XP_010409748.1** |
| ***Cuculus canorus*** | ***ABCG2*** | **XP_009557822.1** | ***ABCG2-like*** | **XP_009568999.1** |
| ***Cyanistes caeruleus*** | ***ABCG2*** | **XP_023782111.1** | ***ABCG2-like*** | **XP_023785307.1** |
| ***Egretta garzetta*** | ***ABCG2*** | **XP_009638289.1** | ***ABCG2-like*** | **XP_009640330.1** |
| ***Lonchura striata domestica*** | ***ABCG2*** | **XP_021382150.1** | ***ABCG2-like*** | **XP_021394548.1** |
| ***Taeniopygia guttata*** | ***ABCG2*** | **XP_002190340.2** | ***ABCG2-like*** | **XP_012429985.1** |
| ***Falco cherrug*** | ***ABCG2*** | **XP_014139881.1** | ***ABCG2-like*** | **XP_005441670.1** |
| ***Falco peregrinus*** | ***ABCG2*** | **XP_013151588.1** | ***ABCG2-like*** | **XP_005234646.1** |
| ***Ficedula albicollis*** | ***ABCG2*** | **XP_005044772.1** | ***ABCG2-like*** | **XP_016155053.1** |
| ***Serinus canaria*** | ***ABCG2*** | **XP_009099622.1** | ***ABCG2-like*** | **XP_009085605.1** |
| ***Geospiza fortis*** | ***ABCG2*** | **XP_014167420.1** | ***ABCG2-like*** | **XP_005416554.1** |
| ***Manacus vitellinus*** | ***ABCG2*** | **XP_017940081.1** | ***ABCG2-like*** | **lost** |
| ***Melopsittacus undulatus*** | ***ABCG2*** | **XP_005155284.1** | ***ABCG2-like*** | **XP_005153946.1** |
| ***Mesitornis unicolor*** | ***ABCG2*** | **XP_010184629.1** | ***ABCG2-like*** | **lost** |
| ***Nipponia nippon*** | ***ABCG2*** | **XP_009465909.1** | ***ABCG2-like*** | **XP_009464204.1** |
| ***Parus major*** | ***ABCG2*** | **XP_015480451.1** | ***ABCG2-like*** | **XP_015488431.1** |
| ***Pelecanus crispus*** | ***ABCG2*** | **XP_009489087.1** | ***ABCG2-like*** | **XP_009489087.1** |
| ***Phalacrocorax carbo*** | ***ABCG2*** | **XP_009510213.1** | ***ABCG2-like*** | **XP_009507174.1** |
| ***Meleagris gallopavo*** | ***ABCG2*** | **XP_010712746.1** | ***ABCG2-like*** | **Lost** |
| ***Gallus gallus*** | ***ABCG2*** | **NP_001315419.1** | ***ABCG2-like*** | **lost** |
| ***Coturnix japonica*** | ***ABCG2*** | **XP_015722215.1** | ***ABCG2-like*** | **lost** |
| ***Pseudopodoces humilis*** | ***ABCG2*** | **XP_009507174.1** | ***ABCG2-like*** | **XP_005520621.1** |
| ***Pygoscelis adeliae*** | ***ABCG2*** | **XP_009325757.1** | ***ABCG2-like*** | **XP_009320667.1** |
| ***Struthio camelus australis*** | ***ABCG2*** | **XP_009674798.1** | ***ABCG2-like*** | **XP_009676190.1** |
| ***Sturnus vulgaris*** | ***ABCG2*** | **XP_014734486.1** | ***ABCG2-like*** | **XP_014730909.1** |
| ***Zonotrichia albicollis*** | ***ABCG2*** | **XP_014121273.1** | ***ABCG2-like*** | **XP_005481333.1** |

**Table S2 GenBank numbers of the bird *ABCG2* and *ABCG2-like* nucleic acid sequences obtained in our study.**

| **Avian species** | **Gene** | **Genebank** | **Gene** | **Genebank** |
| --- | --- | --- | --- | --- |
| ***Aquila chrysaetos canadensis*** | ***ABCG2*** | **XM_011600810.1** | ***ABCG2-like*** | **XM_011596882.1** |
| ***Haliaeetus albicilla*** | ***ABCG2*** | **XM_009912096.1** | ***ABCG2-like*** | **XM_011596882.1** |
| ***Haliaeetus leucocephalus*** | ***ABCG2*** | **XM_010569852.1** | ***ABCG2-like*** | **XM_010563516.1** |
| ***Anas platyrhynchos*** | ***ABCG2*** | **XM_013093252.2** | ***ABCG2-like*** | **XM_005025198.3** |
| ***Anser cygnoides domesticus*** | ***ABCG2*** | **XM_013190634.1** | ***ABCG2-like*** | **XM_013201381.1** |
| ***Apaloderma vittatum*** | ***ABCG2*** | **XM_009866965.1** | ***ABCG2-like*** | **XM_009876185.** |
| ***Aptenodytes forsteri*** | ***ABCG2*** | **XM_019472333.1** | ***ABCG2-like*** | **XM_009277713.1** |
| ***Apteryx australis mantelli*** | ***ABCG2*** | **XM_013957375.1** | ***ABCG2-like*** | **XM_013958841.1** |
| ***Balearica regulorum gibbericeps*** | ***ABCG2*** | **XM_010305474.1** | ***ABCG2-like*** | **XM_010307573.1** |
| ***Calidris pugnax*** | ***ABCG2*** | **XM_014961495.1** | ***ABCG2-like*** | **XM_014956362.1** |
| ***Calypte anna*** | ***ABCG2*** | **XM_008500807.1** | ***ABCG2-like*** | **XM_008502049.1** |
| ***Chaetura pelagica*** | ***ABCG2*** | **XM_010001747.1** | ***ABCG2-like*** | **XM_010000799.1** |
| ***Charadrius vociferus*** | ***ABCG2*** | **XM_009890747.1** | ***ABCG2-like*** | **XM_009888737.1** |
| ***Columba livia*** | ***ABCG2*** | **XM_021289056.1** | ***ABCG2-like*** | **XM_021293846.1** |
| ***Corvus brachyrhynchos*** | ***ABCG2*** | **XM_017744106.1** | ***ABCG2-like*** | **XM_017725952.1** |
| ***Corvus cornix cornix*** | ***ABCG2*** | **XM_019293215.2** | ***ABCG2-like*** | **XM_010411446.3** |
| ***Cuculus canorus*** | ***ABCG2*** | **XM_009559527.1** | ***ABCG2-like*** | **XM_009570704.1** |
| ***Cyanistes caeruleus*** | ***ABCG2*** | **XM_023926343.1** | ***ABCG2-like*** | **XM_023929539.1** |
| ***Egretta garzetta*** | ***ABCG2*** | **XM_009639994.1** | ***ABCG2-like*** | **XM_009642035.1** |
| ***Lonchura striata domestica*** | ***ABCG2*** | **XM_021526475.1** | ***ABCG2-like*** | **XM_021538873.1** |
| ***Taeniopygia guttata*** | ***ABCG2*** | **XM_002190304.3** | ***ABCG2-like*** | **XM_012574531.1** |
| ***Falco cherrug*** | ***ABCG2*** | **XM_014284406.1** | ***ABCG2-like*** | **XM_012574531.1** |
| ***Falco peregrinus*** | ***ABCG2*** | **XM_013296134.1** | ***ABCG2-like*** | **XM_005234589.2** |
| ***Ficedula albicollis*** | ***ABCG2*** | **XM_005044715.1** | ***ABCG2-like*** | **XM_016299567.1** |
| ***Serinus canaria*** | ***ABCG2*** | **XM_009101374.2** | ***ABCG2-like*** | **XM_009087357.2** |
| ***Geospiza fortis*** | ***ABCG2*** | **XM_014311945.1** | ***ABCG2-like*** | **XM_005416497.1** |
| ***Manacus vitellinus*** | ***ABCG2*** | **XM_018084592.1** | ***ABCG2-like*** | **lost** |
| ***Melopsittacus undulatus*** | ***ABCG2*** | **XM_005155227.1** | ***ABCG2-like*** | **XM_005153889.1** |
| ***Mesitornis unicolor*** | ***ABCG2*** | **XM_010186327.1** | ***ABCG2-like*** | **lost** |
| ***Nipponia nippon*** | ***ABCG2*** | **XM_009467634.1** | ***ABCG2-like*** | **XM_009465929.1** |
| ***Parus major*** | ***ABCG2*** | **XM_015624965.2** | ***ABCG2-like*** | **XM_015632945.1** |
| ***Pelecanus crispus*** | ***ABCG2*** | **XM_009490812.1** | ***ABCG2-like*** | **XM_009485486.1** |
| ***Phalacrocorax carbo*** | ***ABCG2*** | **XM_009511918.1** | ***ABCG2-like*** | **XM_009508879.1** |
| ***Meleagris gallopavo*** | ***ABCG2*** | **XM_019617611.1** | ***ABCG2-like*** | **lost** |
| ***Gallus gallus*** | ***ABCG2*** | **XM_025151575.1** | ***ABCG2-like*** | **lost** |
| *Coturnix japonica* | ***ABCG2*** | **XM_015866729.1** | ***ABCG2-like*** | **lost** |
| ***Pseudopodoces humilis*** | ***ABCG2*** | **XM_014254753.1** | ***ABCG2-like*** | **XM_005520564.2** |
| ***Pygoscelis adeliae*** | ***ABCG2*** | **XM_009327482.1** | ***ABCG2-like*** | **XM_009322392.1** |
| ***Struthio camelus australis*** | ***ABCG2*** | **XM_009676503.1** | ***ABCG2-like*** | **XM_009677895.1** |
| ***Sturnus vulgaris*** | ***ABCG2*** | **XM_014879000.1** | ***ABCG2-like*** | **XM_014875423.1** |
| ***Zonotrichia albicollis*** | ***ABCG2*** | **XM_014265798.2** | ***ABCG2-like*** | **XM_005481276.2** |
