# Supplementary material for: Molecular evolution of the ATP-binding cassette subfamily G member 2 gene subfamily and its paralogs in birds
Source: BMC Evol Biol. 2020 Jul 14;20:85. doi: 10.1186/s12862-020-01654-z (PMC7362505; doi:10.1186/s12862-020-01654-z)
Supplement: Supplementary file 2 — Additional file 2: Table S3. Nucleic acid sequences GenBank numbers of the ABCG2 gene subfamily members in outgroup species used in the phylogenetic analysis. Table S8 Nucleic acid sequences GenBank numbers of the outgroup ABCG2 gene subfamily member nucleic acid sequences used in the conversion analysis. [file 12862_2020_1654_MOESM2_ESM.docx]

| **Outgroups species** | **Gene** | **Genebank** |
| --- | --- | --- |
| ***Homo sapiens*** | ***ABCG2*** | **NP_001244315.1** |
| ***Mus caroli*** | ***ABCG2*** | **XP_021020806.1** |
| ***Alligator sinensis*** | ***ABCG2*** | **XP_006018174.1** |
| ***Alligator sinensis*** | ***ABCG2-like*** | **XP_006014851.1** |
| ***Xenopus laevis.L*** | ***ABCG2.L*** | **XP_018102834.1** |
| ***Xenopus laevis.S*** | ***ABCG2.S*** | **XP_018098921.1** |
| ***Chrysemys picta bellii*** | ***ABCG2*** | **XP_005292068.1** |
| ***Chrysemys picta bellii*** | ***ABCG2-like*** | **XP_005292067.1** |
| ***Danio rerio*** | ***ABCG2a*** | **NP_001036240.1** |
| ***Danio rerio*** | ***ABCG2b*** | **NP_001034155.1** |
| ***Danio rerio*** | ***ABCG2c*** | **NP_001034728.2** |
| ***Danio rerio*** | ***ABCG2d*** | **NM_001042772.1** |

**Table S3 Nucleic acid sequences GenBank numbers of the *ABCG2* gene subfamily members in outgroup species used in the phylogenetic analysis.**

**Table S8** **Nucleic acid sequences GenBank numbers of the outgroup *ABCG2* gene subfamily member nucleic acid sequences used in the conversion analysis.**

| **Outgroups species** | **Gene** | **Genebank** |
| --- | --- | --- |
| ***Homo sapiens*** | ***ABCG2*** | **NM_001257386.2** |
| ***Mus caroli*** | ***ABCG2*** | **XM_021165147.1** |
| ***Xenopus laevis.L*** | ***ABCG2.L*** | **NM_001097672.1** |
| ***Xenopus laevis.S*** | ***ABCG2.S*** | **XM_018243432.1** |
| ***Chrysemys picta bellii*** | ***ABCG2*** | **XM_005292011.3** |
| ***Chrysemys picta bellii*** | ***ABCG2-like*** | **XM_005292010.3** |
| ***Alligator sinensis*** | ***ABCG2*** | **XM_006018112.3** |
| ***Alligator sinensis*** | ***ABCG2-like*** | **XM_006014789.3** |
