# Supplementary material for: Molecular evolution of the ATP-binding cassette subfamily G member 2 gene subfamily and its paralogs in birds
Source: BMC Evol Biol. 2020 Jul 14;20:85. doi: 10.1186/s12862-020-01654-z (PMC7362505; doi:10.1186/s12862-020-01654-z)
Supplement: Supplementary file 3 — Additional file 3: Table S4. Likelihood ratio test statistics for evaluation of model fit in ABCG2 gene. Table S5 Likelihood ratio test statistics for evaluation of model fit in ABCG2-like gene. [file 12862_2020_1654_MOESM3_ESM.docx]

**Table S4** Likelihood ratio test statistics for evaluation of model fit in *ABCG2* gene.

| Model comparison | df | 2△InL | LRT p |
| --- | --- | --- | --- |
| M0 vs M3 | 2 | 14590 | 0.00E+00 |
| M1a vs M2a | 2 | 50 | 1.39E-11 |
| M7 vs M8 | 2 | 86 | 0.00E+00 |

**Table S5** Likelihood ratio test statistics for evaluation of model fit in *ABCG2-like* gene.

| Model comparison | df | 2△InL | LRT p |
| --- | --- | --- | --- |
| M0 vs M3 | 2 | 12292 | 0.00E+00 |
| M1a vs M2a | 2 | 26 | 2.26E-06 |
| M7 vs M8 | 2 | 44 | 2.79E-10 |
