# Supplementary material for: Molecular evolution of the ATP-binding cassette subfamily G member 2 gene subfamily and its paralogs in birds
Source: BMC Evol Biol. 2020 Jul 14;20:85. doi: 10.1186/s12862-020-01654-z (PMC7362505; doi:10.1186/s12862-020-01654-z)
Supplement: Supplementary file 4 — Additional file 4: Table S6. Positive selection sites of ABCG2 gene. Table S7 Positive selection sites of ABCG2-like gene. [file 12862_2020_1654_MOESM4_ESM.docx]

**Table S6 Positive selection sites of *ABCG2* gene.**

| **Model code** | **positive selection sites** |
| --- | --- |
| **M2a(positive selection)** | 134 S, **401 H**, 705 -, 725 M, **726 W** |
| **M8 (beta&ω>1)** | **87 S**, **96 K**, 104 -, 122 V, **134 S**, **349 T**, 387 D, **401 H**, 696 S, 703 -, **705 -**,  **725 M**, **726 W**, 741 V |

Note: Amino acid positions ars based on the *Anas platyrhynchos* *ABCG2* gene (XM_013093252.2), positive selection sites p>95%, bold fonts p>99%

**Table S7 Positive selection sites of *ABCG2-like* gene.**

| **Model code** | **positive selection sites** |
| --- | --- |
| **M2a(positive selection)** | 32 -, 37-, 38- |
| **M8 (beta&ω>1)** | **87 A**, 429 A, **694 V**, 715 N |

Note: Amino acid positions ars based on the *Anas platyrhynchos* *ABCG2-like* gene (XM_005025198.3), positive selection sites p>95%, bold fonts p>99%
