# Supplementary material for: Molecular evolution of the ATP-binding cassette subfamily G member 2 gene subfamily and its paralogs in birds
Source: BMC Evol Biol. 2020 Jul 14;20:85. doi: 10.1186/s12862-020-01654-z (PMC7362505; doi:10.1186/s12862-020-01654-z)
Supplement: Supplementary file 6 — Additional file 6. Prediction results of the protein transmembrane structure of ABCG2 gene subfamily members. [file 12862_2020_1654_MOESM6_ESM.pdf]

Avian species ABCG2

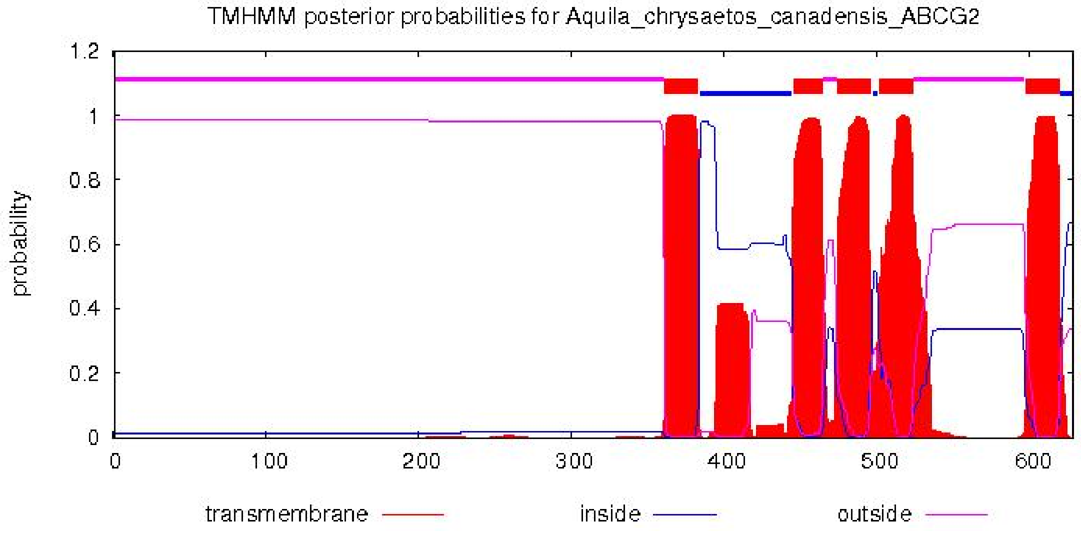

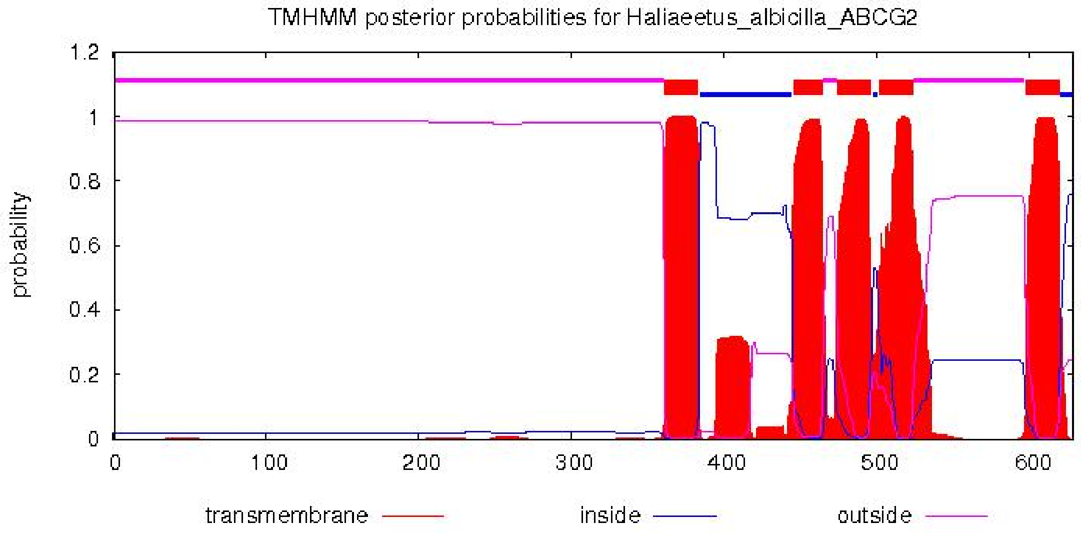

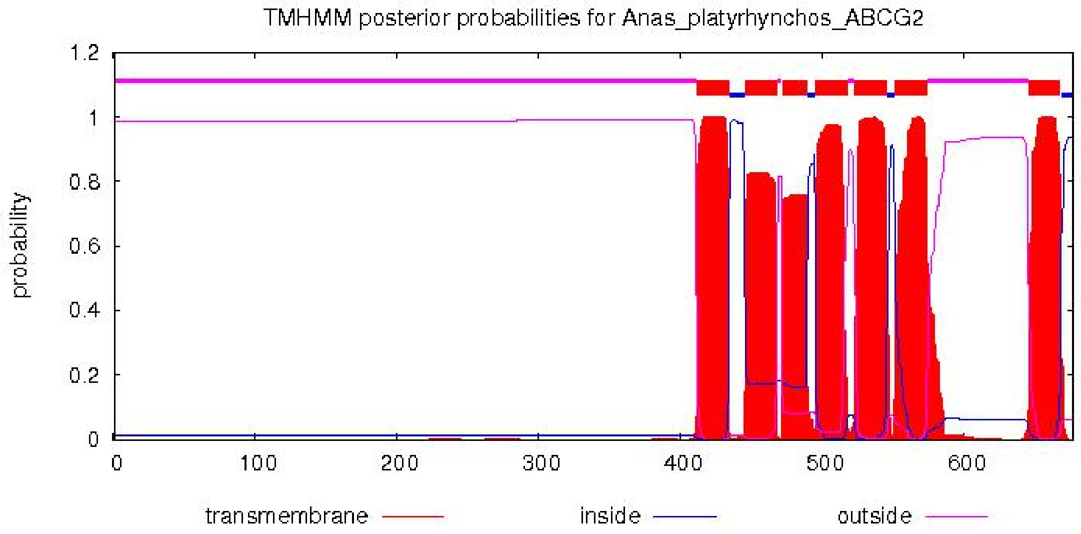

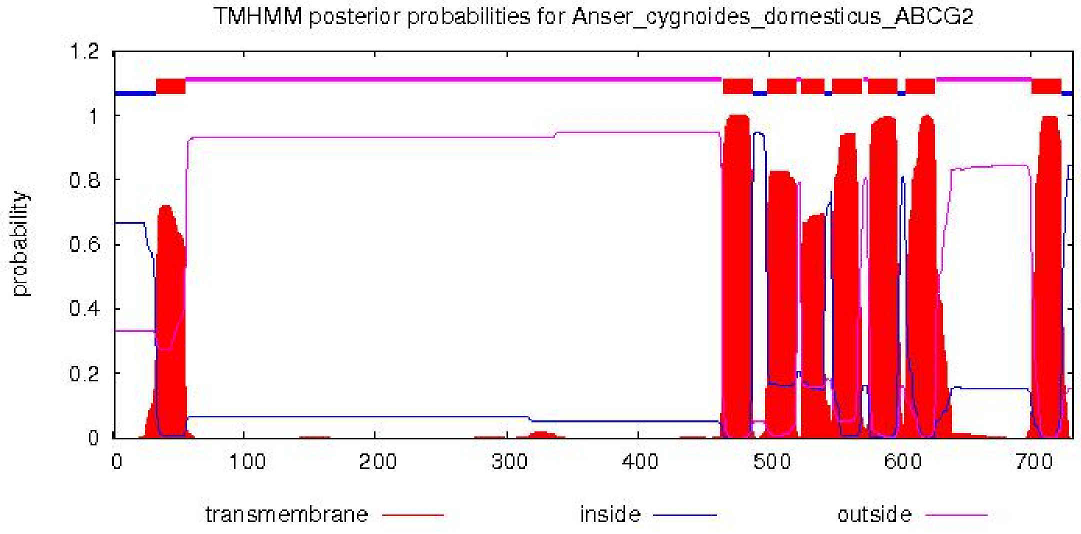
TMHMM posterior probabilities for Anser_cygnoides_domesticus_ABCG2
probability
1.2
1
0.8
0.6
0.4
0.2
0
0
100
200
300
400
500
600
700
transmembrane
inside
outside

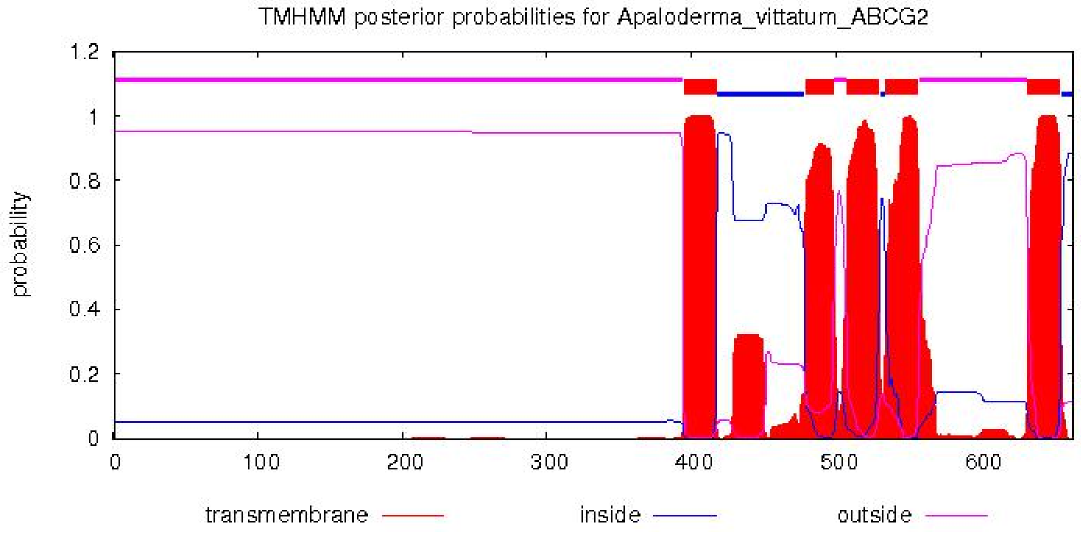
TMHMM posterior probabilities for Apaloderma_vittatum_ABCG2
probability
1.2
1
0.8
0.6
0.4
0.2
0
0
100
200
300
400
500
600
transmembrane
inside
outside

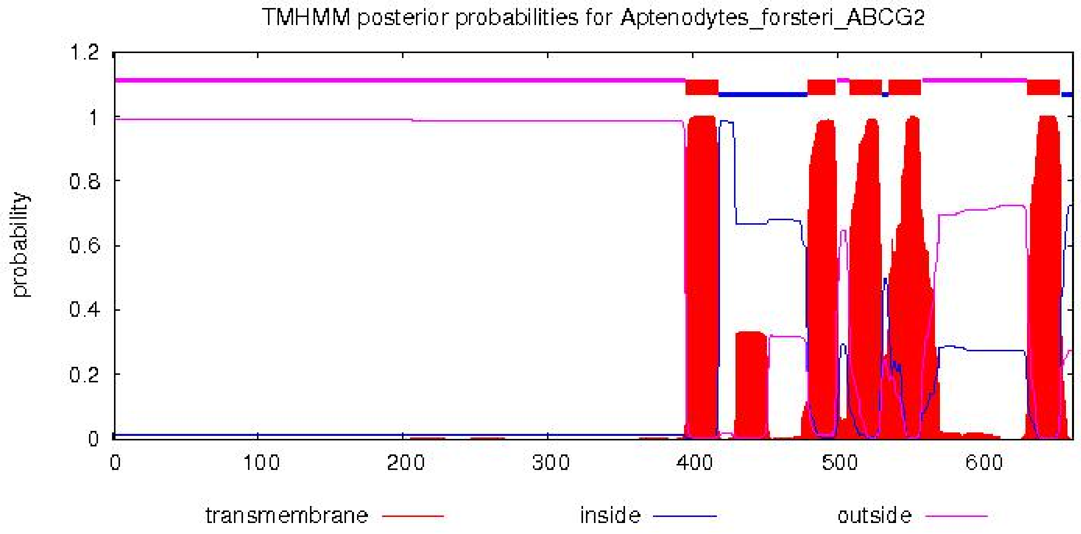
TMHMM posterior probabilities for Aptenodytes_forsteri_ABCG2
probability
1.2
1
0.8
0.6
0.4
0.2
0
0
100
200
300
400
500
600
transmembrane
inside
outside

TMHMM posterior probabilities for Apteryx_australis_mantelli_ABCG2

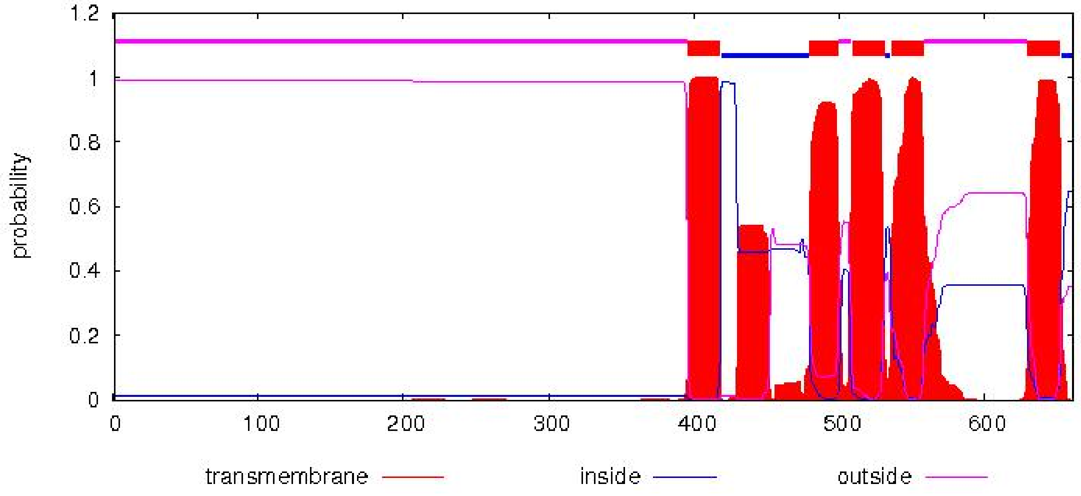

TMHMM posterior probabilities for Balearica_regulorum_gibbericeps_ABCG2

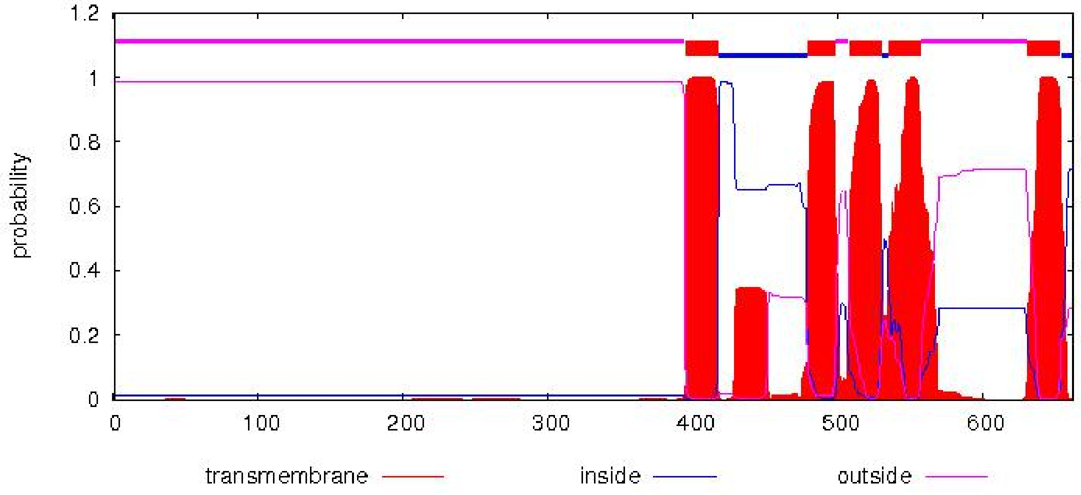

TMHMM posterior probabilities for Calidris_pugnax_ABCG2

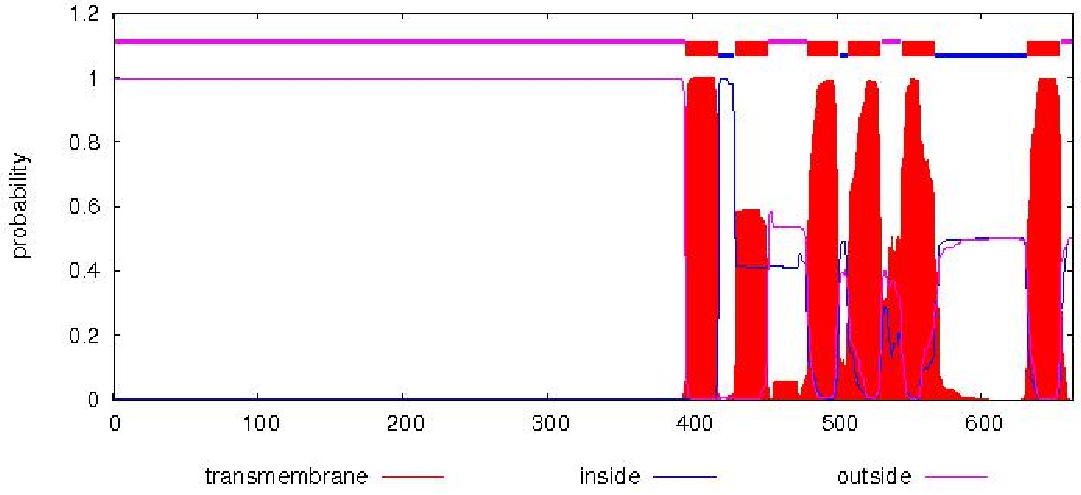

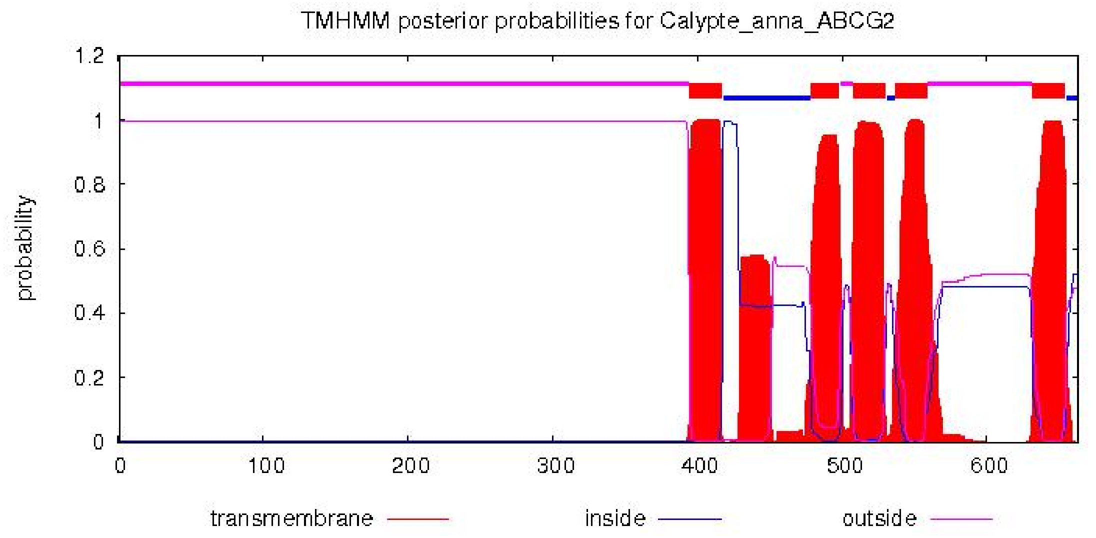

TMHMM posterior probabilities for Calypte_anna_ABCG2
probability
1.2
1
0.8
0.6
0.4
0.2
0
0
100
200
300
400
500
600
transmembrane
inside
outside

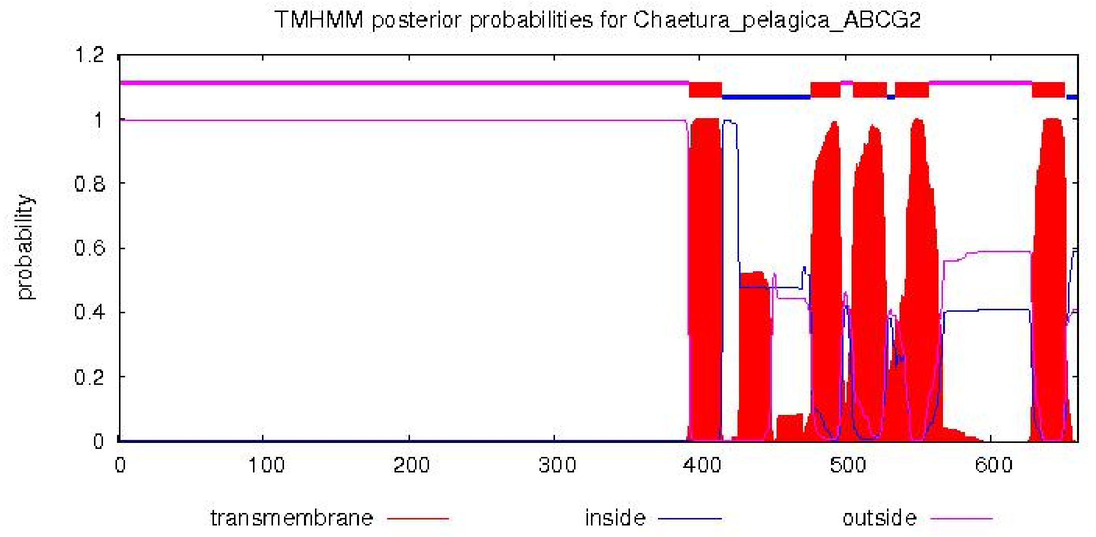

TMHMM posterior probabilities for Chaetura_pelagica_ABCG2
probability
1.2
1
0.8
0.6
0.4
0.2
0
0
100
200
300
400
500
600
transmembrane
inside
outside

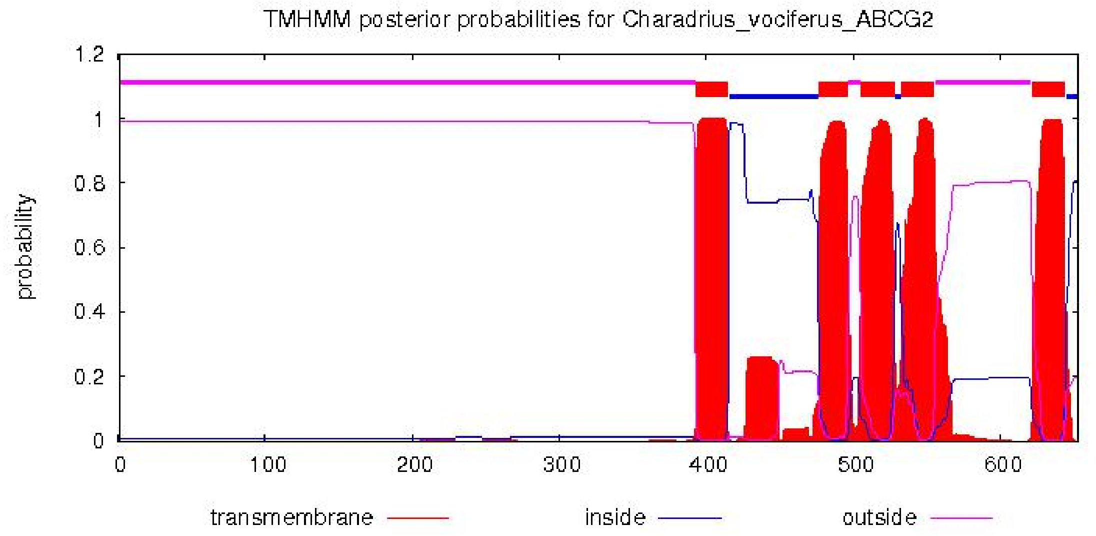

TMHMM posterior probabilities for Charadrius_vociferus_ABCG2
probability
1.2
1
0.8
0.6
0.4
0.2
0
0
100
200
300
400
500
600
transmembrane
inside
outside

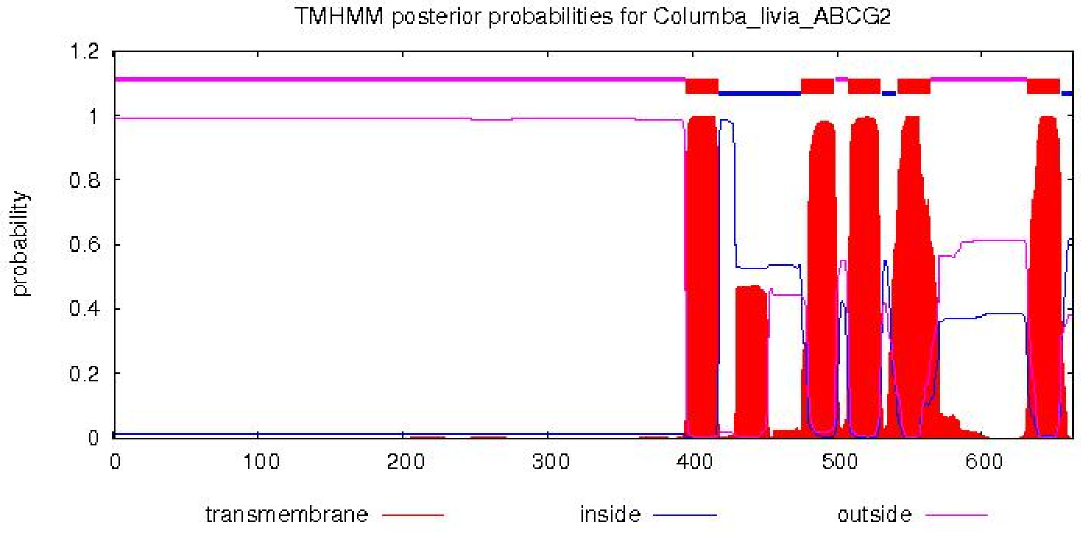

TMHMM posterior probabilities for Columba_livia_ABCG2
probability
1.2
1
0.8
0.6
0.4
0.2
0
0
100
200
300
400
500
600
transmembrane
inside
outside

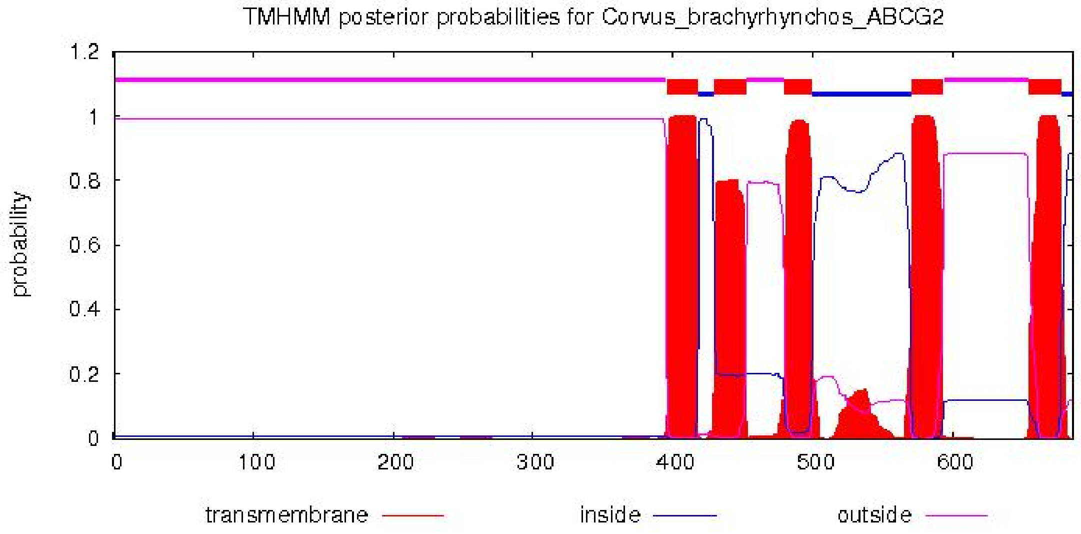

TMHMM posterior probabilities for Corvus_brachyrhynchos_ABCG2
probability
1.2
1
0.8
0.6
0.4
0.2
0
0
100
200
300
400
500
600
transmembrane
inside
outside

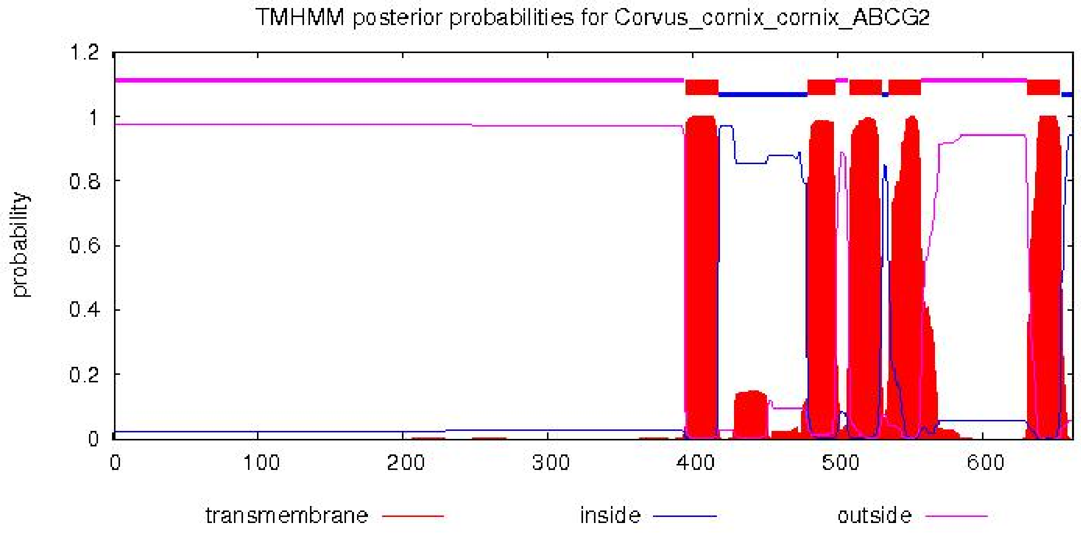

TMHMM posterior probabilities for Corvus_cornix_cornix_ABCG2
probability
1.2
1
0.8
0.6
0.4
0.2
0
0
100
200
300
400
500
600
transmembrane
inside
outside

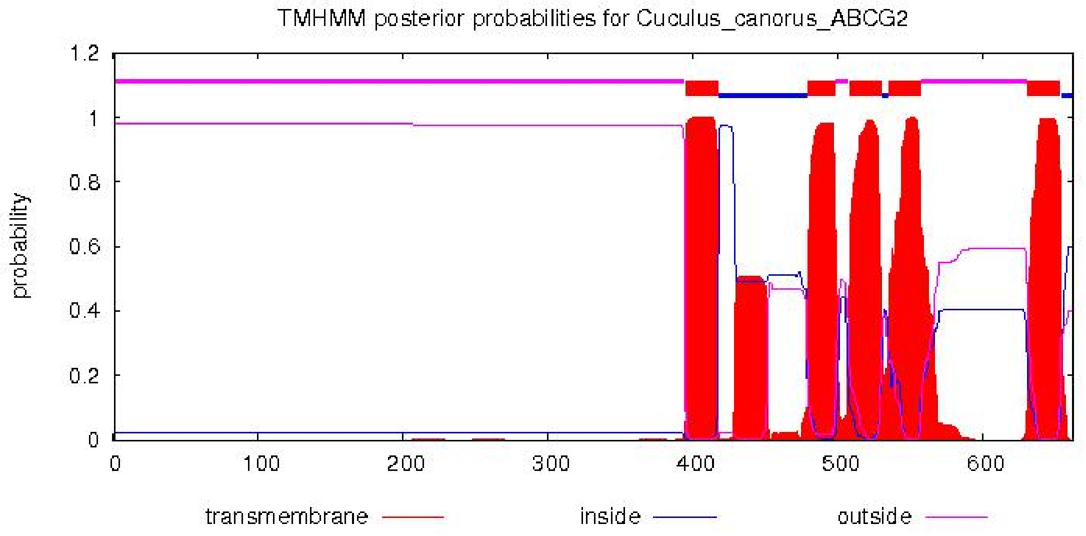
TMHMM posterior probabilities for Cuculus_canorus_ABCG2
probability
1.2
1
0.8
0.6
0.4
0.2
0
0
100
200
300
400
500
600
transmembrane
inside
outside

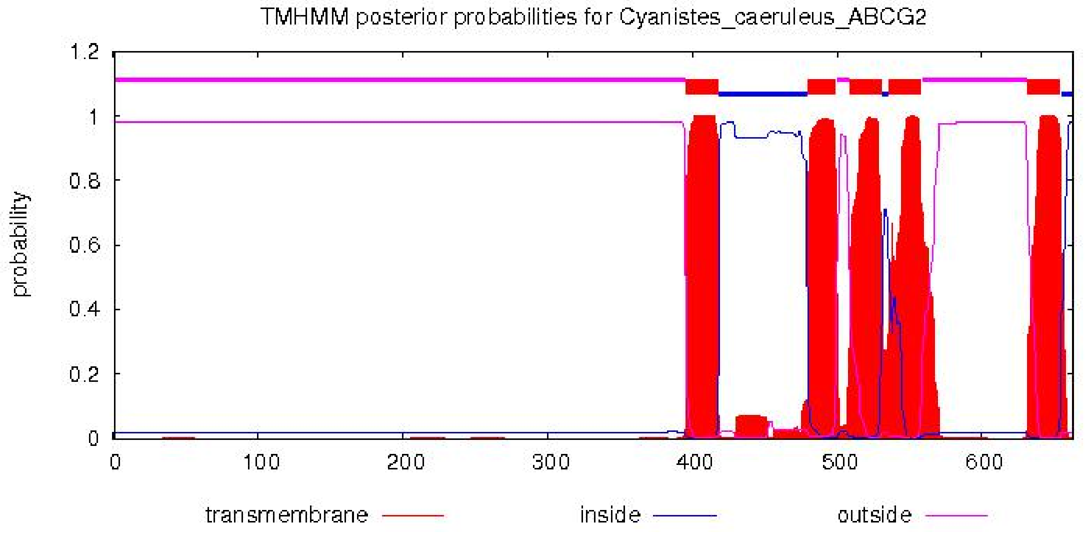
TMHMM posterior probabilities for Cyanistes_caeruleus_ABCG2
probability
1.2
1
0.8
0.6
0.4
0.2
0
0
100
200
300
400
500
600
transmembrane
inside
outside

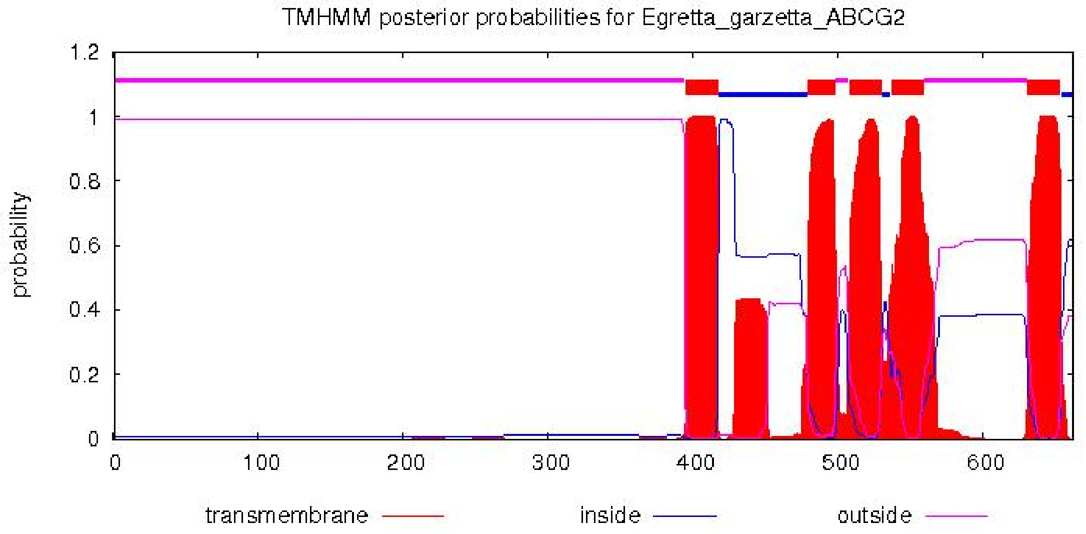
TMHMM posterior probabilities for Egretta_garzetta_ABCG2
probability
1.2
1
0.8
0.6
0.4
0.2
0
0
100
200
300
400
500
600
transmembrane
inside
outside

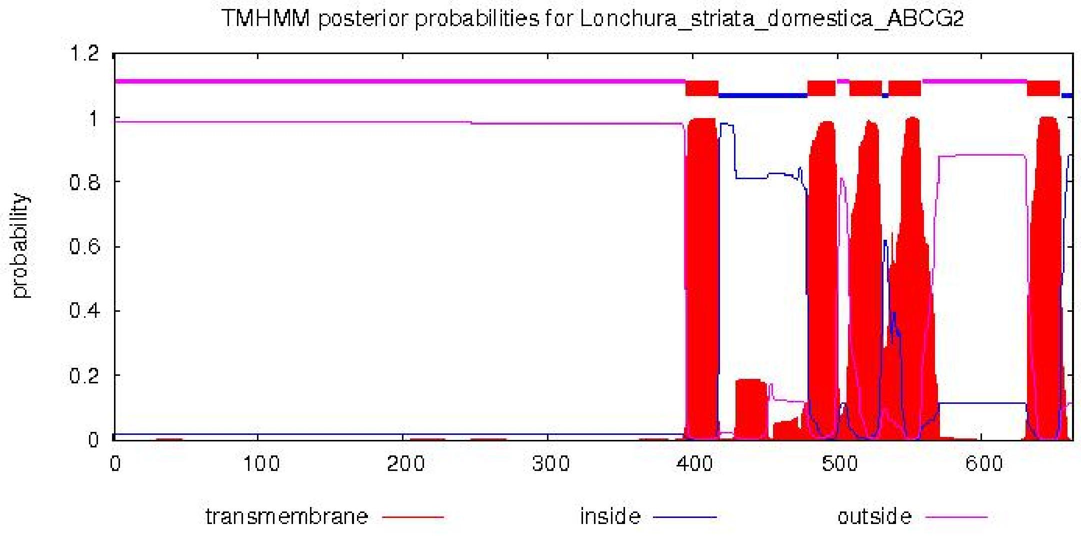
TMHMM posterior probabilities for Lonchura_striata_domestica_ABCG2
probability
1.2
1
0.8
0.6
0.4
0.2
0
0
100
200
300
400
500
600
transmembrane
inside
outside

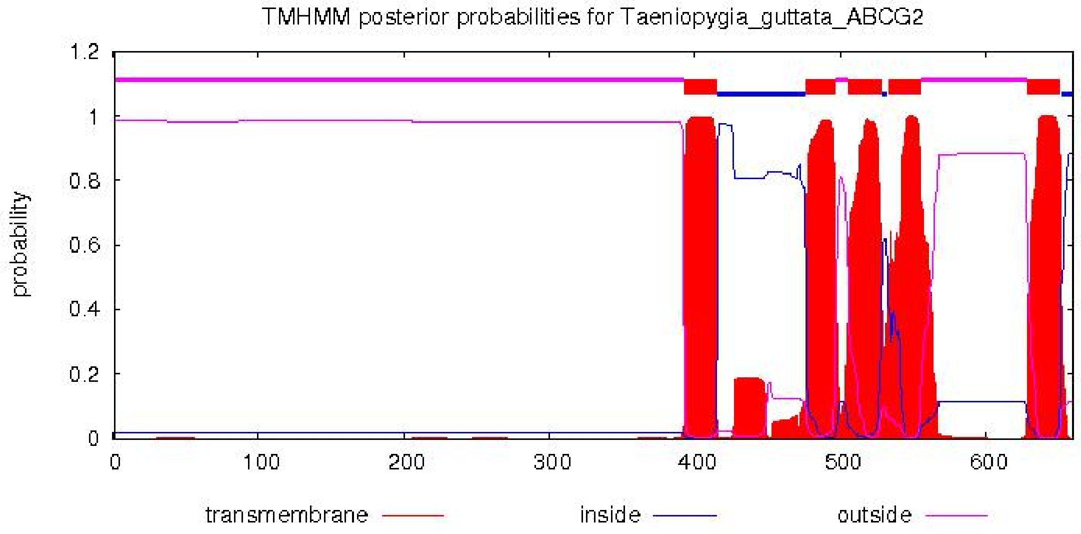
TMHMM posterior probabilities for Taeniopygia_guttata_ABCG2
probability
1.2
1
0.8
0.6
0.4
0.2
0
0
100
200
300
400
500
600
transmembrane
inside
outside

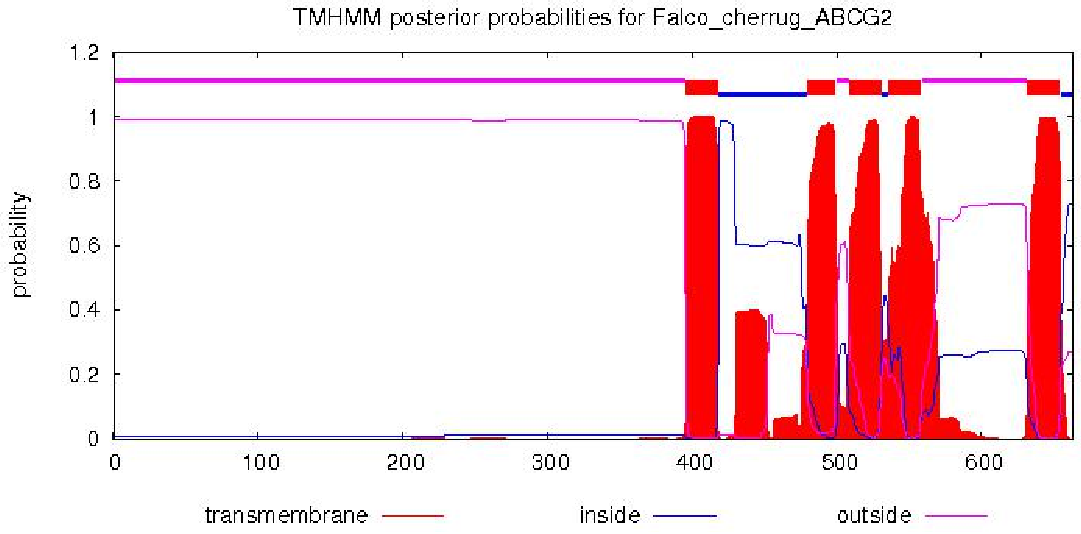
TMHMM posterior probabilities for Falco_cherrug_ABCG2
probability
1.2
1
0.8
0.6
0.4
0.2
0
0
100
200
300
400
500
600
transmembrane
inside
outside

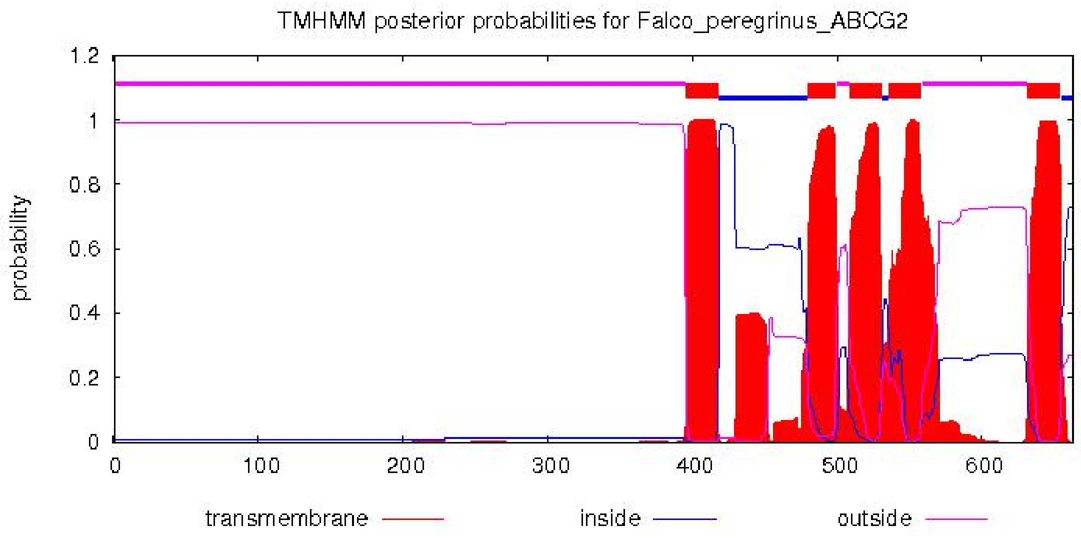
TMHMM posterior probabilities for Falco_peregrinus_ABCG2
probability
transmembrane
inside
outside

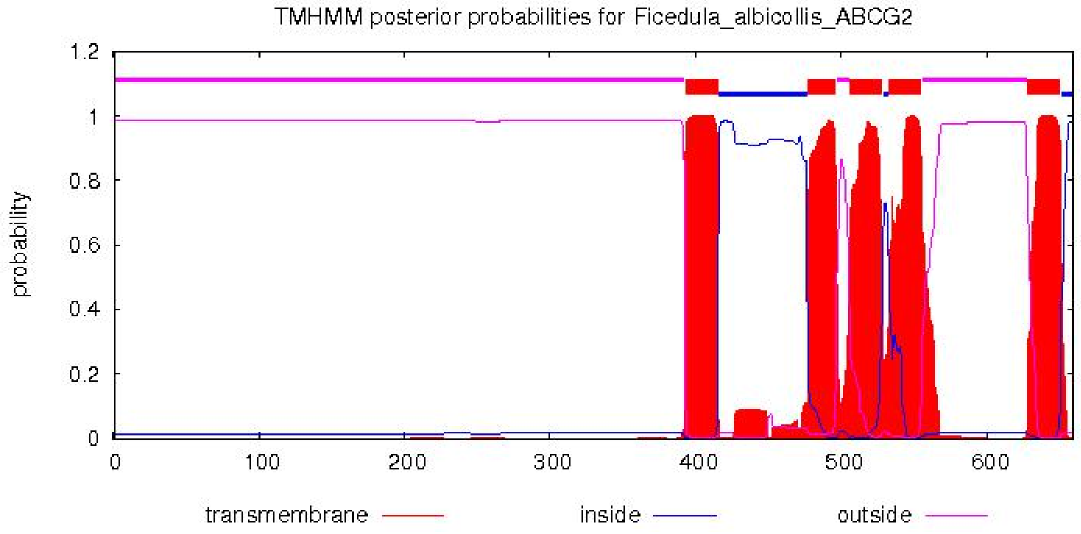
TMHMM posterior probabilities for Ficedula_albicollis_ABCG2
probability
transmembrane
inside
outside

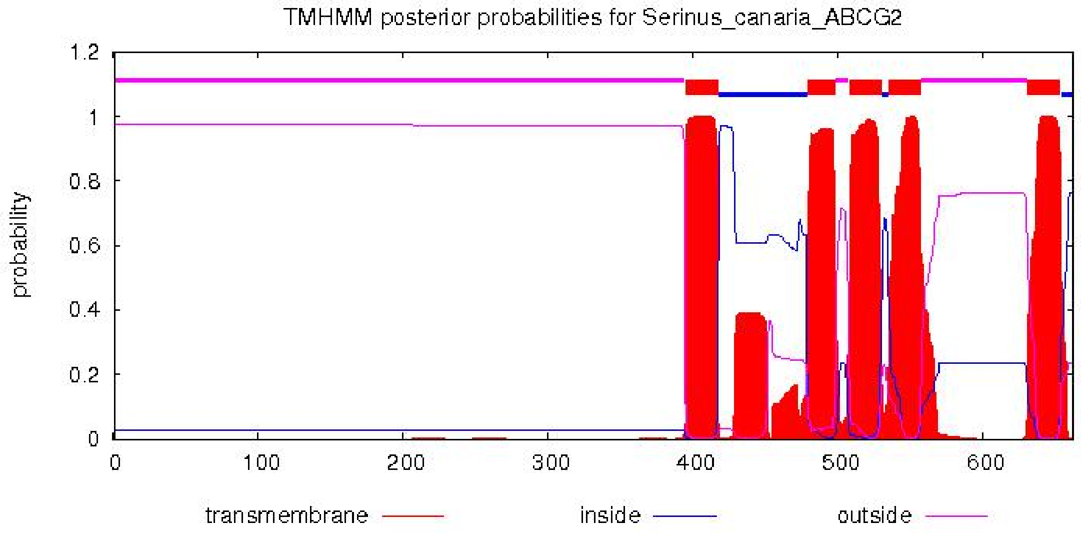
TMHMM posterior probabilities for Serinus_canaria_ABCG2
probability
transmembrane
inside
outside

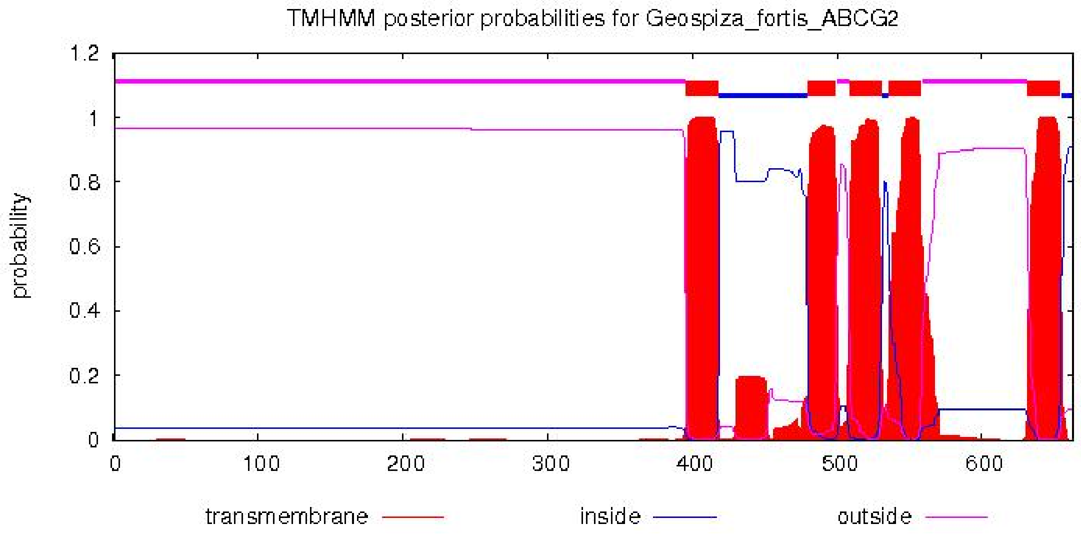

TMHMM posterior probabilities for Geospiza_fortis_ABCG2
probability
1.2
1
0.8
0.6
0.4
0.2
0
0
100
200
300
400
500
600
transmembrane
inside
outside

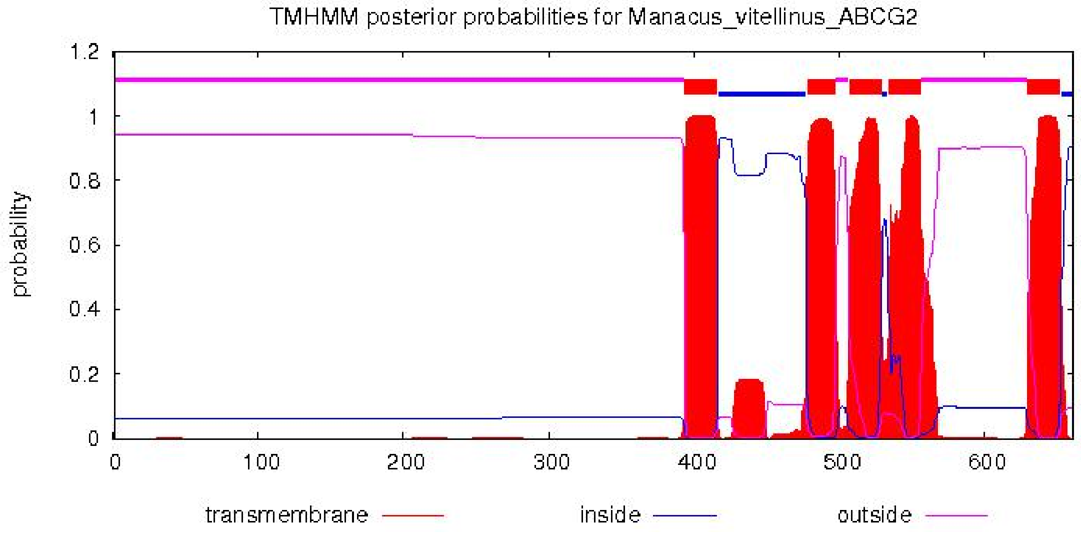

TMHMM posterior probabilities for Manacus_vitellinus_ABCG2
probability
1.2
1
0.8
0.6
0.4
0.2
0
0
100
200
300
400
500
600
transmembrane
inside
outside

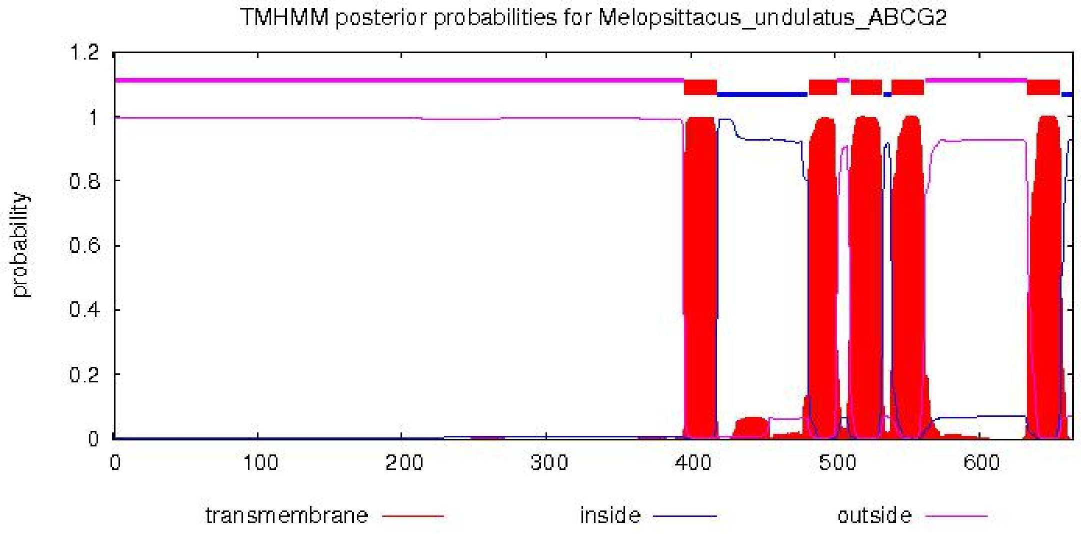

TMHMM posterior probabilities for Melopsittacus_undulatus_ABCG2
probability
1.2
1
0.8
0.6
0.4
0.2
0
0
100
200
300
400
500
600
transmembrane
inside
outside

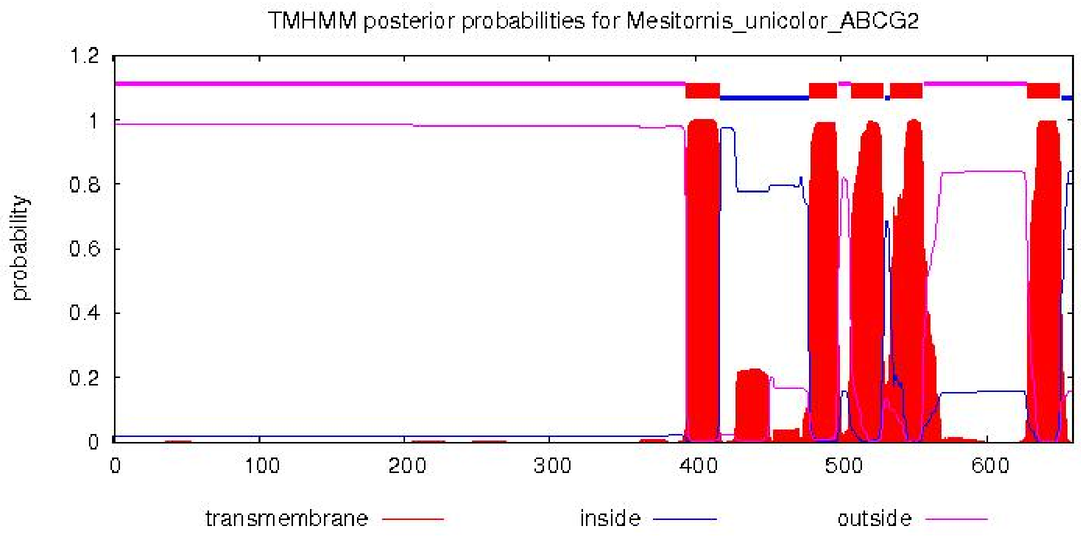
TMHMM posterior probabilities for Mesitornis_unicolor_ABCG2
probability
transmembrane
inside
outside

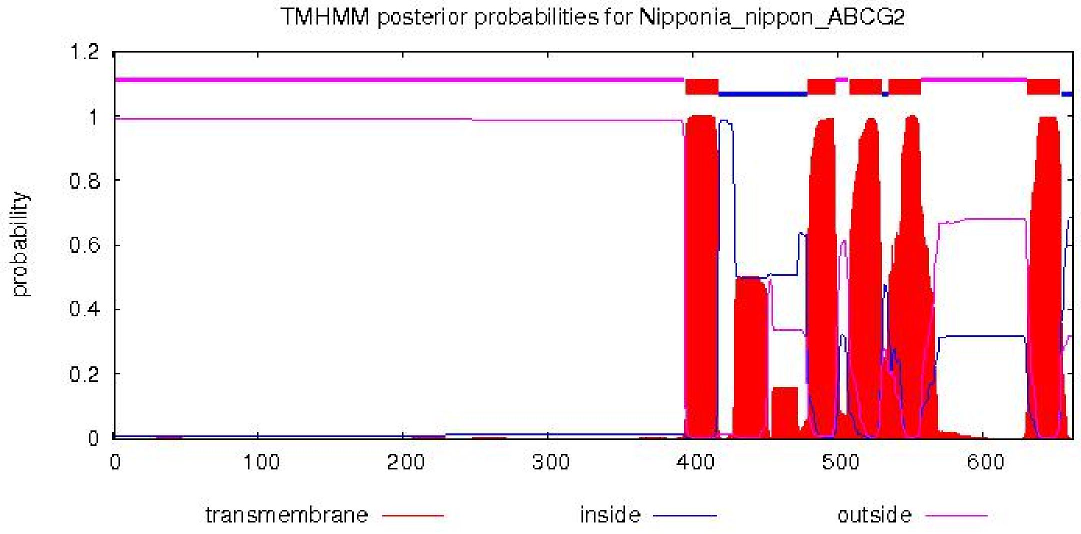
TMHMM posterior probabilities for Nipponia_nippon_ABCG2
probability
transmembrane
inside
outside

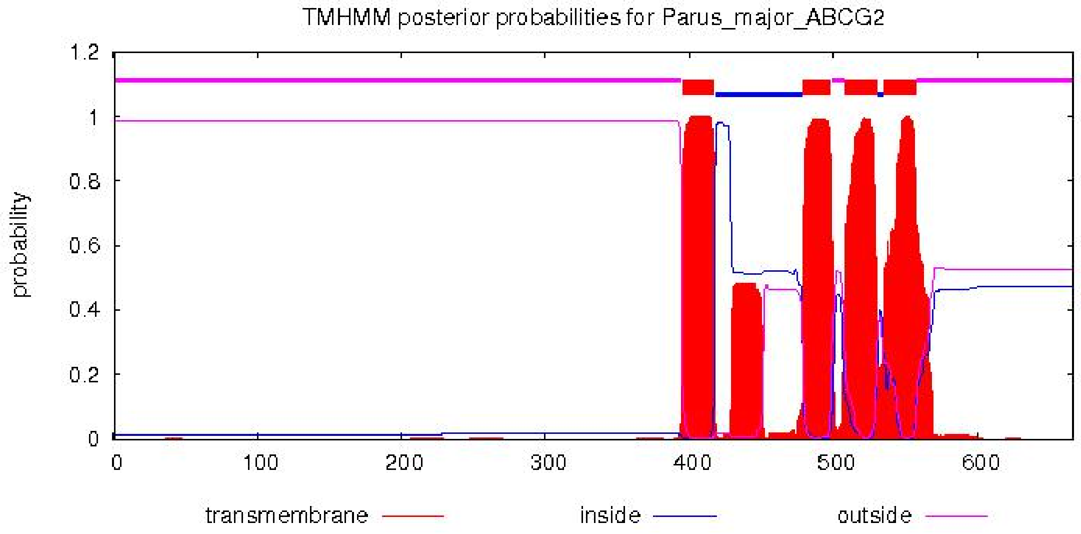
TMHMM posterior probabilities for Parus_major_ABCG2
probability
transmembrane
inside
outside

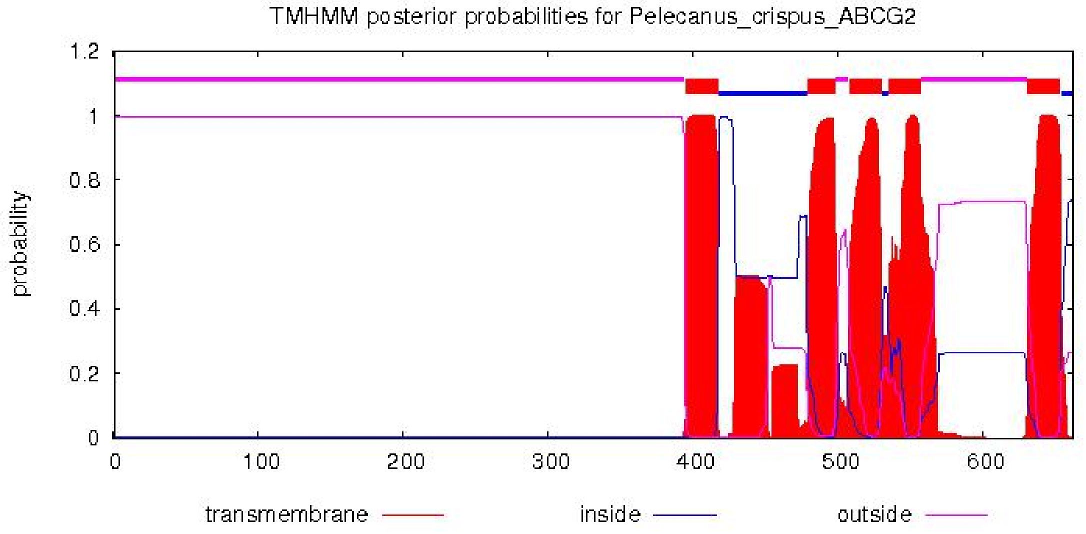
TMHMM posterior probabilities for Pelecanus_crispus_ABCG2
probability
1.2
1
0.8
0.6
0.4
0.2
0
0
100
200
300
400
500
600
transmembrane
inside
outside

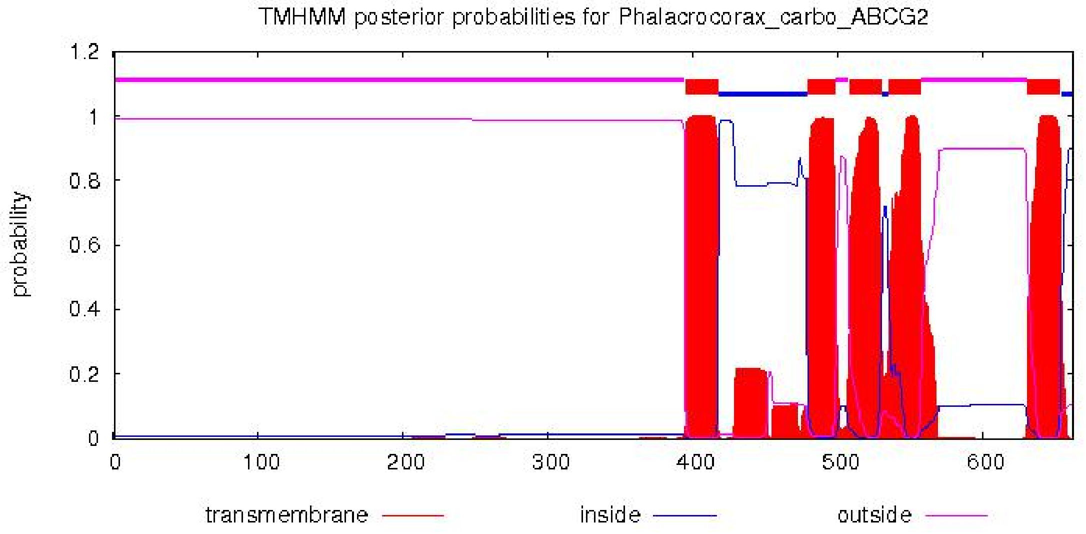
TMHMM posterior probabilities for Phalacrocorax_carbo_ABCG2
probability
1.2
1
0.8
0.6
0.4
0.2
0
0
100
200
300
400
500
600
transmembrane
inside
outside

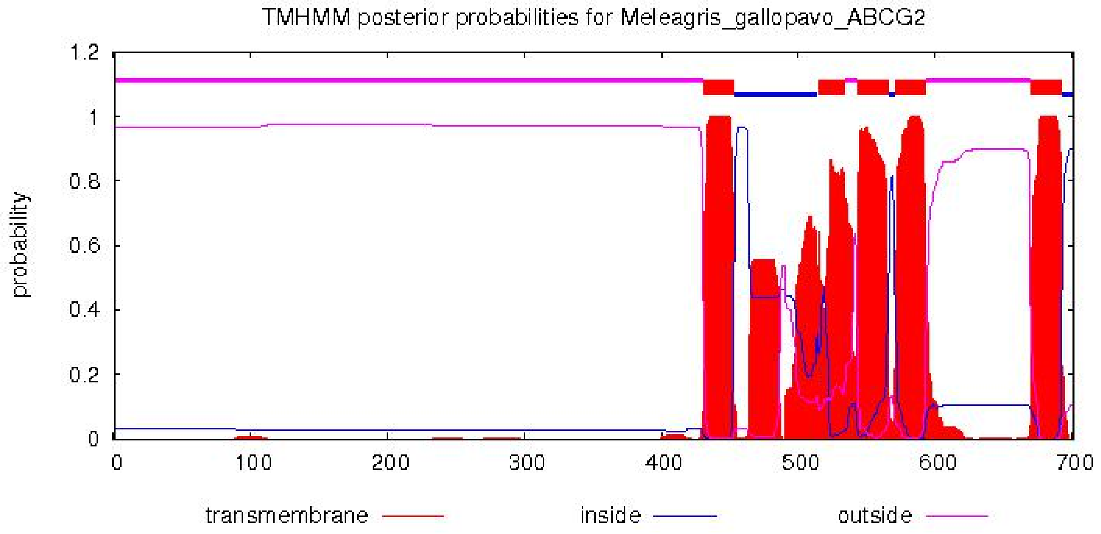
TMHMM posterior probabilities for Meleagris_gallopavo_ABCG2
probability
1.2
1
0.8
0.6
0.4
0.2
0
0
100
200
300
400
500
600
700
transmembrane
inside
outside

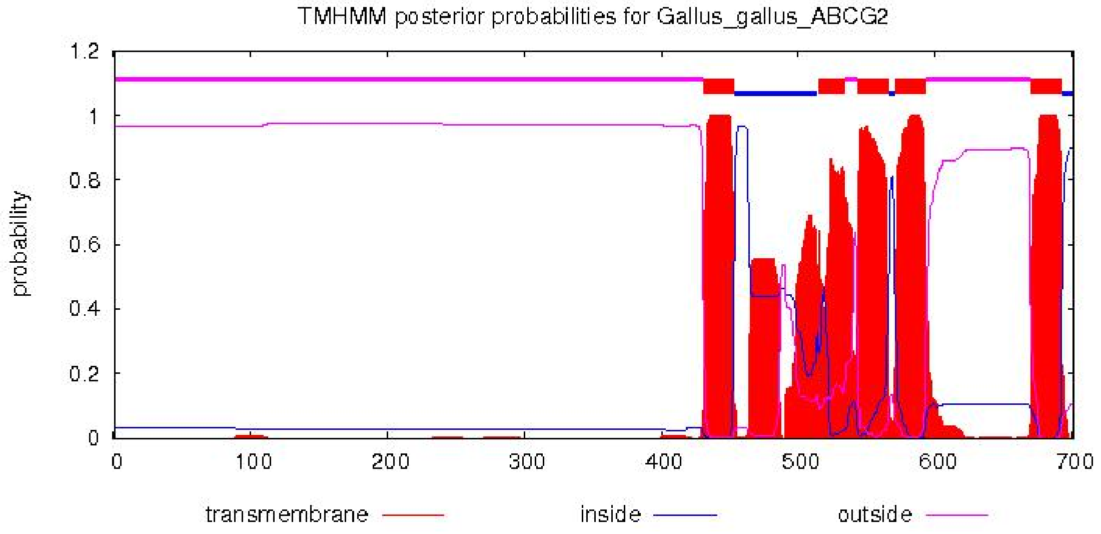
TMHMM posterior probabilities for Gallus_gallus_ABCG2
probability
1.2
1
0.8
0.6
0.4
0.2
0
0
100
200
300
400
500
600
700
transmembrane
inside
outside

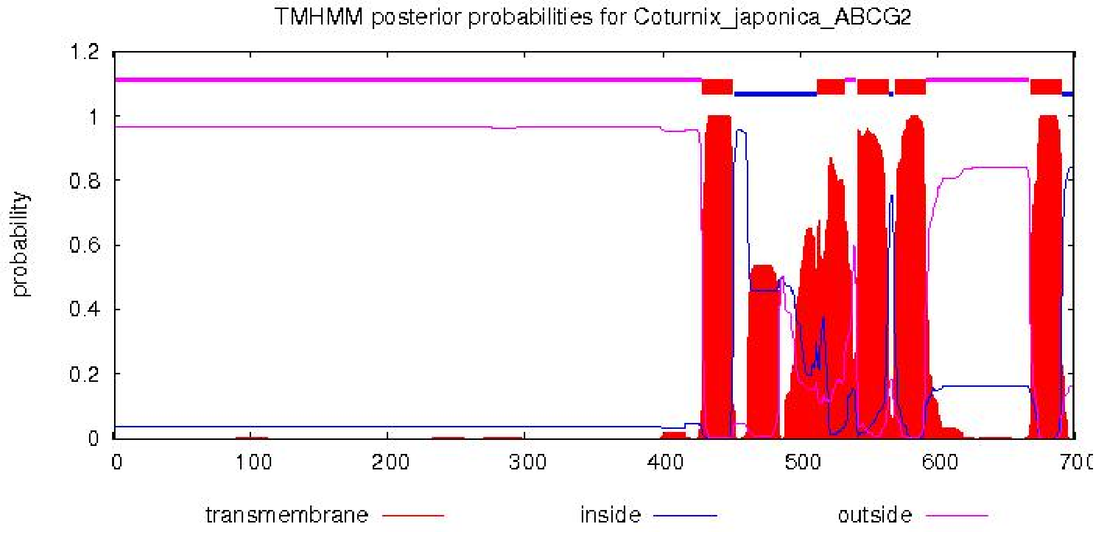
TMHMM posterior probabilities for Coturnix_japonica_ABCG2
probability
1.2
1
0.8
0.6
0.4
0.2
0
0
100
200
300
400
500
600
transmembrane
inside
outside

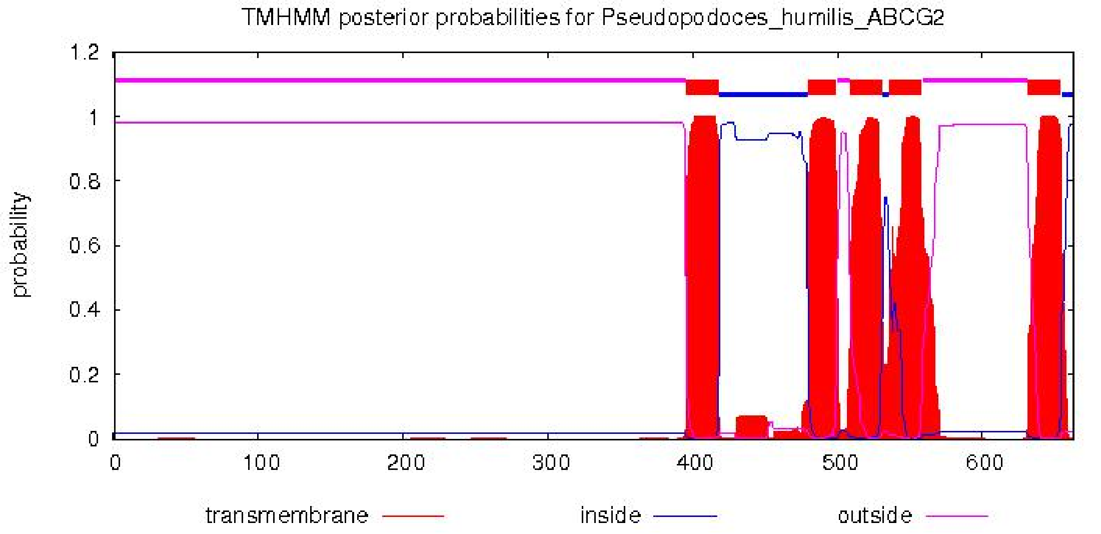
TMHMM posterior probabilities for Pseudopodoces_humilis_ABCG2
probability
1.2
1
0.8
0.6
0.4
0.2
0
0
100
200
300
400
500
600
transmembrane
inside
outside

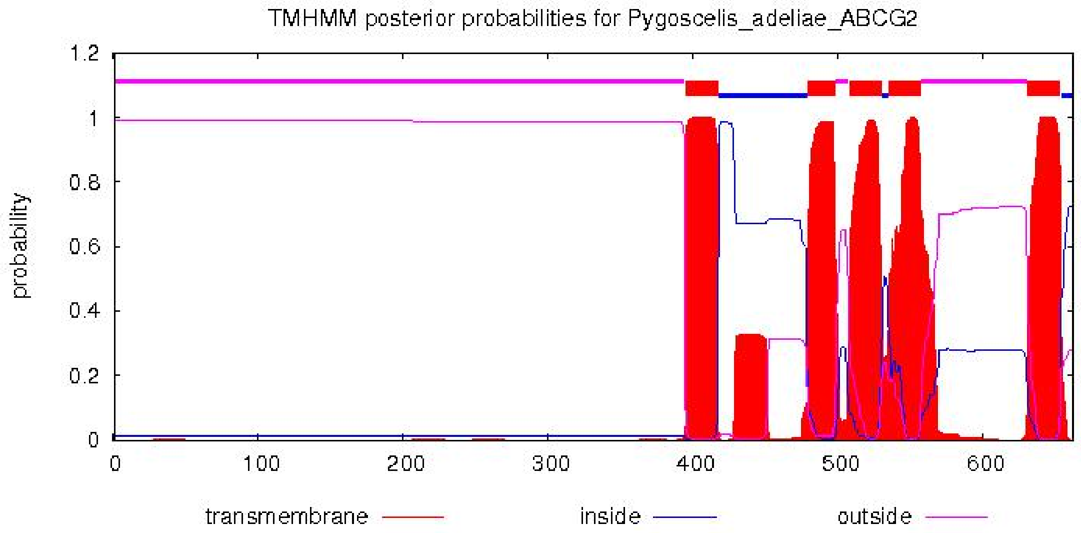
TMHMM posterior probabilities for Pygoscelis_adeliae_ABCG2
probability
1.2
1
0.8
0.6
0.4
0.2
0
0
100
200
300
400
500
600
transmembrane
inside
outside

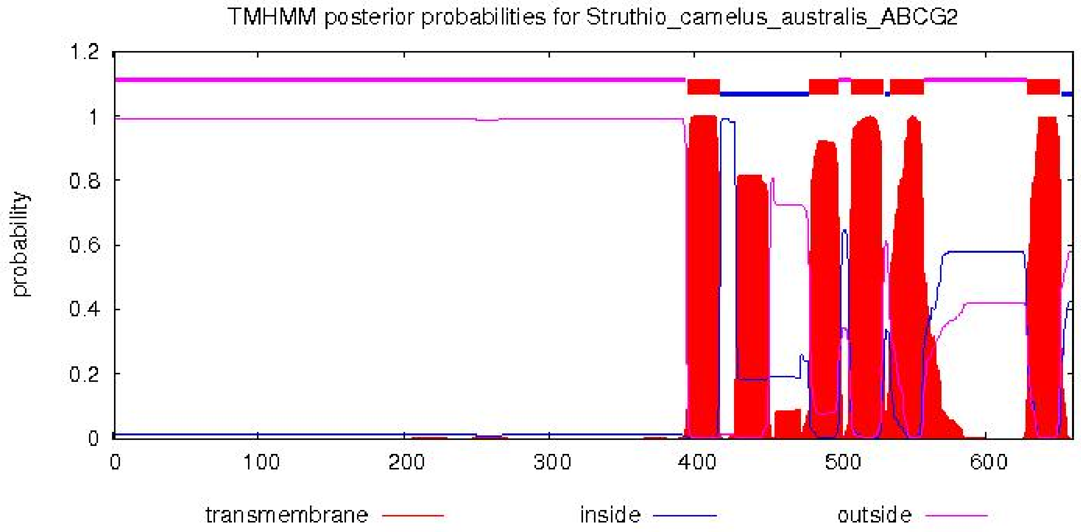
TMHMM posterior probabilities for Struthio_camelus_australis_ABCG2
probability
1.2
1
0.8
0.6
0.4
0.2
0
0
100
200
300
400
500
600
transmembrane
inside
outside

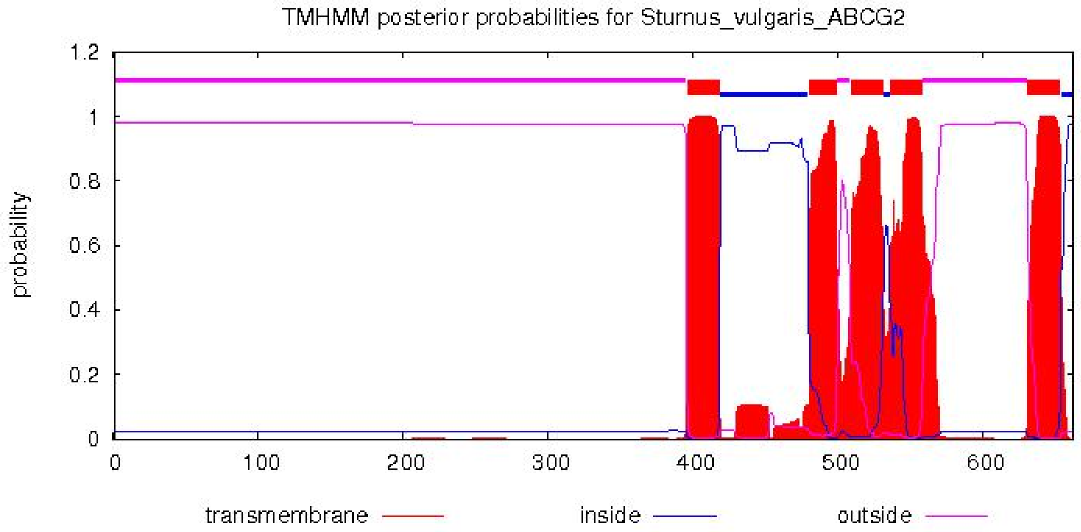
TMHMM posterior probabilities for Sturnus_vulgaris_ABCG2
probability
1.2
1
0.8
0.6
0.4
0.2
0
0
100
200
300
400
500
600
transmembrane
inside
outside

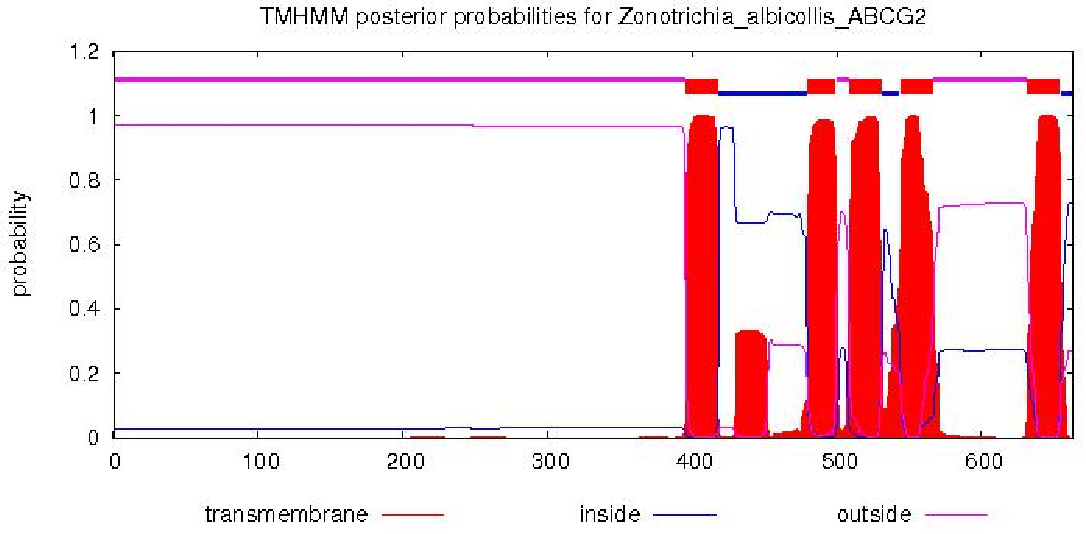

Avian species ABCG2-like(ABCG2-L)

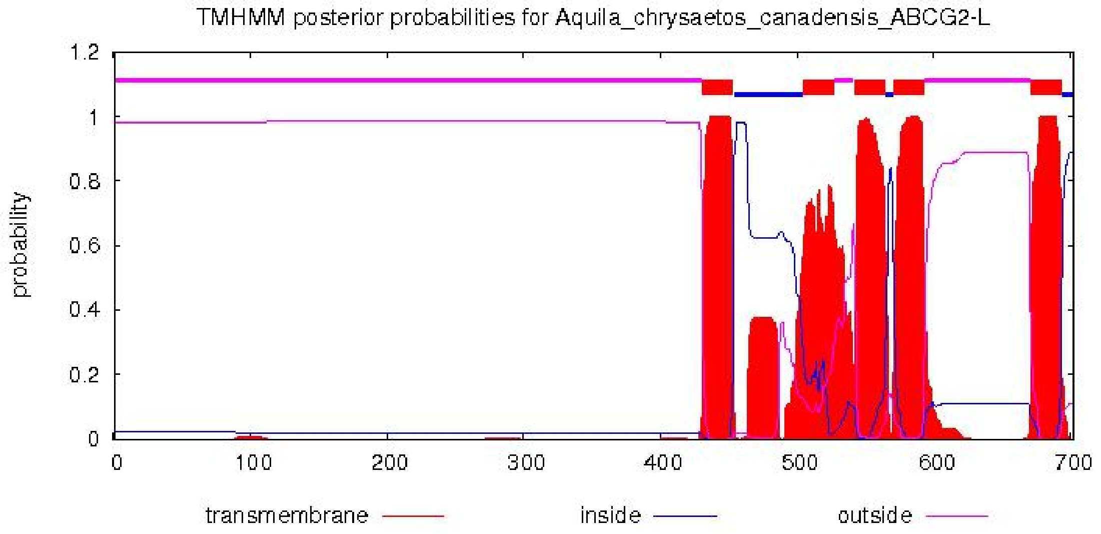

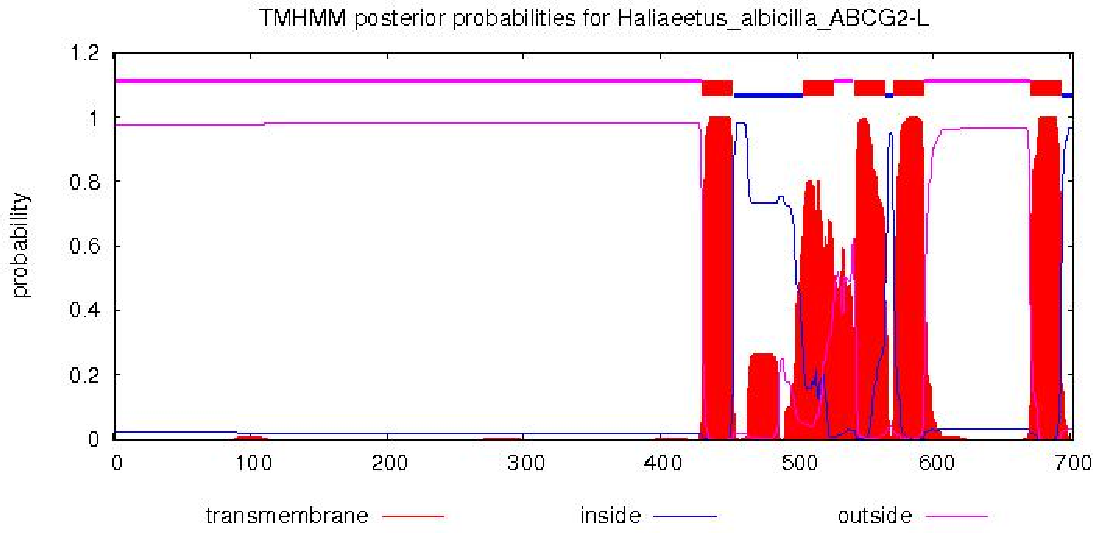

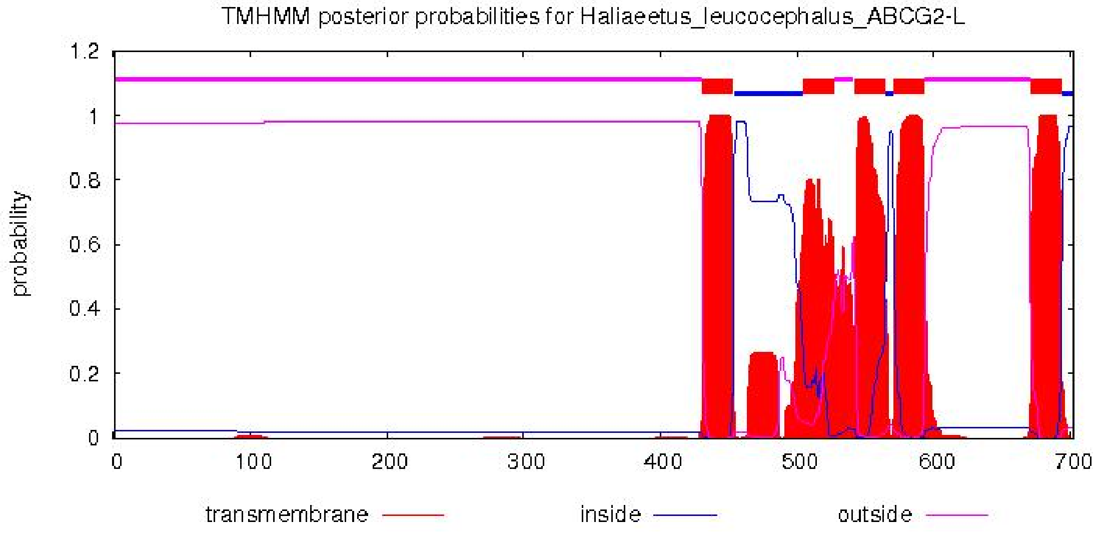
TMHMM posterior probabilities for Haliaeetus_leucocephalus_ABCG2-L
probability
1.2
1
0.8
0.6
0.4
0.2
0
0
100
200
300
400
500
600
700
transmembrane
inside
outside

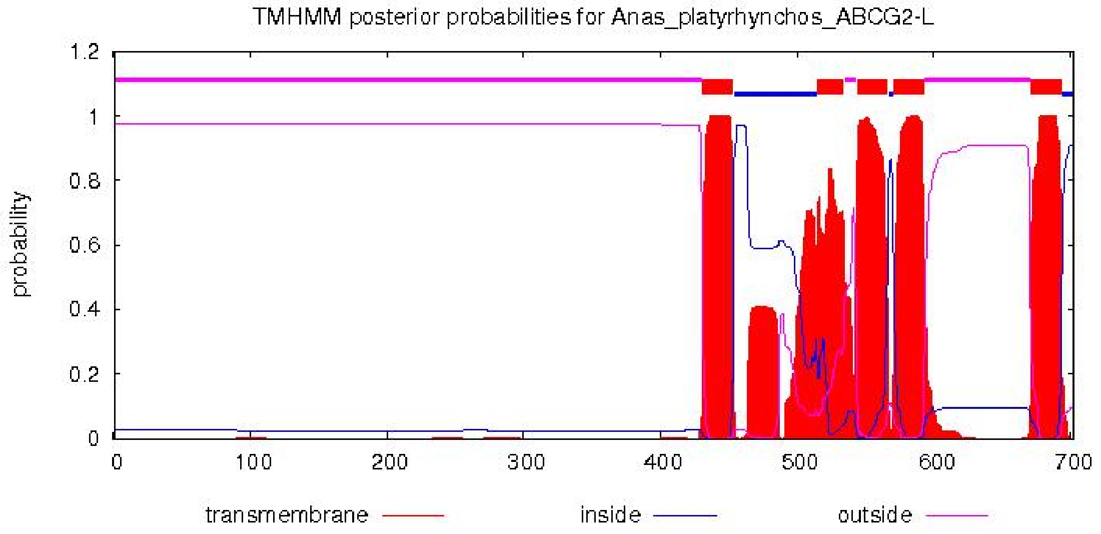
TMHMM posterior probabilities for Anas_platyrhynchos_ABCG2-L
probability
1.2
1
0.8
0.6
0.4
0.2
0
0
100
200
300
400
500
600
700
transmembrane
inside
outside

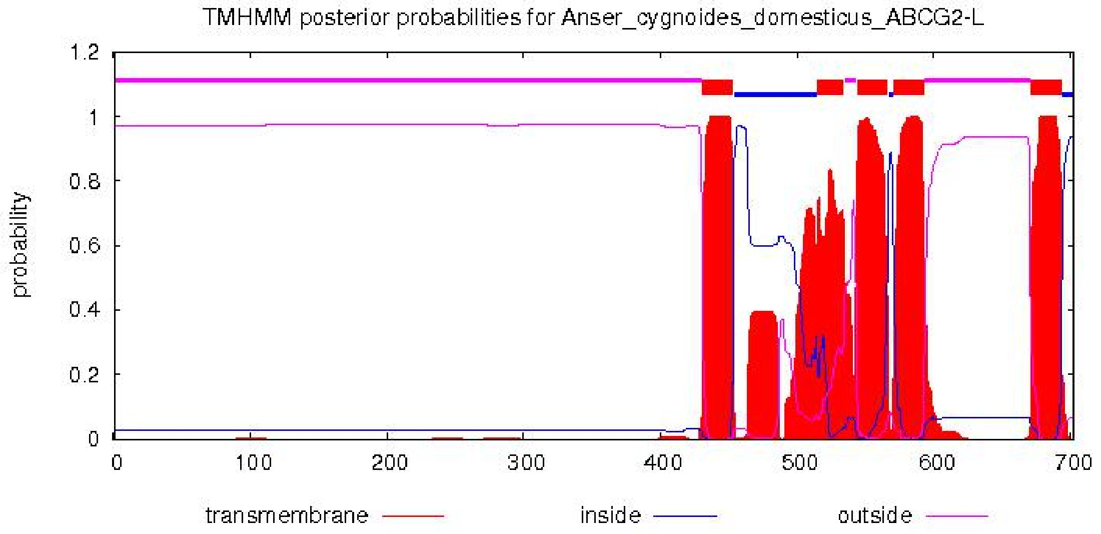
TMHMM posterior probabilities for Anser_cygnoides_domesticus_ABCG2-L
probability
1.2
1
0.8
0.6
0.4
0.2
0
0
100
200
300
400
500
600
700
transmembrane
inside
outside

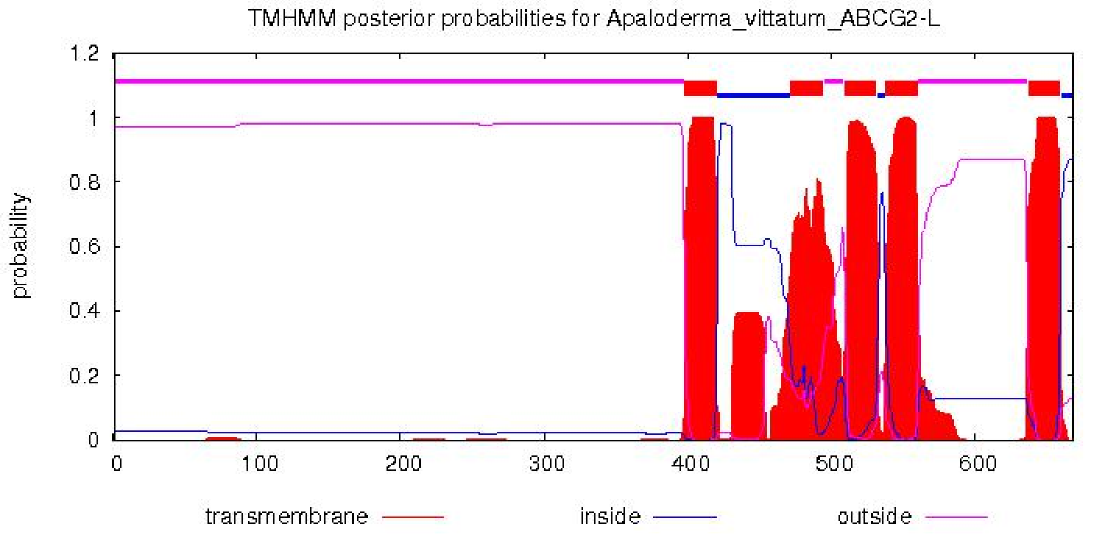
TMHMM posterior probabilities for Apaloderma_vittatum_ABCG2-L
probability
1.2
1
0.8
0.6
0.4
0.2
0
0
100
200
300
400
500
600
transmembrane
inside
outside

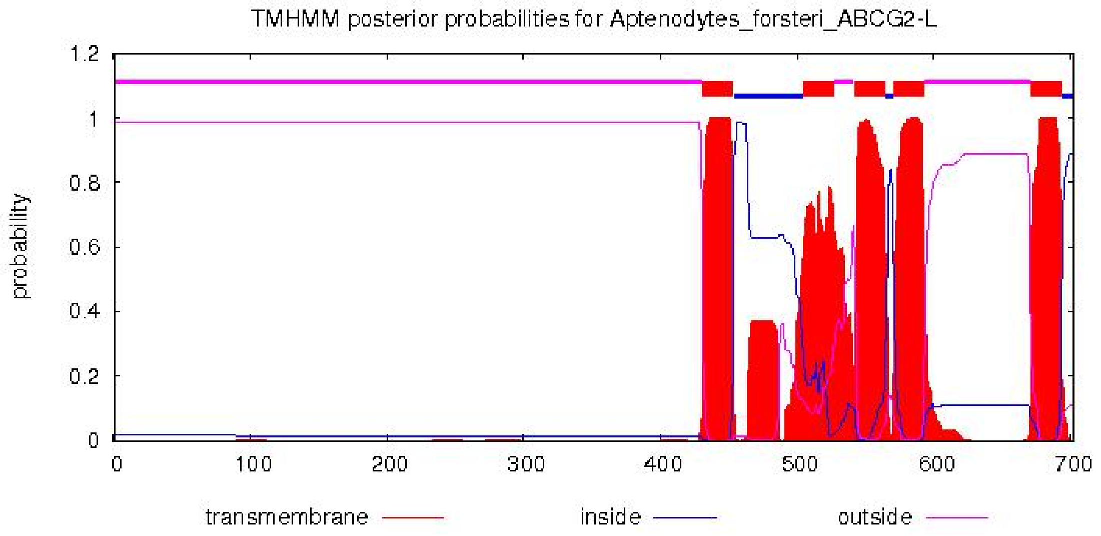
TMHMM posterior probabilities for Aptenodytes_forsteri_ABCG2-L
probability
1.2
1
0.8
0.6
0.4
0.2
0
0
100
200
300
400
500
600
700
transmembrane
inside
outside

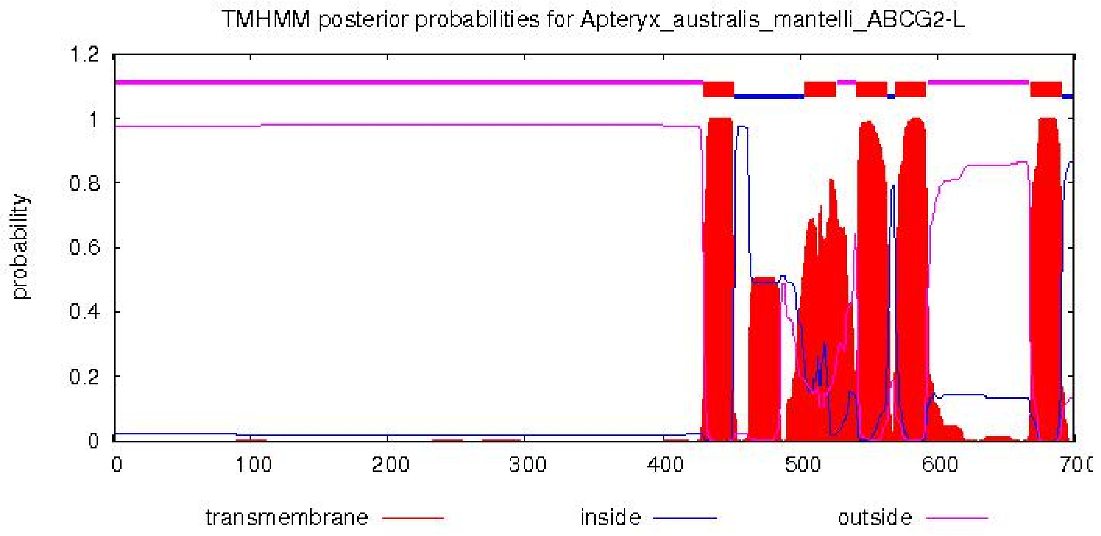
TMHMM posterior probabilities for Apteryx_australis_mantelli_ABCG2-L
probability
1.2
1
0.8
0.6
0.4
0.2
0
0
100
200
300
400
500
600
transmembrane
inside
outside

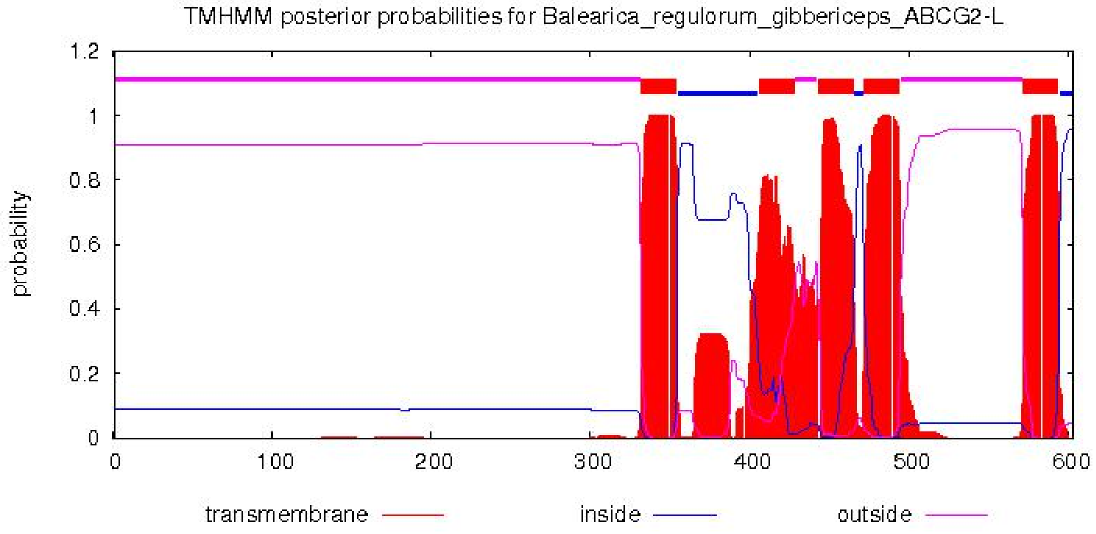
TMHMM posterior probabilities for Balearica_regulorum_gibbericeps_ABCG2-L
probability
1.2
1
0.8
0.6
0.4
0.2
0
0
100
200
300
400
500
600
transmembrane
inside
outside

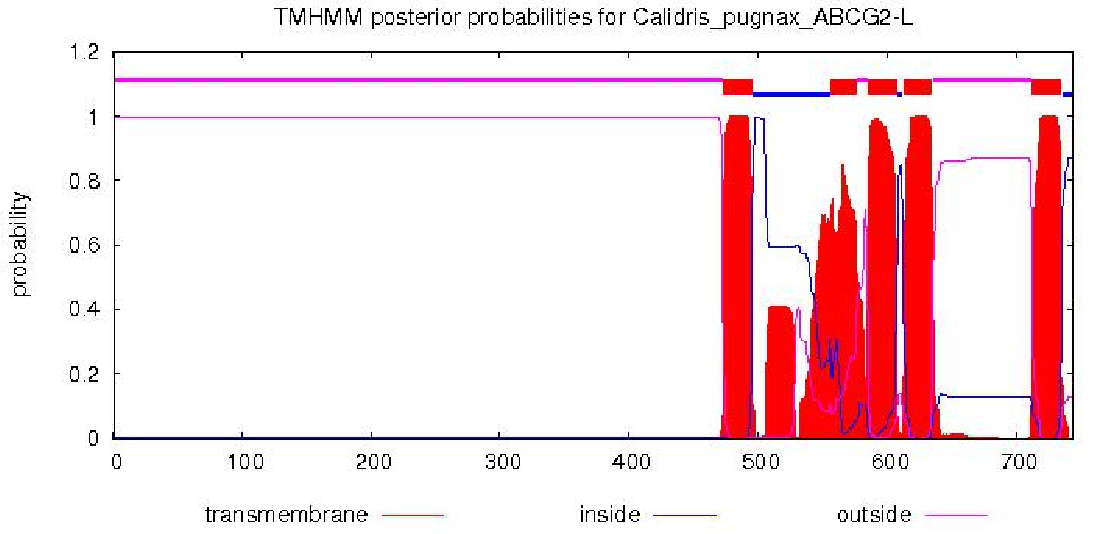
TMHMM posterior probabilities for Calidris_pugnax_ABCG2-L
probability
1.2
1
0.8
0.6
0.4
0.2
0
0
100
200
300
400
500
600
700
transmembrane
inside
outside

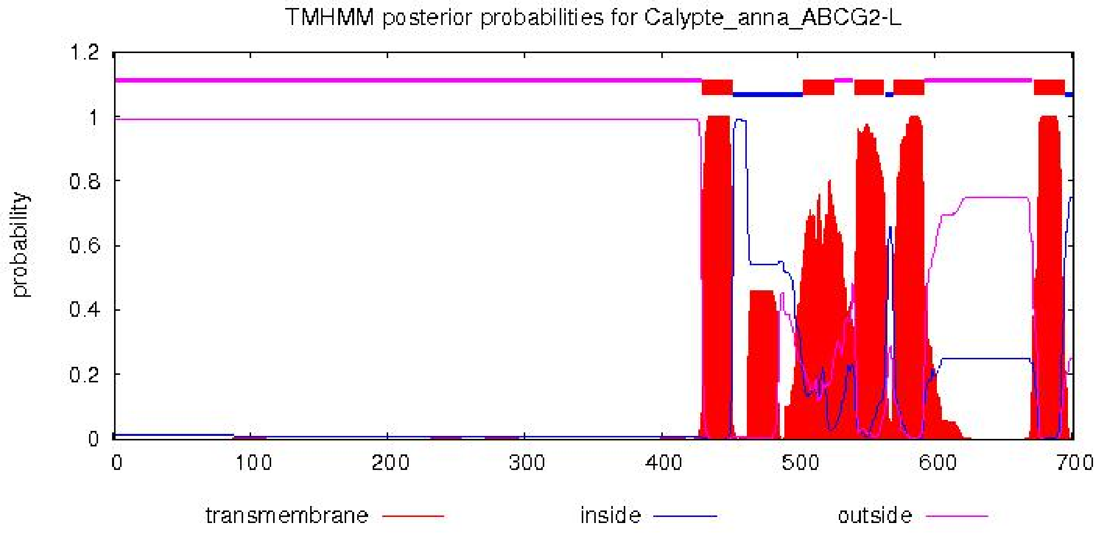
TMHMM posterior probabilities for Calypte_anna_ABCG2-L
probability
1.2
1
0.8
0.6
0.4
0.2
0
0
100
200
300
400
500
600
700
transmembrane
inside
outside

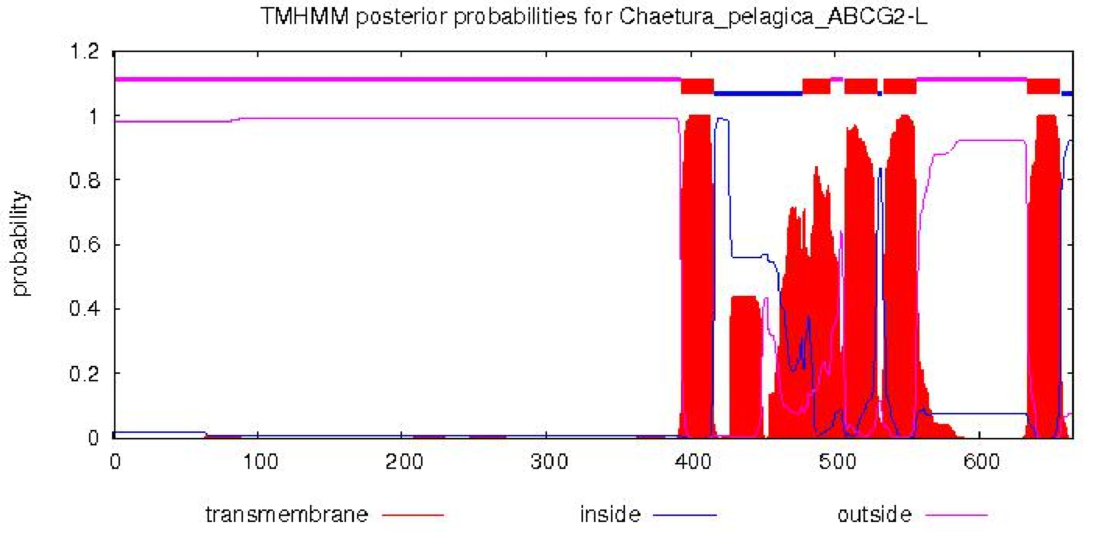
TMHMM posterior probabilities for Chaetura_pelagica_ABCG2-L
probability
transmembrane
inside
outside

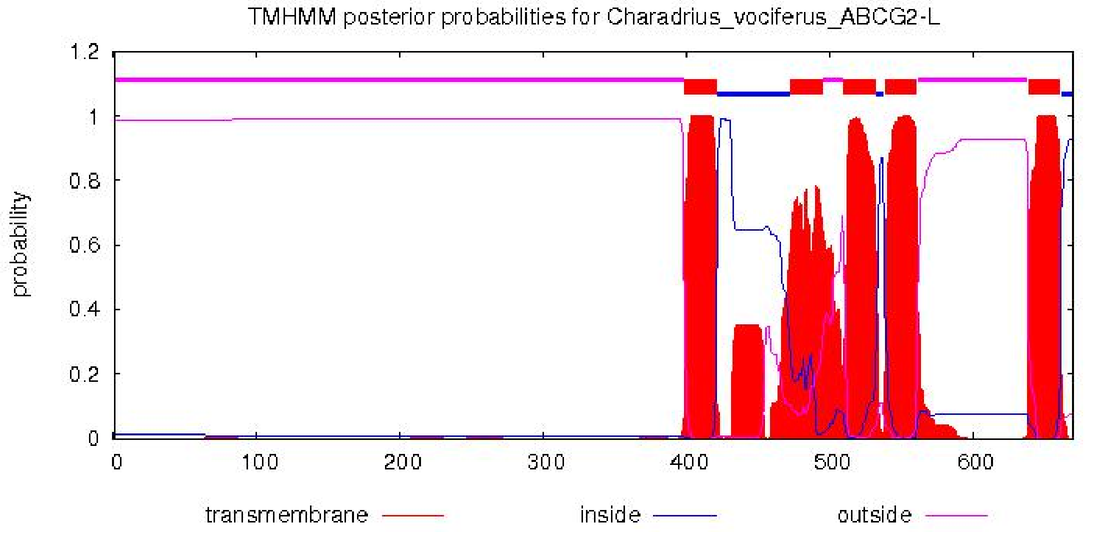
TMHMM posterior probabilities for Charadrius_vociferus_ABCG2-L
probability
transmembrane
inside
outside

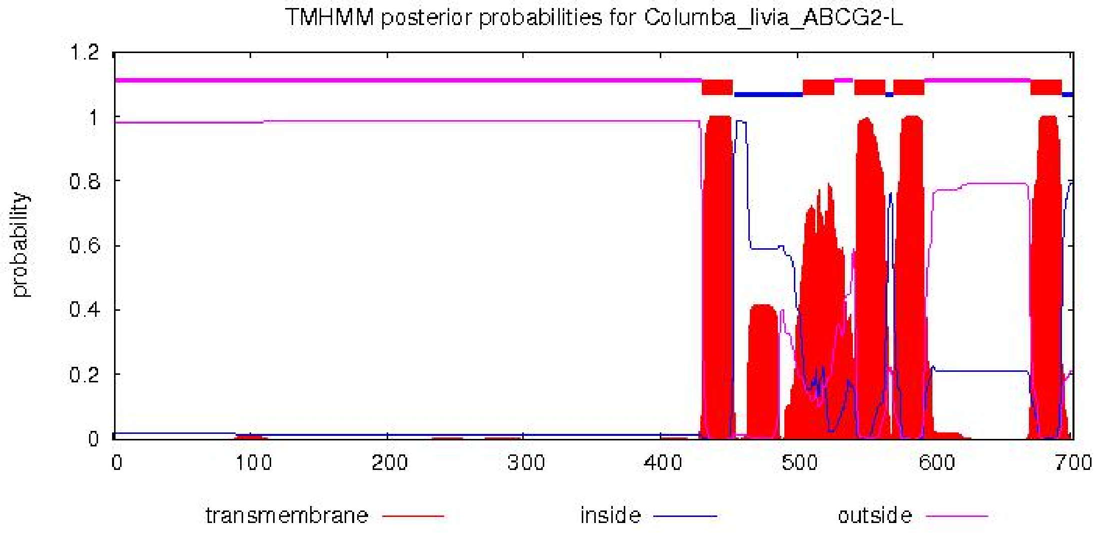
TMHMM posterior probabilities for Columba_livia_ABCG2-L
probability
transmembrane
inside
outside

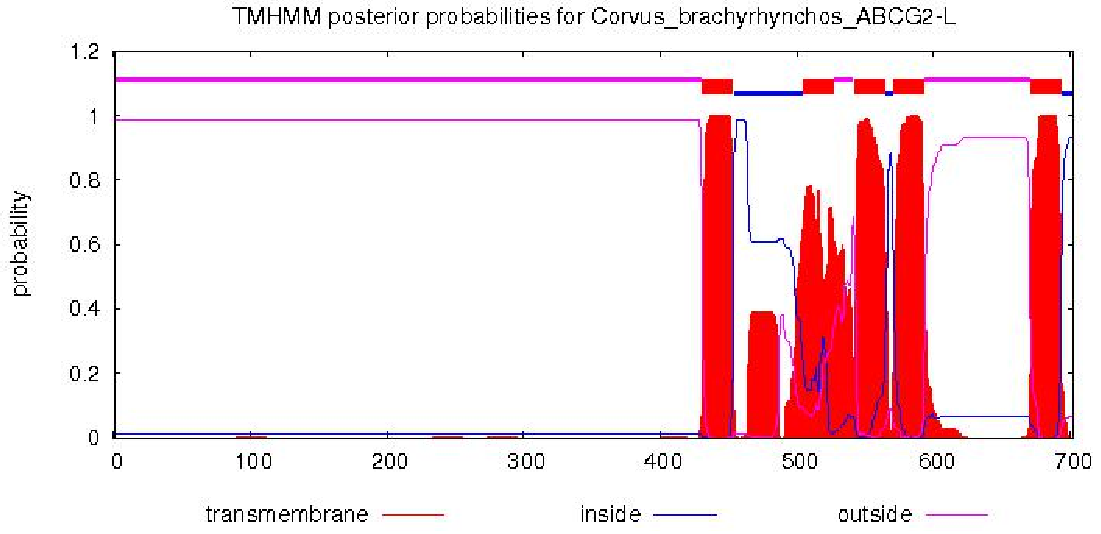
TMHMM posterior probabilities for Corvus_brachyrhynchos_ABCG2-L
probability
1.2
1
0.8
0.6
0.4
0.2
0
0
100
200
300
400
500
600
700
transmembrane
inside
outside

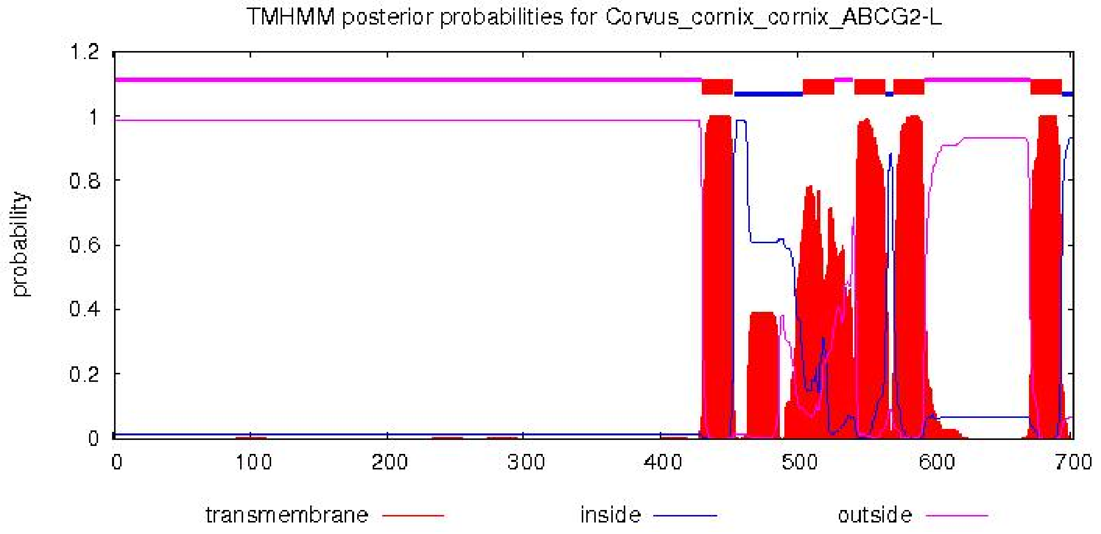
TMHMM posterior probabilities for Corvus_cornix_cornix_ABCG2-L
probability
1.2
1
0.8
0.6
0.4
0.2
0
0
100
200
300
400
500
600
700
transmembrane
inside
outside

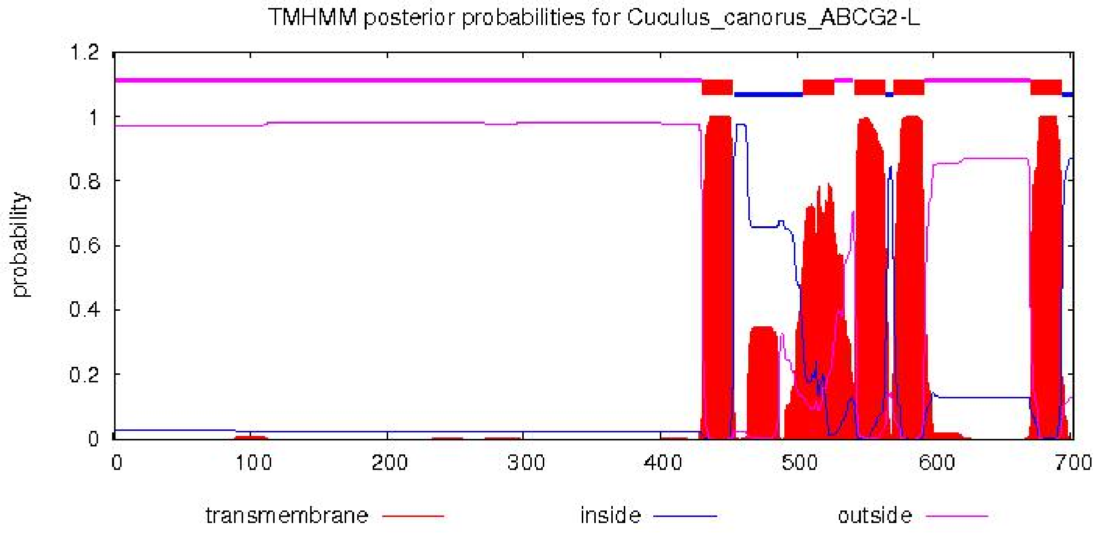
TMHMM posterior probabilities for Cuculus_canorus_ABCG2-L
probability
1.2
1
0.8
0.6
0.4
0.2
0
0
100
200
300
400
500
600
700
transmembrane
inside
outside

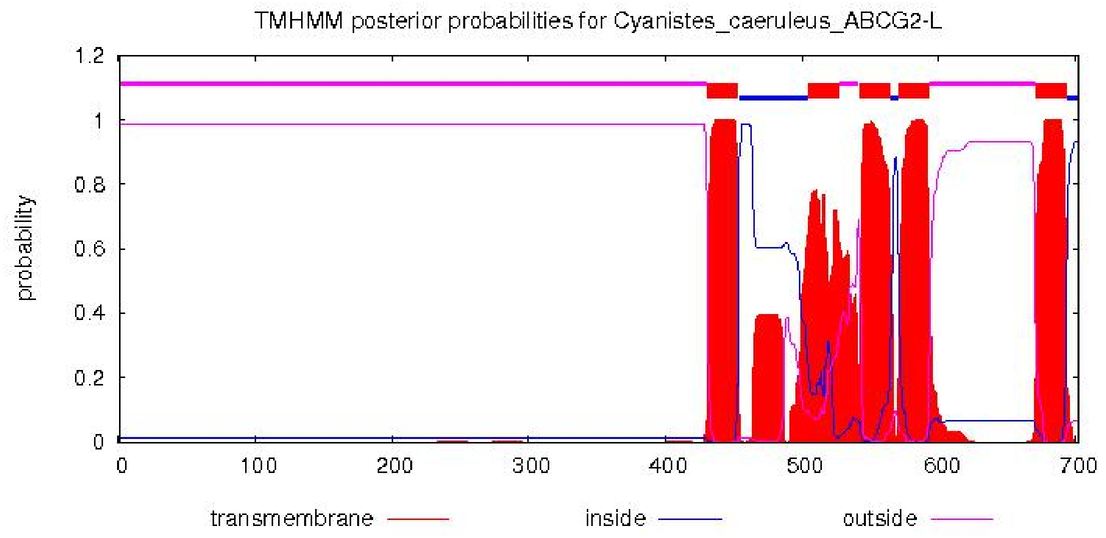
TMHMM posterior probabilities for Cyanistes_caeruleus_ABCG2-L
probability
1.2
1
0.8
0.6
0.4
0.2
0
0
100
200
300
400
500
600
700
transmembrane
inside
outside

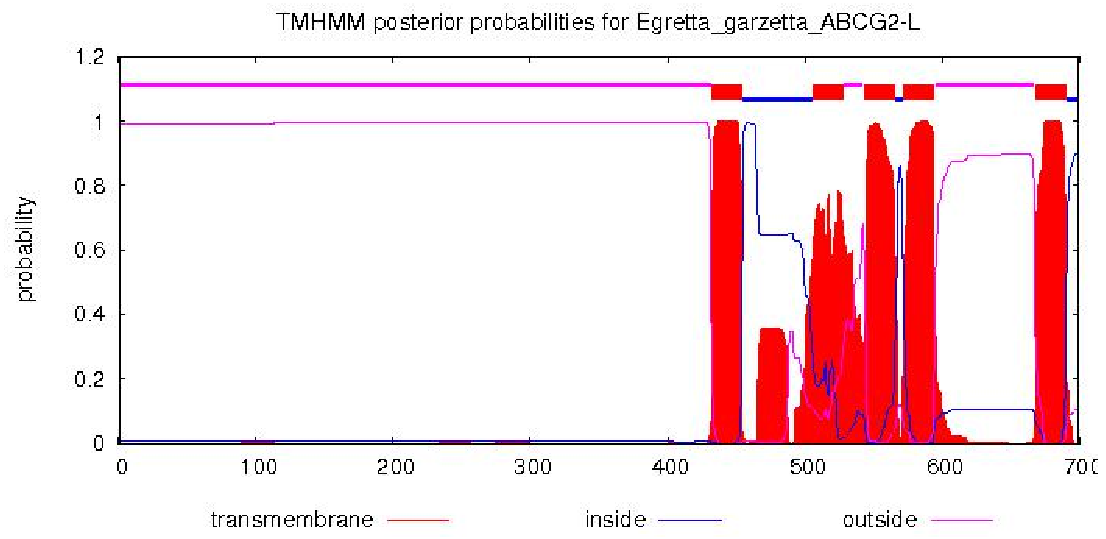
TMHMM posterior probabilities for Egretta_garzetta_ABCG2-L
probability
1.2
1
0.8
0.6
0.4
0.2
0
0
100
200
300
400
500
600
70(
transmembrane
inside
outside

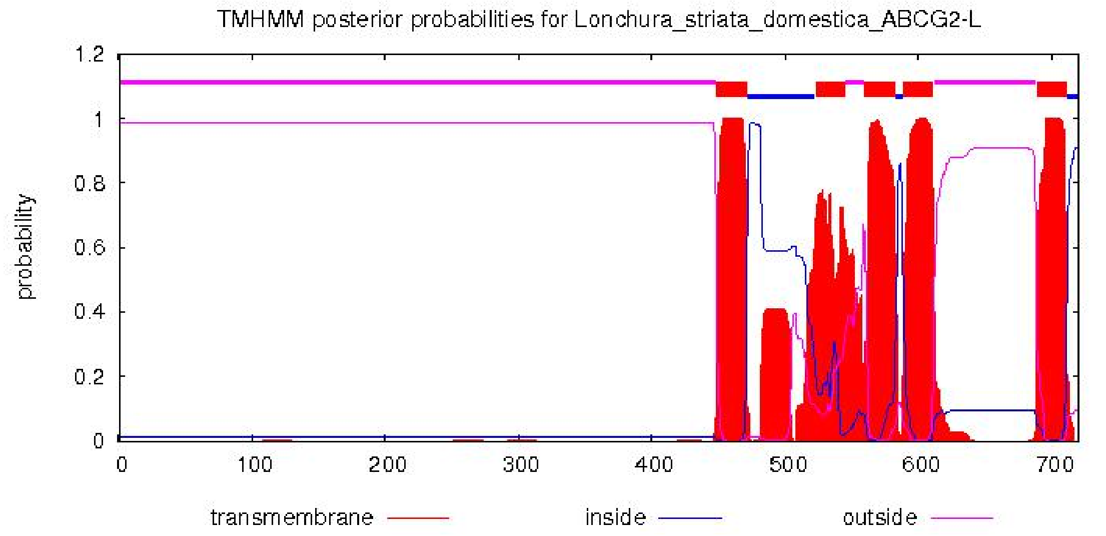
TMHMM posterior probabilities for Lonchura_striata_domestica_ABCG2-L
probability
1.2
1
0.8
0.6
0.4
0.2
0
0
100
200
300
400
500
600
700
transmembrane
inside
outside

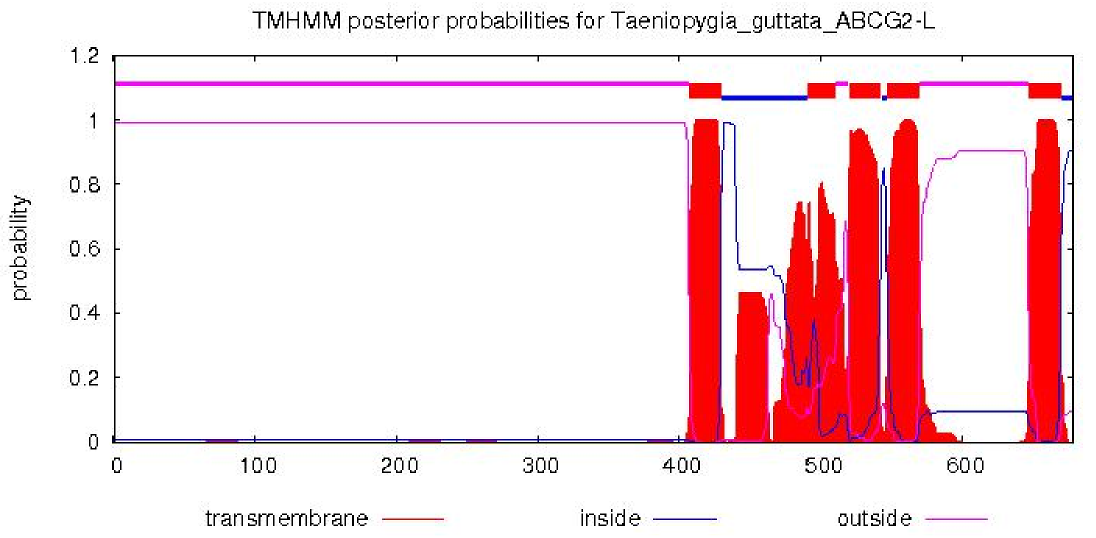
TMHMM posterior probabilities for Taeniopygia_guttata_ABCG2-L
probability
1.2
1
0.8
0.6
0.4
0.2
0
0
100
200
300
400
500
600
transmembrane
inside
outside

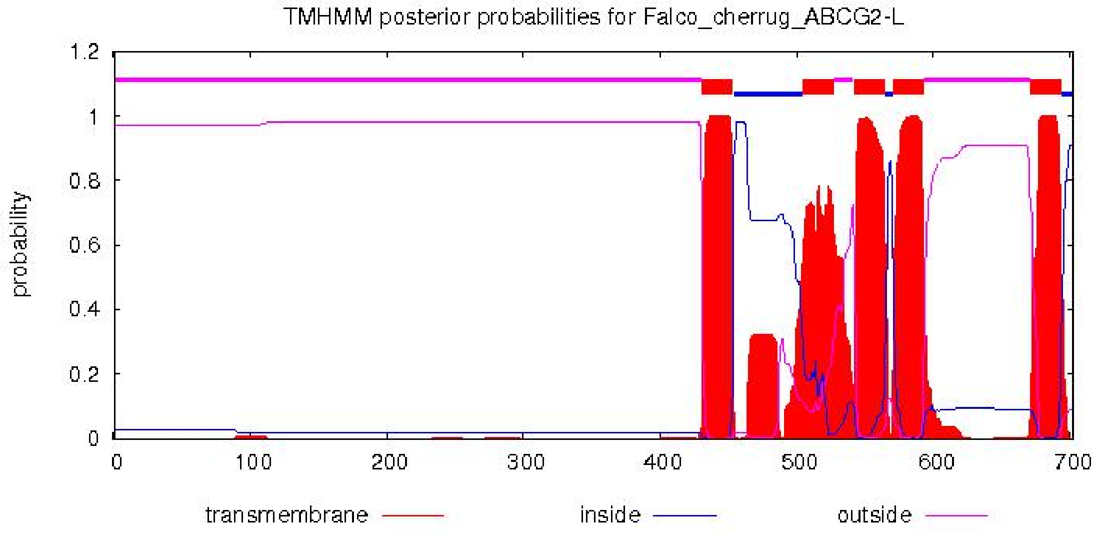
TMHMM posterior probabilities for Falco_cherrug_ABCG2-L
probability
1.2
1
0.8
0.6
0.4
0.2
0
0
100
200
300
400
500
600
700
transmembrane
inside
outside

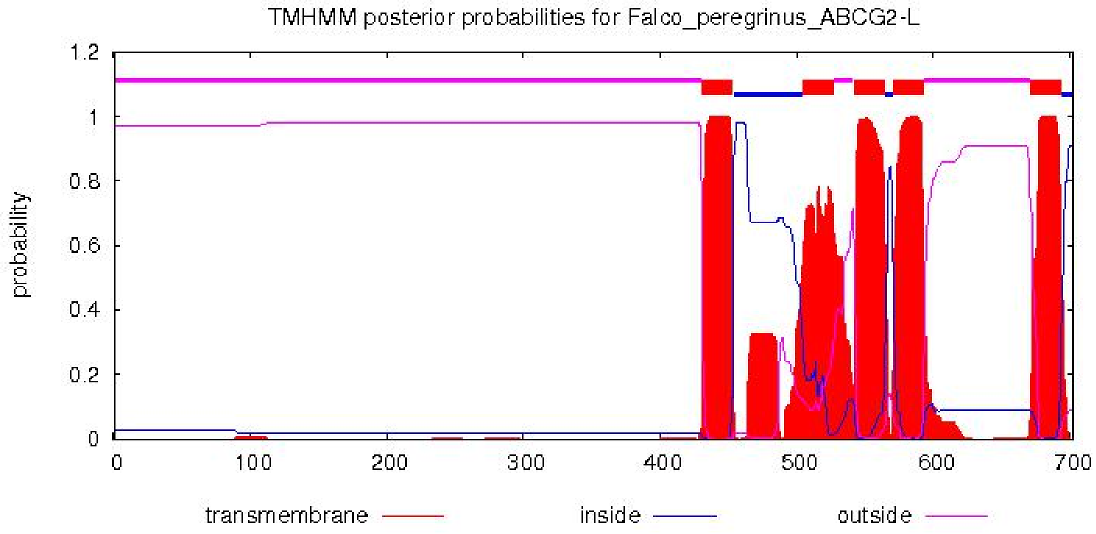
TMHMM posterior probabilities for Falco_peregrinus_ABCG2-L
probability
1.2
1
0.8
0.6
0.4
0.2
0
0
100
200
300
400
500
600
700
transmembrane
inside
outside

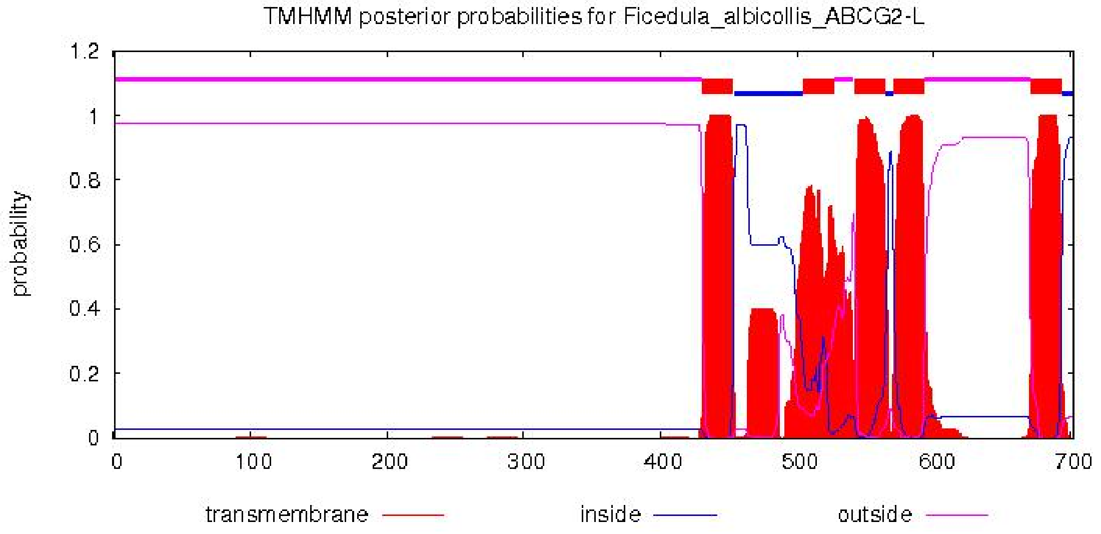

TMHMM posterior probabilities for Ficedula_albicollis_ABCG2-L
probability
1.2
1
0.8
0.6
0.4
0.2
0
0
100
200
300
400
500
600
700
transmembrane
inside
outside

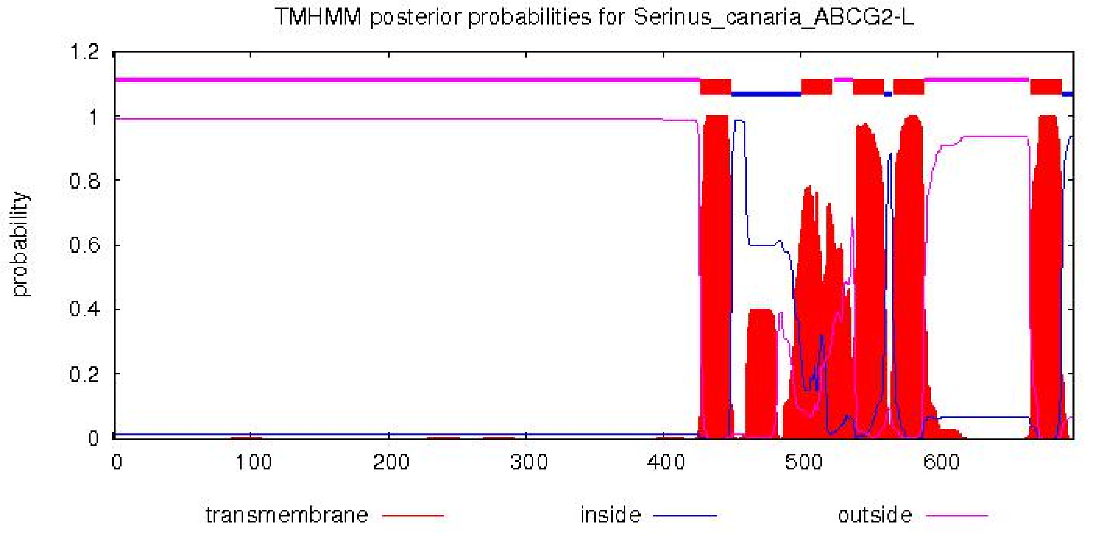

TMHMM posterior probabilities for Serinus_canaria_ABCG2-L
probability
1.2
1
0.8
0.6
0.4
0.2
0
0
100
200
300
400
500
600
transmembrane
inside
outside

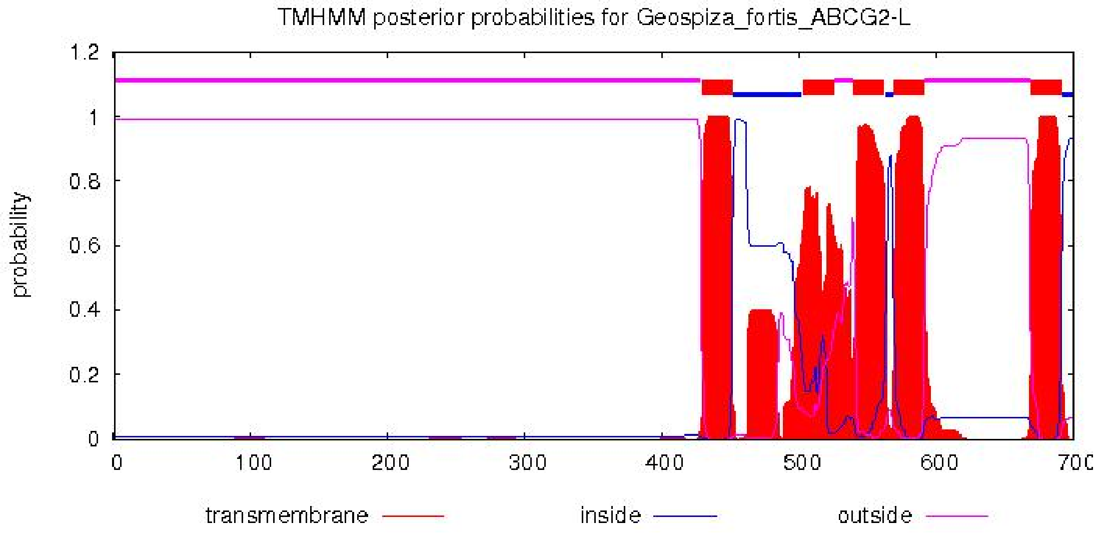

TMHMM posterior probabilities for Geospiza_fortis_ABCG2-L
probability
1.2
1
0.8
0.6
0.4
0.2
0
0
100
200
300
400
500
600
transmembrane
inside
outside

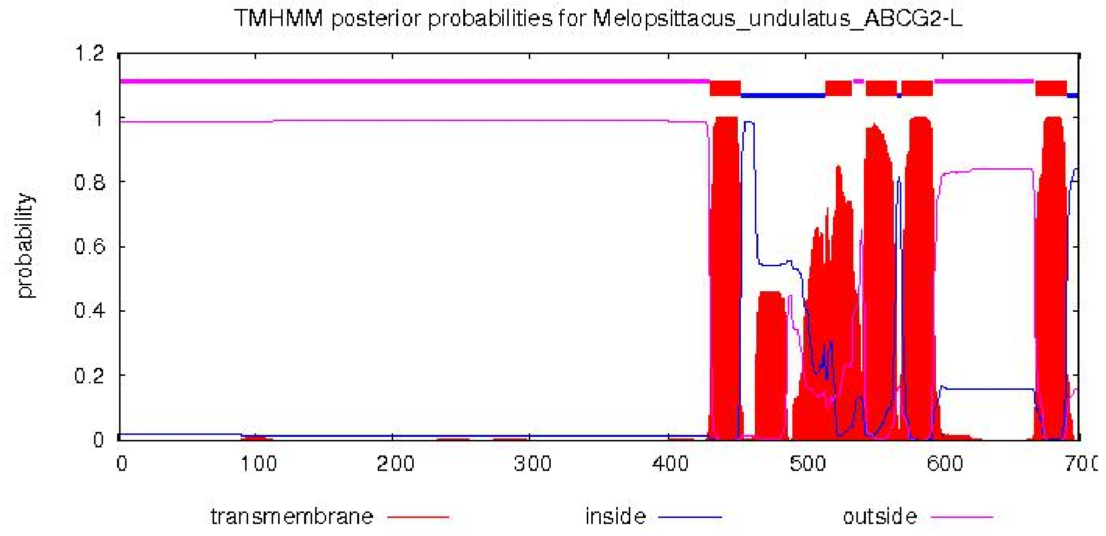
TMHMM posterior probabilities for Melopsittacus_undulatus_ABCG2-L
probability
1.2
1
0.8
0.6
0.4
0.2
0
0
100
200
300
400
500
600
700
transmembrane
inside
outside

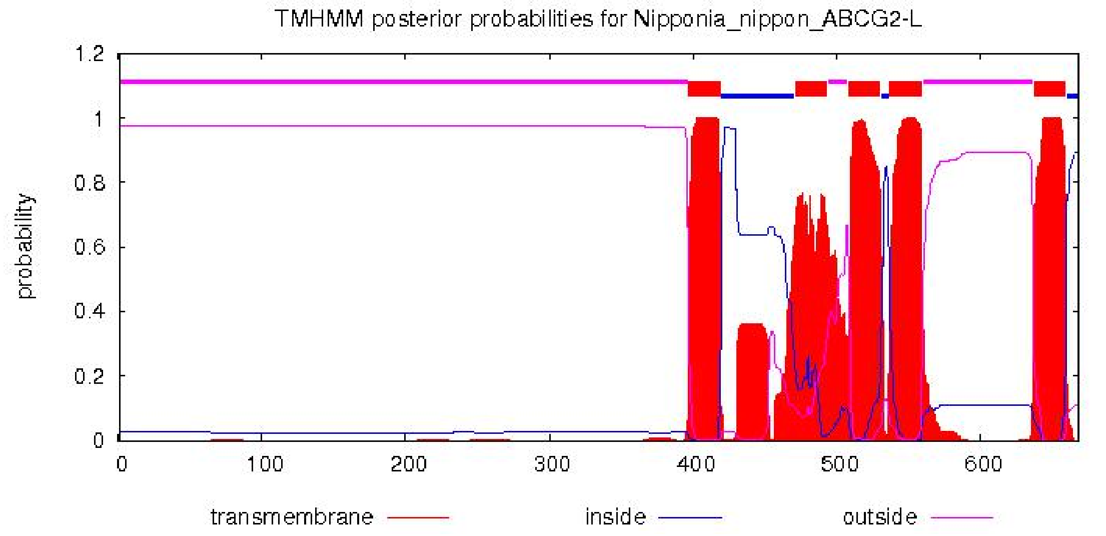
TMHMM posterior probabilities for Nipponia_nippon_ABCG2-L
probability
1.2
1
0.8
0.6
0.4
0.2
0
0
100
200
300
400
500
600
transmembrane
inside
outside

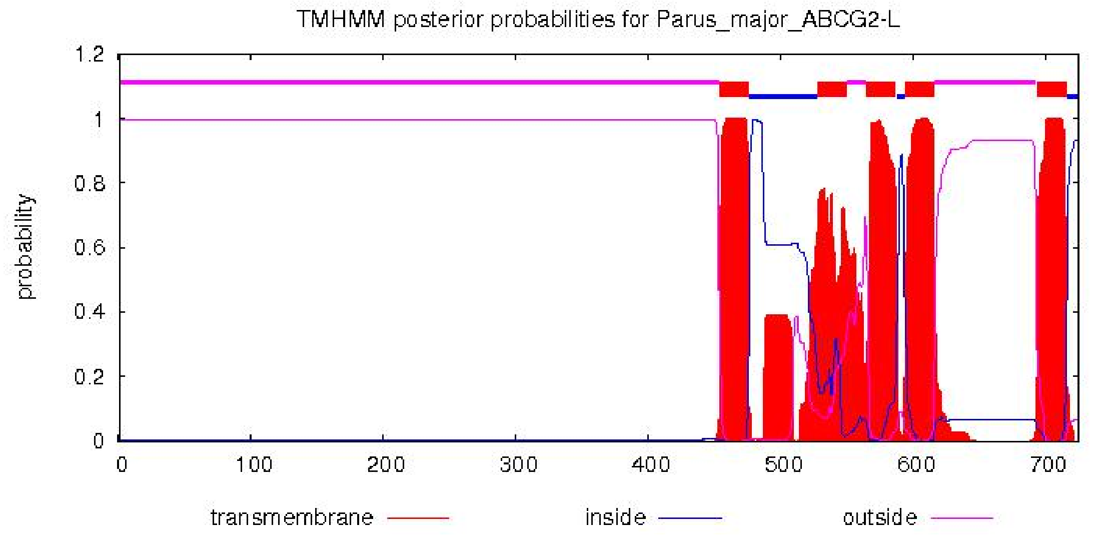
TMHMM posterior probabilities for Parus_major_ABCG2-L
probability
1.2
1
0.8
0.6
0.4
0.2
0
0
100
200
300
400
500
600
700
transmembrane
inside
outside

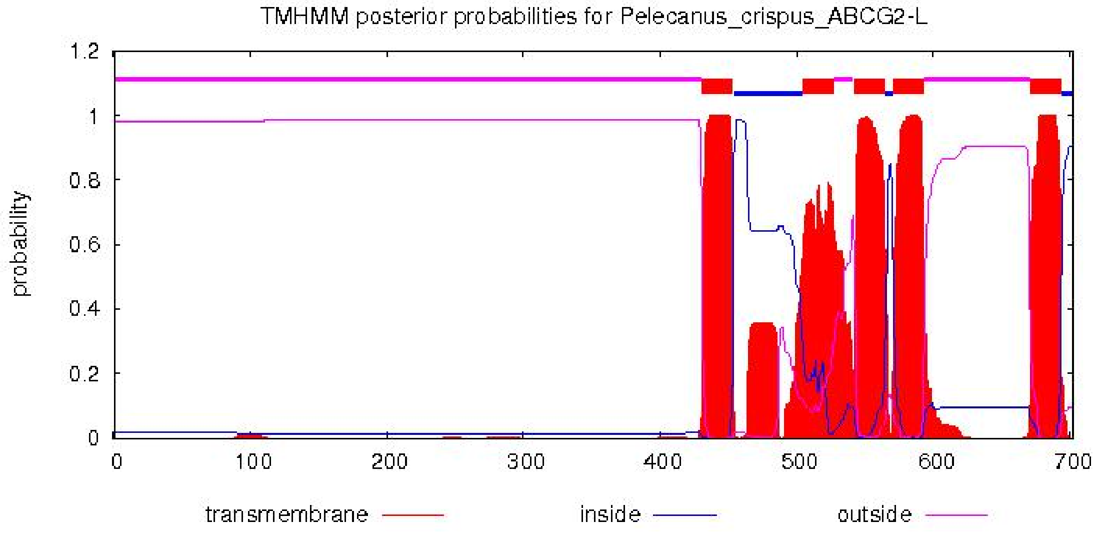
TMHMM posterior probabilities for Pelecanus_crispus_ABCG2-L
probability
1.2
1
0.8
0.6
0.4
0.2
0
0
100
200
300
400
500
600
700
transmembrane
inside
outside

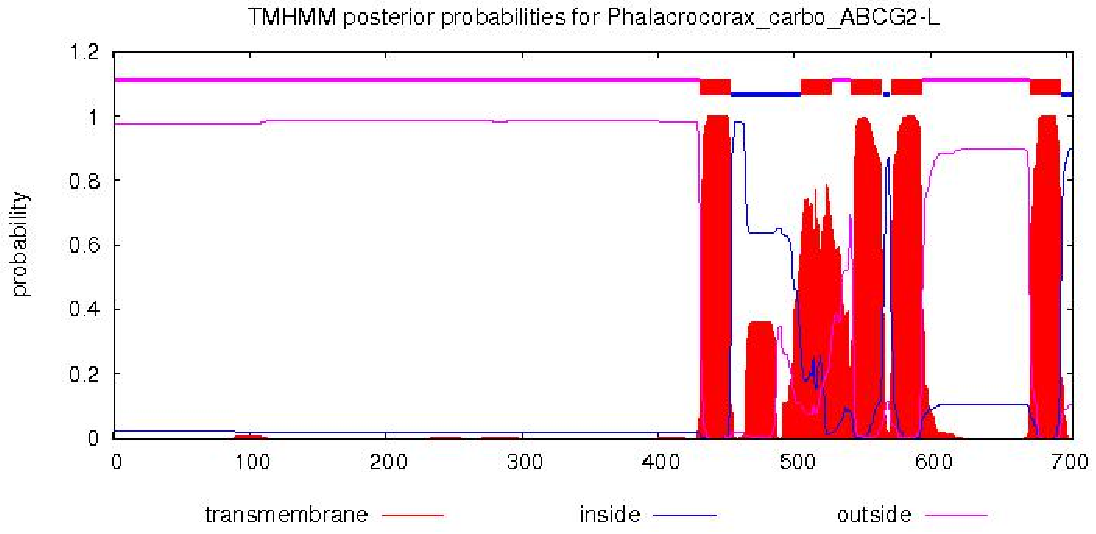
TMHMM posterior probabilities for Phalacrocorax_carbo_ABCG2-L
probability
1.2
1
0.8
0.6
0.4
0.2
0
0
100
200
300
400
500
600
700
transmembrane
inside
outside

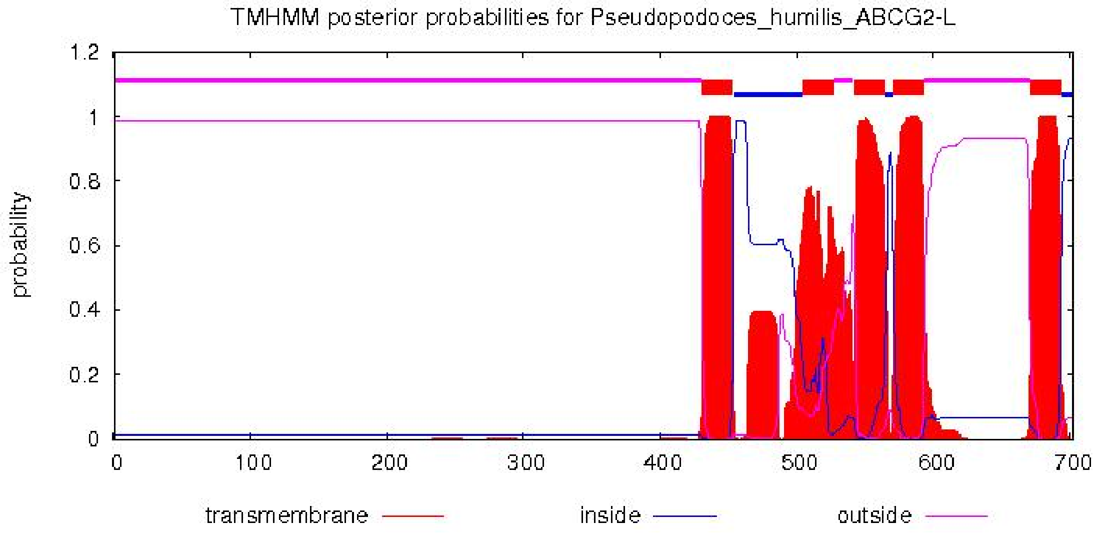
TMHMM posterior probabilities for Pseudopodoces_humilis_ABCG2-L
probability
1.2
1
0.8
0.6
0.4
0.2
0
0
100
200
300
400
500
600
700
transmembrane
inside
outside

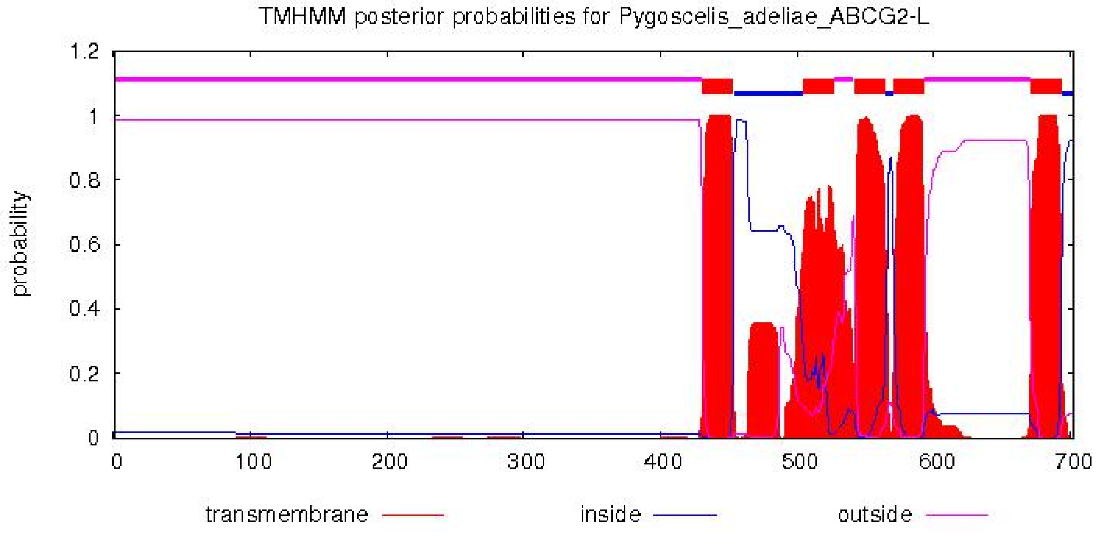

TMHMM posterior probabilities for Pygoscelis_adeliae_ABCG2-L
probability
transmembrane
inside
outside

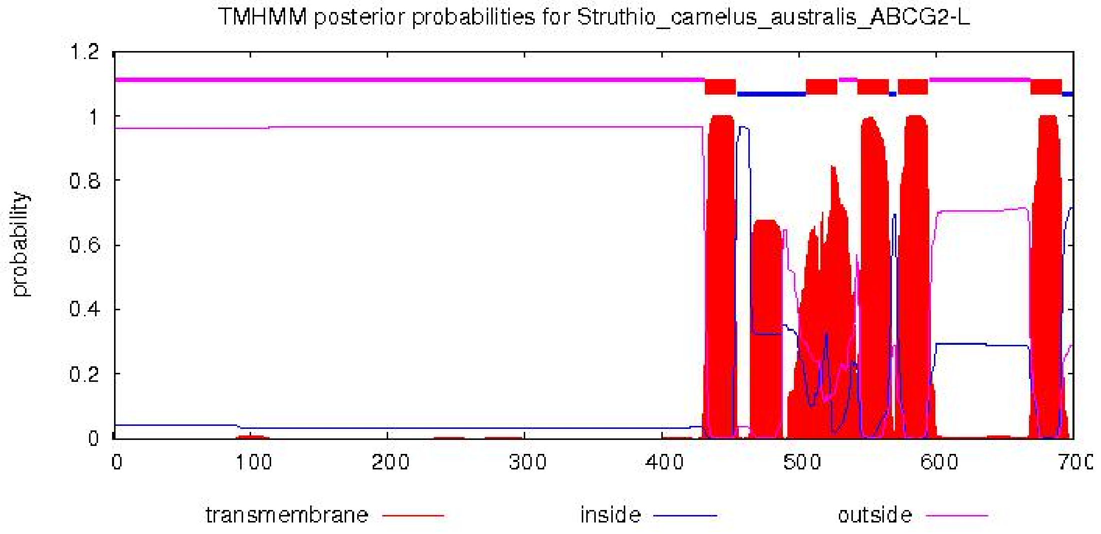

TMHMM posterior probabilities for Struthio_camelus_australis_ABCG2-L
probability
transmembrane
inside
outside

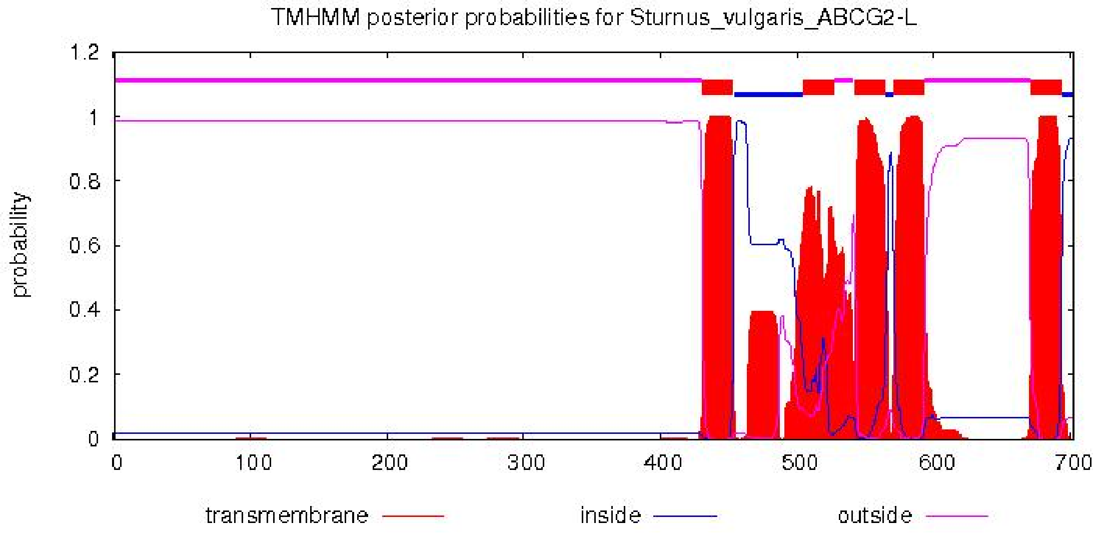

TMHMM posterior probabilities for Sturnus_vulgaris_ABCG2-L
probability
transmembrane
inside
outside

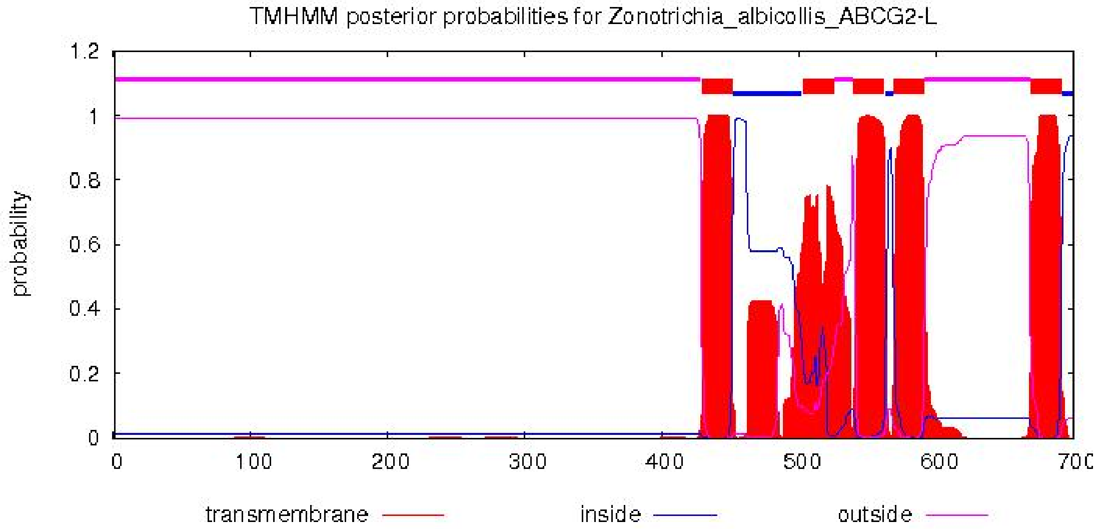

Outgroups species *ABCG2* gene subfamily members

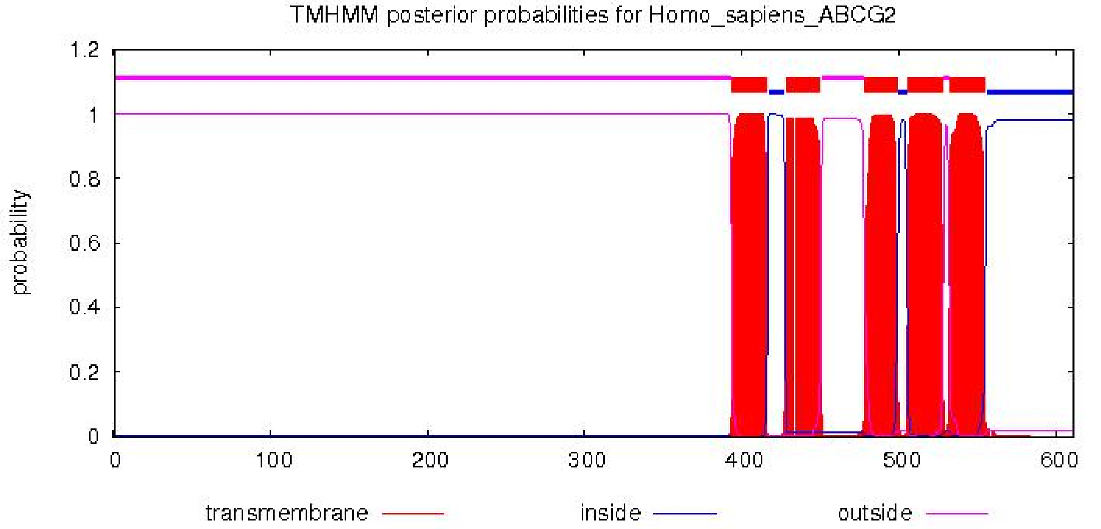

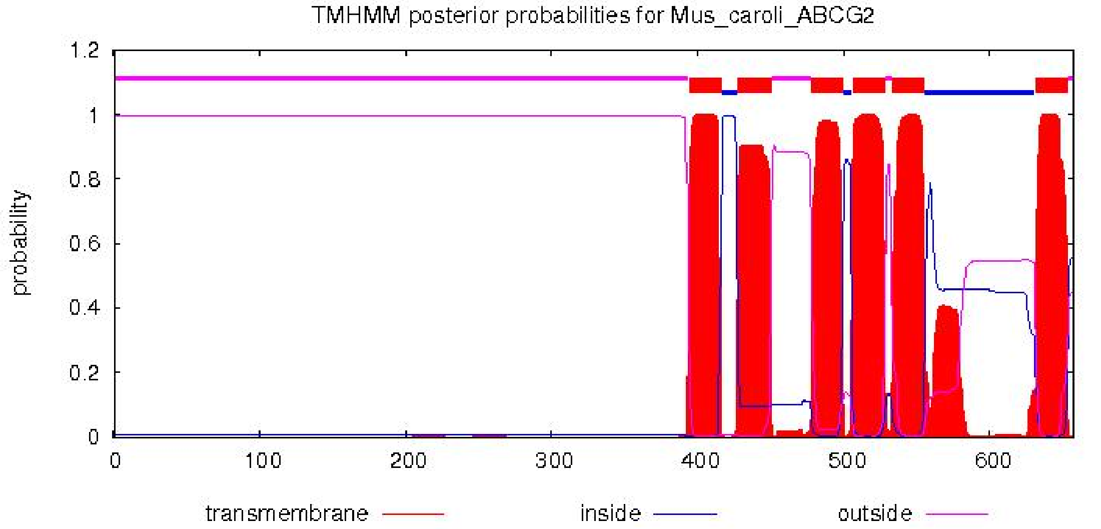

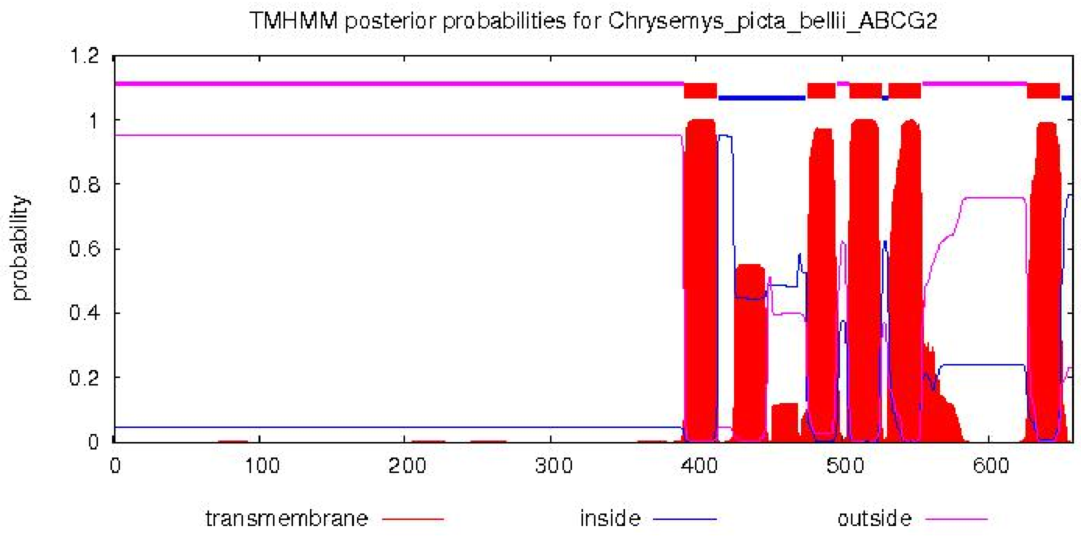
TMHMM posterior probabilities for Chrysemys_picta_bellii_ABCG2
probability
1.2
1
0.8
0.6
0.4
0.2
0
0
100
200
300
400
500
600
transmembrane
inside
outside

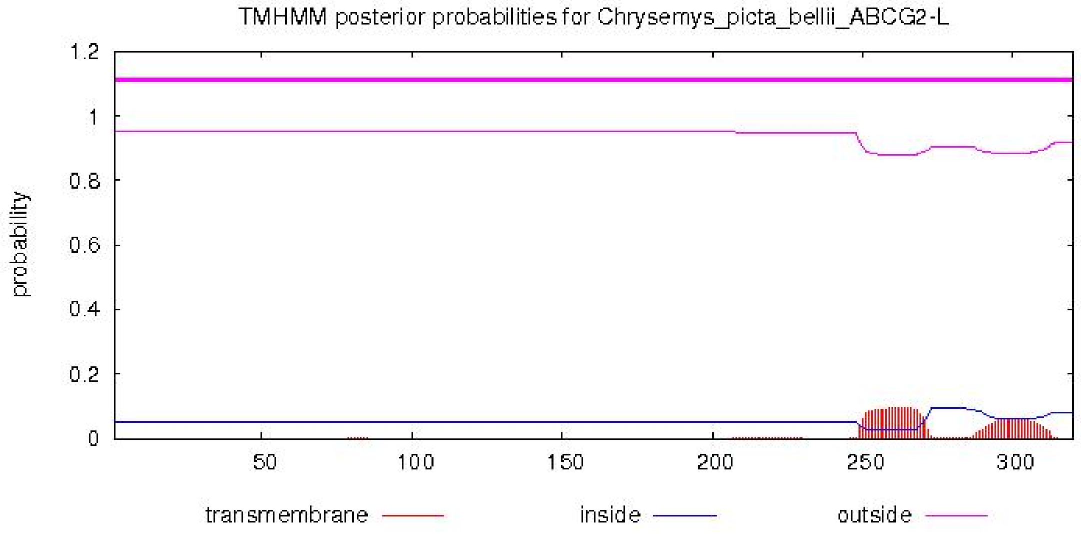
TMHMM posterior probabilities for Chrysemys_picta_bellii_ABCG2-L
probability
1.2
1
0.8
0.6
0.4
0.2
0
50
100
150
200
250
300
transmembrane
inside
outside

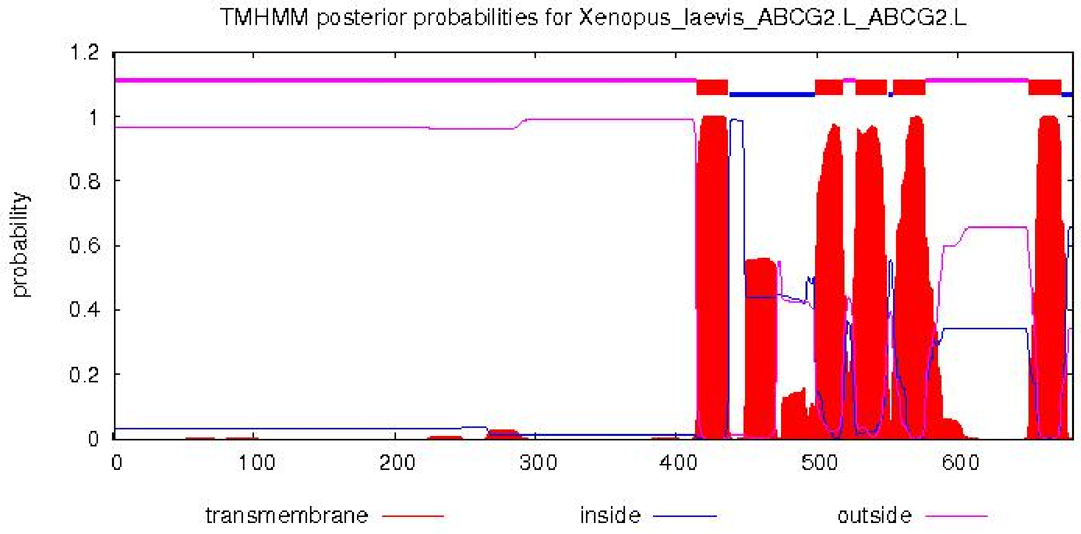
TMHMM posterior probabilities for Xenopus_laevis_ABCG2.L_ABCG2.L
probability
1.2
1
0.8
0.6
0.4
0.2
0
0
100
200
300
400
500
600
transmembrane
inside
outside

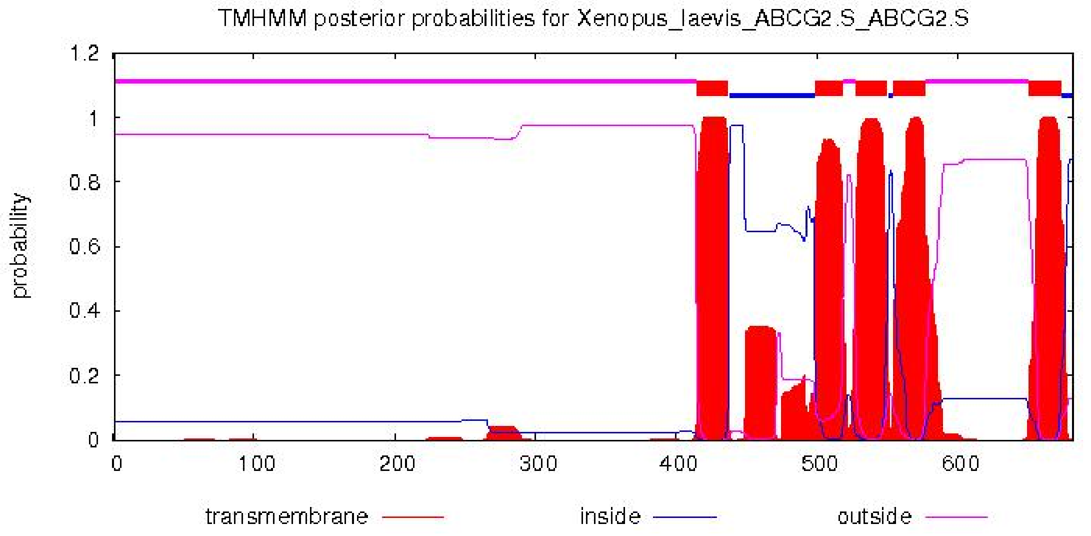
TMHMM posterior probabilities for Xenopus_laevis_ABCG2.S_ABCG2.S
probability
1.2
1
0.8
0.6
0.4
0.2
0
0
100
200
300
400
500
600
transmembrane
inside
outside

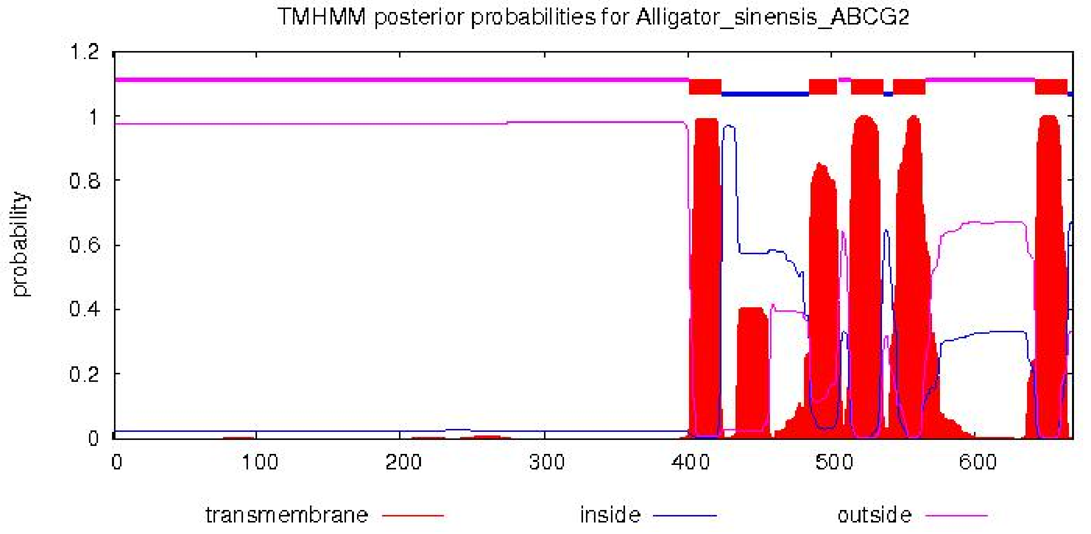
TMHMM posterior probabilities for Alligator_sinensis_ABCG2
probability
1.2
1
0.8
0.6
0.4
0.2
0
0
100
200
300
400
500
600
transmembrane
inside
outside

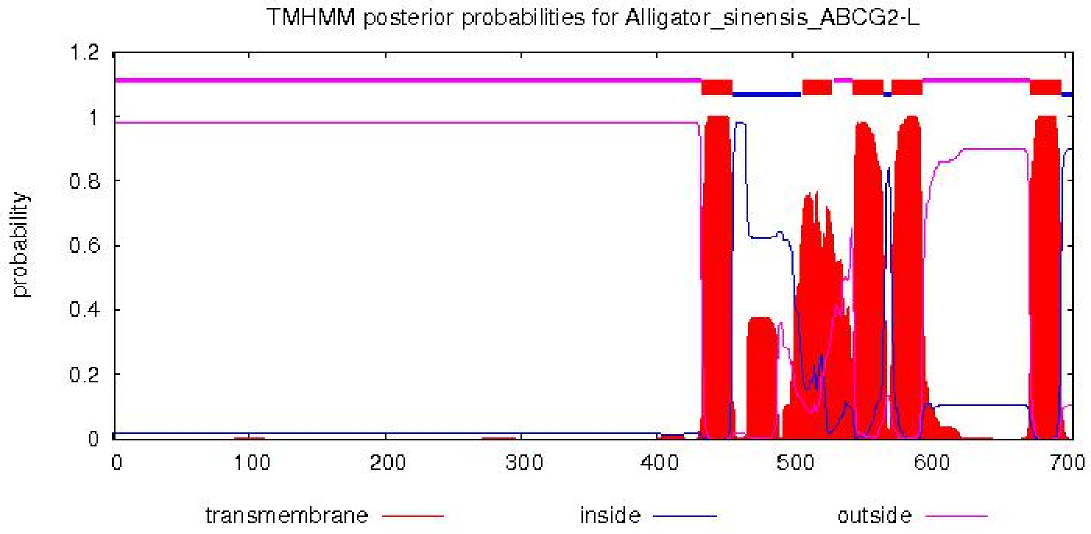
TMHMM posterior probabilities for Alligator_sinensis_ABCG2-L
probability
1.2
1
0.8
0.6
0.4
0.2
0
0
100
200
300
400
500
600
700
transmembrane
inside
outside
