# Supplementary material for: Molecular evolution of the ATP-binding cassette subfamily G member 2 gene subfamily and its paralogs in birds
Source: BMC Evol Biol. 2020 Jul 14;20:85. doi: 10.1186/s12862-020-01654-z (PMC7362505; doi:10.1186/s12862-020-01654-z)
Supplement: Supplementary file 7 — Additional file 7: Table S7. Number of phosphorylation sites in the ABCG2 and ABCG2-like amino acid sequences of birds. [file 12862_2020_1654_MOESM7_ESM.docx]

**Table 8 Number of phosphorylation sites in the ABCG2 and ABCG2-like amino acid sequences of birds.**

| **Avian species** | **ABCG2 Phosphorylation site / Amino acid residue** | | | **ABCG2-like Phosphorylation site / Amino acid residue** | | |
| --- | --- | --- | --- | --- | --- | --- |
|  | **Serine(S)** | **Threonine(T)** | **Tyrosine(Y)** | **Serine(S)** | **Threonine(T)** | **Tyrosine(Y)** |
| ***Anas platyrhynchos*** | **8/53** | **1/54** | **4/23** | **7/64** | **5/38** | **3/23** |
| ***Anser cygnoides domesticus*** | **5/65** | **1/58** | **2/22** | **5/61** | **6/38** | **3/23** |
| ***Apaloderma vittatum*** | **4/53** | **3/50** | **3/21** | **7/61** | **5/38** | **3/23** |
| ***Apteryx australis mantelli*** | **4/55** | **2/44** | **7/23** | **6/59** | **5/39** | **2/23** |
| ***Aquila chrysaetos canadensis*** | **3/51** | **2/46** | **4/21** | **5/61** | **4/37** | **3/23** |
| ***Balearica regulorum gibbericeps*** | **6/55** | **2/50** | **2/22** | **5/55** | **3/32** | **3/23** |
| ***Calidris pugnax*** | **5/56** | **3/50** | **5/24** | **8/64** | **5/39** | **4/25** |
| ***Chaetura pelagica*** | **6/55** | **5/46** | **4/21** | **6/61** | **4/34** | **3/23** |
| ***Calypte anna*** | **7/55** | **2/51** | **3/22** | **9/68** | **5/35** | **3/24** |
| ***Charadrius vociferus*** | **5/49** | **3/50** | **4/22** | **6/61** | **4/34** | **3/23** |
| ***Columba livia*** | **7/49** | **2/50** | **6/23** | **7/63** | **4/36** | **3/24** |
| ***Corvus brachyrhynchos*** | **9/64** | **2/42** | **5/27** | **6/65** | **5/37** | **4/23** |
| ***Corvus cornix cornix*** | **9/61** | **2/44** | **4/24** | **6/65** | **5/37** | **4/23** |
| ***Cuculus canorus*** | **6/52** | **3/45** | **4/21** | **7/66** | **5/37** | **3/23** |
| ***Cyanistes caeruleus*** | **6/58** | **2/46** | **4/24** | **8/65** | **3/36** | **4/23** |
| ***Egretta garzetta*** | **6/53** | **2/47** | **4/22** | **7/65** | **6/35** | **3/23** |
| ***Falco cherrug*** | **3/51** | **4/47** | **3/23** | **7/64** | **5/37** | **3/24** |
| ***Falco peregrinus*** | **3/51** | **4/47** | **3/23** | **7/64** | **5/37** | **3/24** |
| ***Ficedula albicollis*** | **9/54** | **2/43** | **2/23** | **10/66** | **4/36** | **4/23** |
| ***Geospiza fortis*** | **5/54** | **4/46** | **1/23** | **10/67** | **4/39** | **5/23** |
| ***Haliaeetus albicilla*** | **4/51** | **3/47** | **4/21** | **5/59** | **4/37** | **3/23** |
| ***Lonchura striata domestica*** | **5/58** | **2/46** | **3/25** | **8/66** | **4/41** | **3/24** |
| ***Melopsittacus undulatus*** | **7/57** | **2/48** | **3/24** | **9/66** | **6/38** | **4/23** |
| ***Manacus vitellinus*** | **8/52** | **3/50** | **5/23** |  |  |  |
| ***Mesitornis unicolor*** | **8/53** | **2/48** | **3/24** |  |  |  |
| ***Nipponia nippon*** | **5/56** | **2/47** | **5/23** | **6/61** | **6/33** | **3/23** |
| ***Parus major*** | **10/60** | **2/46** | **3/23** | **7/64** | **3/38** | **4/25** |
| ***Pelecanus crispus*** | **4/54** | **2/47** | **4/22** | **7/61** | **3/36** | **4/23** |
| ***Phalacrocorax carbo*** | **6/53** | **2/48** | **5/23** | **5/63** | **5/34** | **3/23** |
| ***Pseudopodoces humilis*** | **9/60** | **3/45** | **4/23** | **8/66** | **3/35** | **4/23** |
| ***Pygoscelis adeliae*** | **4/55** | **2/53** | **5/23** | **8/65** | **4/35** | **3/22** |
| ***Serinus canaria*** | **6/58** | **2/44** | **2/25** | **10/67** | **4/39** | **4/23** |
| ***Struthio camelus australis*** | **4/40** | **3/37** | **5/17** | **8/63** | **6/40** | **3/24** |
| ***Sturnus vulgaris*** | **8/55** | **3/44** | **4/24** | **8/65** | **3/38** | **4/23** |
| ***Zonotrichia albicollis*** | **4/65** | **5/38** | **2/23** | **6/67** | **4/40** | **3/24** |
| ***Meleagris gallopavo*** | **7/61** | **4/38** | **3/22** |  |  |  |
| ***Gallus gallus*** | **7/60** | **5/38** | **3/22** |  |  |  |
| ***Taeniopygia guttata*** | **4/56** | **2/46** | **4/25** | **9/65** | **3/37** | **4/23** |
| ***Coturnix japonica*** | **7/61** | **4/37** | **3/21** |  |  |  |
| ***Aptenodytes forsteri*** | **4/55** | **2/51** | **5/23** | **7/62** | **4/35** | **3/23** |
| ***Haliaeetus leucocephalus*** | **4/51** | **3/46** | **4/21** | **5/60** | **4/37** | **3/23** |
| **Average** | **6/55** | **3/46** | **4/23** | **7/63** | **4/37** | **3/23** |
