# Supplementary material for: Molecular evolution of the ATP-binding cassette subfamily G member 2 gene subfamily and its paralogs in birds
Source: BMC Evol Biol. 2020 Jul 14;20:85. doi: 10.1186/s12862-020-01654-z (PMC7362505; doi:10.1186/s12862-020-01654-z)

**Query *Anas platyrhynchos ABCG2***

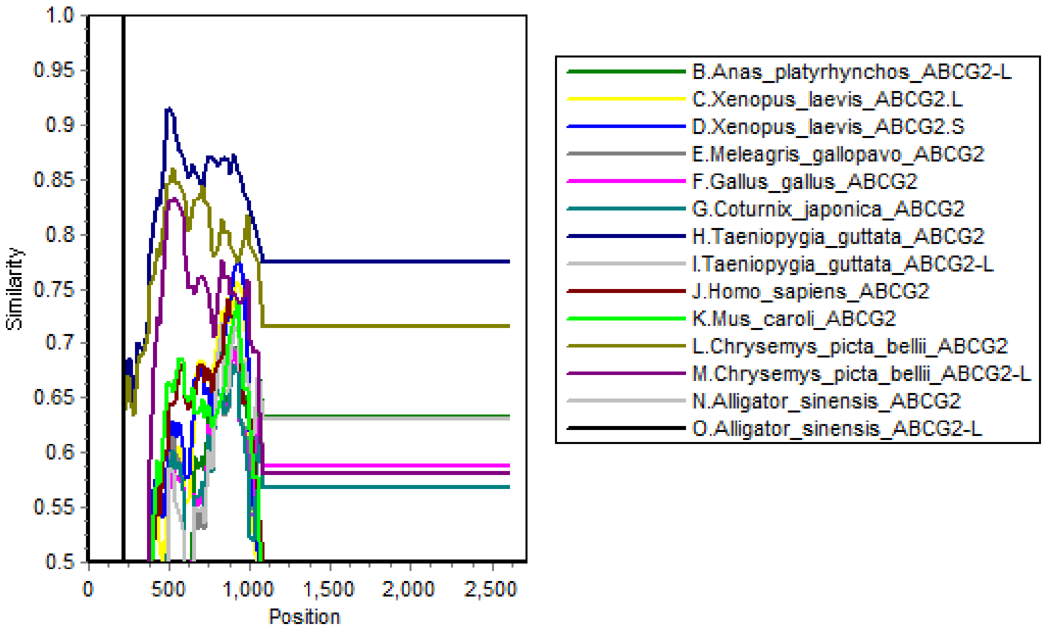


**Query *Anas platyrhynchos ABCG2-like***

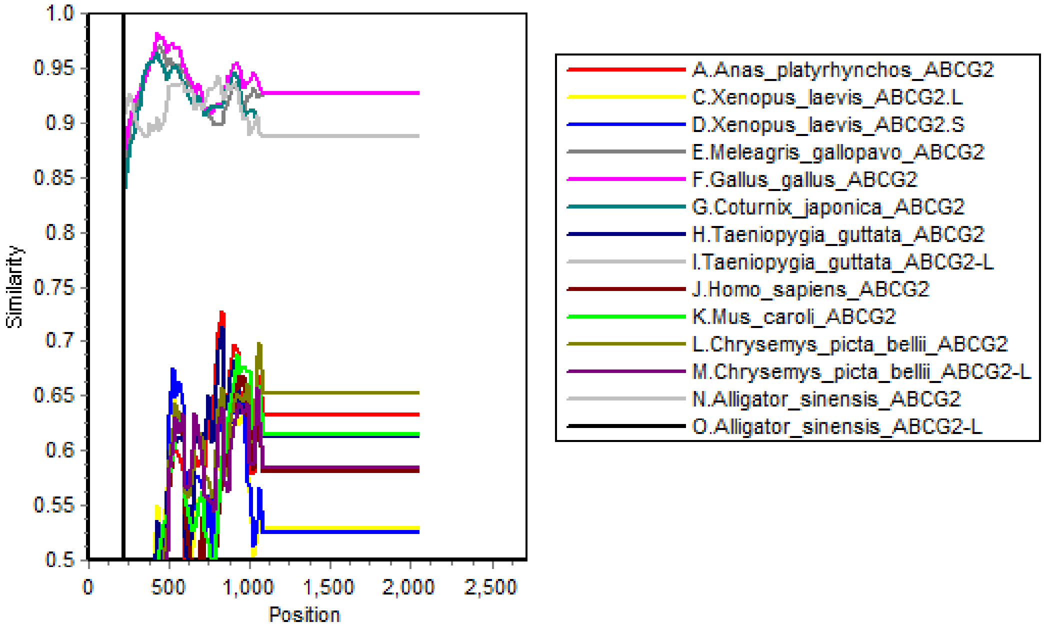

**Query *Meleagris gallopavo ABCG2***

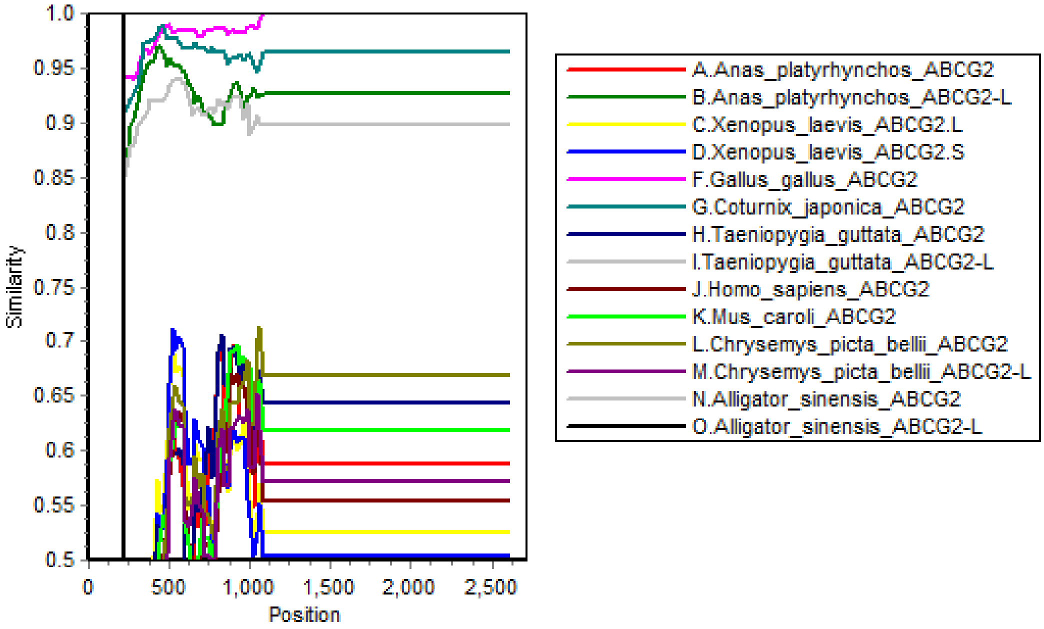


**Query *Gallus gallus ABCG2***

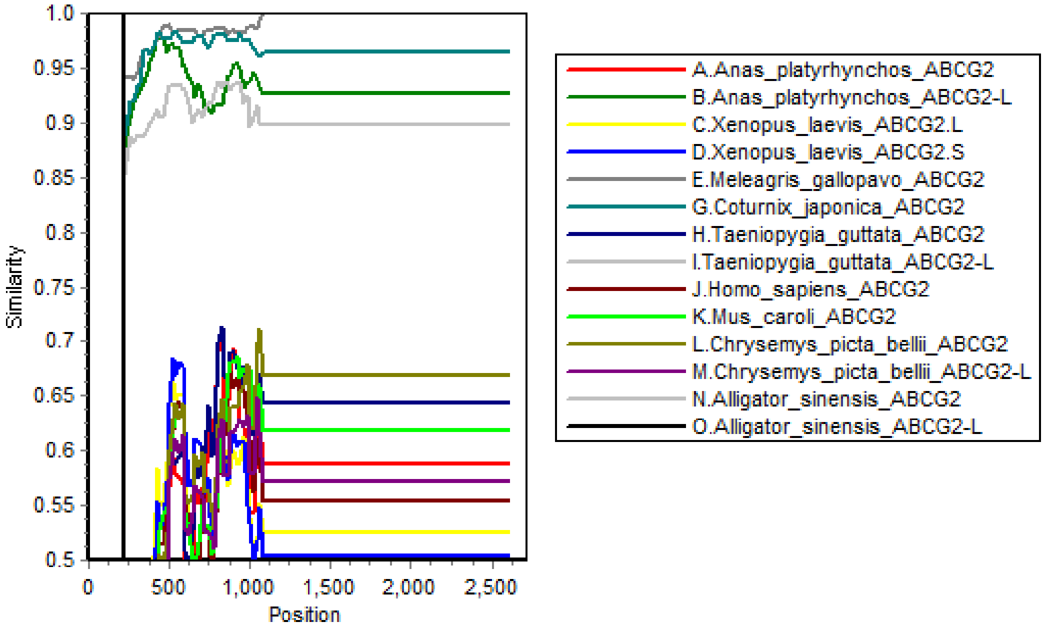

**Query *Coturnix japonica ABCG2***

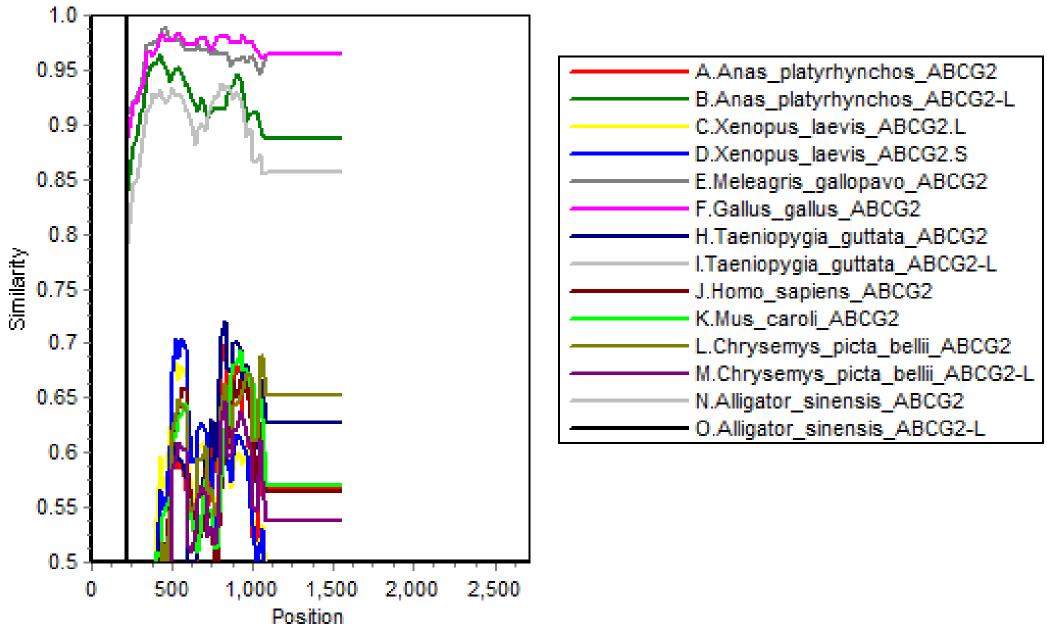


**Query *Taeniopygia guttata ABCG2***

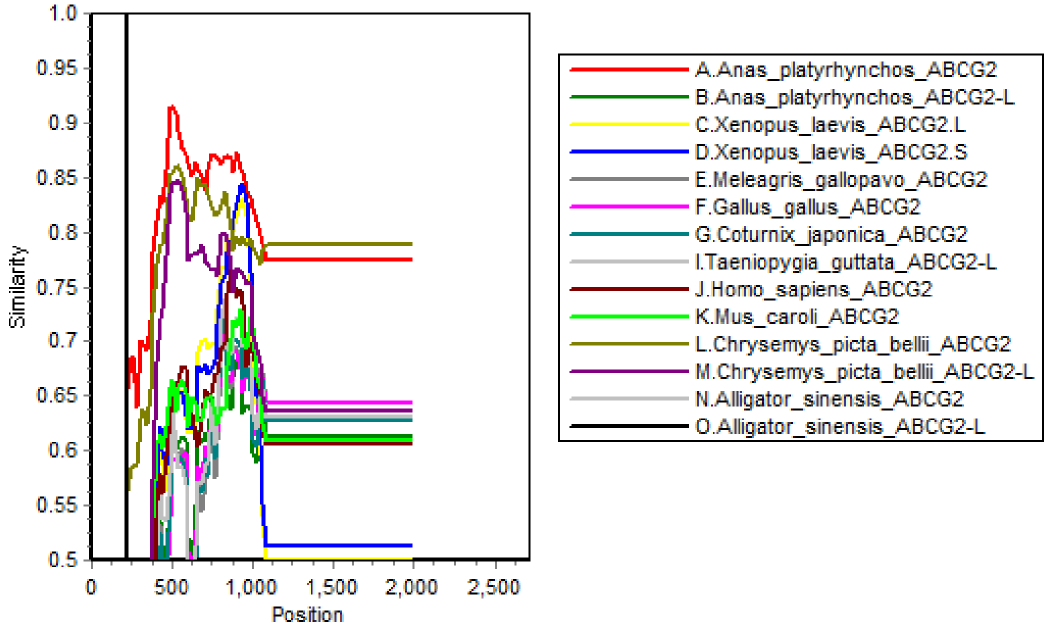

**Query *Taeniopygia guttata ABCG2***

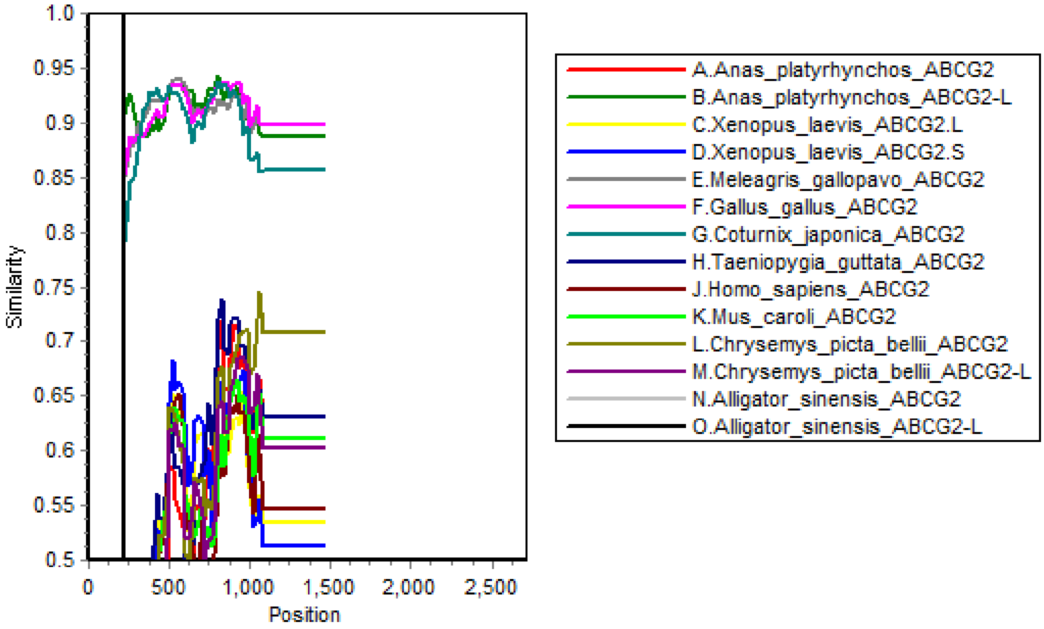

Supplement: Supplementary file 9 — Additional file 9. Sequence similarity plots of coding and pseudogene sequences of the ABCG2 and ABCG2-like gene families in birds and mammals. [file 12862_2020_1654_MOESM9_ESM.pdf]
